# optPBN: An Optimisation Toolbox for Probabilistic Boolean Networks

**DOI:** 10.1371/journal.pone.0098001

**Published:** 2014-07-01

**Authors:** Panuwat Trairatphisan, Andrzej Mizera, Jun Pang, Alexandru Adrian Tantar, Thomas Sauter

**Affiliations:** 1 Systems Biology Group, Life Sciences Research Unit, University of Luxembourg, Luxembourg, Luxembourg; 2 Computer Science and Communications Research Unit, University of Luxembourg, Luxembourg, Luxembourg; 3 Interdisciplinary Centre for Security, Reliability and Trust, University of Luxembourg, Luxembourg, Luxembourg; Technische Universität Dresden, Medical Faculty, Germany

## Abstract

**Background:**

There exist several computational tools which allow for the optimisation and inference of biological networks using a Boolean formalism. Nevertheless, the results from such tools yield only limited quantitative insights into the complexity of biological systems because of the inherited qualitative nature of Boolean networks.

**Results:**

We introduce *optPBN*, a Matlab-based toolbox for the optimisation of probabilistic Boolean networks (PBN) which operates under the framework of the *BN/PBN toolbox*. *optPBN* offers an easy generation of probabilistic Boolean networks from rule-based Boolean model specification and it allows for flexible measurement data integration from multiple experiments. Subsequently, *optPBN* generates integrated optimisation problems which can be solved by various optimisers.

In term of functionalities, *optPBN* allows for the construction of a probabilistic Boolean network from a given set of potential constitutive Boolean networks by optimising the selection probabilities for these networks so that the resulting PBN fits experimental data. Furthermore, the *optPBN* pipeline can also be operated on large-scale computational platforms to solve complex optimisation problems. Apart from exemplary case studies which we correctly inferred the original network, we also successfully applied *optPBN* to study a large-scale Boolean model of apoptosis where it allows identifying the inverse correlation between UVB irradiation, NFκB and Caspase 3 activations, and apoptosis in primary hepatocytes quantitatively. Also, the results from *optPBN* help elucidating the relevancy of crosstalk interactions in the apoptotic network.

**Summary:**

The *optPBN* toolbox provides a simple yet comprehensive pipeline for integrated optimisation problem generation in the PBN formalism that can readily be solved by various optimisers on local or grid-based computational platforms. *optPBN* can be further applied to various biological studies such as the inference of gene regulatory networks or the identification of the interaction's relevancy in signal transduction networks.

## Introduction

The Boolean network (BN) modelling framework was first introduced by Kauffmann in 1969 for the study of gene regulatory networks [1]. It has been widely applied to analyse the dynamics of different biological systems such as the gene regulatory network of the yeast cell cycle [2], T-cell signalling [3], signal transduction in the apoptotic pathway [4] and many more. For an overview see, e.g., [5], [6]. Despite its simplicity, the framework has been shown to be capable of modelling large-scale biological networks and providing meaningful biological interpretations, e.g., the attractors can be correlated to different cellular states [7]. Nevertheless, BNs only provide a very limited quantitative insight into biological systems due to their inherent qualitative nature of state and time.

In 2002, the probabilistic Boolean network (PBN) modelling framework was introduced by Ilya Shmulevich and colleagues for the modelling of gene regulatory networks [8]. PBNs combine the rule-based modelling of Boolean networks with uncertainty principles as described by Markov chains [8], [9]. The PBN formalism allows multiple Boolean functions to be assigned to a certain node with corresponding selection probabilities. This assignment forms a collection of Boolean networks (so-called *constituent networks*) that are being randomly chosen in accordance with their selection probabilities throughout the course of a simulation of the PBN. A constituent Boolean network determining the state transition of the PBN is randomly chosen at each epoch in an *instantaneously random PBN*, while the transition determining constituent Boolean network remains constant for a period of time until a binary random variable asks for a switch in a *context-sensitive PBN* [10]. Modelling with PBNs provides a quantitative understanding of biological systems. For example, interactive effects (so called *influences*) between certain genes [8] or average activities of certain genes given by steady-state probabilities [7] can be computed and expressed in quantitative terms.

Over the past years, PBNs have been widely applied to study various biological systems. For instance, Yu *et al.* inferred a gene regulatory network of the interferon pathway in macrophages from time-course gene expression data via the calculation of Coefficient of Determination (CoD) to determine the selection probability of each predictor function [11]. Using a similar approach, Ma *et al.* inferred a brain connectivity network from functional Magnetic Resonance Imaging (fMRI) data where the influence of each brain compartment in patients with Parkinson's disease could be determined [12]. In recent years, Flöttmann *et al.* modelled the regulatory processes taking place during the production of induced pluripotent stem cells by combining the interplay between gene expression, chromatin modification, and DNA methylation [13]. An extensive analysis on the PBN model of Flöttmann *et al.* suggests possible interventions on gene regulation which might be further developed into clinical applications. For more examples, see [6], [14], [15], [16], where, among others, PBN models for the pathogenesis of dengue viral infection and the transcriptional programming during *C. elegans* development, are discussed.

To facilitate the building of BN and PBN models as a representation of biological systems, several computational tools have been developed which can be applied for the optimisation and inference of models in a Boolean formalism. For instance, *CellNetOptimiser (CellNOpt a.k.a. CNO)* by Saez-Rodriguez *et al.* [17] was used for the inference of a signal transduction network from high-throughput sandwich immunoassay data [18]. *Dynamic Deterministic Effects Propagation Networks (DDEPN)* by Bender *et al.* allows for the reconstruction of signalling networks based on time-course experimental data [19]. Lähdesmäki and Shmulevich introduced *BN/PBN toolbox* [20], a Matlab-based toolbox which allows for the simulation, visualisation and analysis of BN and PBN models. *BN/PBN* toolbox also provides a pipeline for network inference in both the BN and PBN formalisms based on experimental measurements such as microarray data. The network inference process is performed via the calculation of the CoD by exploring the error size of a given Boolean function (or so-called *predictor*) compared to data. The state transition probabilities and the influences that determine the interactive effect for each pair of molecules (such as genes) are subsequently calculated. The *BN/PBN toolbox* was initially designed for the inference and analysis of gene regulatory networks [20]. However, it was also applied for the study of different biological systems such as the brain connectivity network as previously mentioned [12].

Based on the existing functionalities of the *BN/PBN toolbox*, we introduce *optPBN*, a Matlab-based optimisation toolbox for probabilistic Boolean networks. *optPBN* allows for a simple generation of PBN models from rule-based Boolean modelling. Prior biological knowledge such as known interactions in the network, which was not considered in the original *BN/PBN* toolbox, can additionally be integrated as inputs in terms of Boolean rules. *optPBN* facilitates the incorporation of experimental data to BN/PBN models in order to generate an integrated optimisation problem which can subsequently be solved by various optimisation solvers. In comparison to the *BN/PBN* toolbox, *optPBN* extends the functionality by allowing the identification of suitable Boolean rules in BNs and the determination of selection probabilities in PBNs based on experimental data and prior knowledge. Even though the optimisation pipeline of *optPBN* is rather simple and straight-forward, the results generated from *optPBN* retain meaningful qualitative and quantitative biological interpretations which are in accordance with the observed biological phenomena as captured in the experimental data.

In terms of functionality, *optPBN* can handle optimisation problems of networks characterised by various complexities. We offer a stand-alone version of *optPBN* toolbox which is suitable for solving simple optimisation problems, e.g., for small networks. For solving optimisation problems of complex biological networks such as the Boolean model of apoptosis from Schlatter *et al.* which comprises 86 nodes and 125 interactions [4], we also offer a grid-based optimisation pipeline of *optPBN* toolbox that operates on a large-scale computational platform such as the Grid'5000 [21]. Based on the results obtained from the optimisation of Schlatter's model in the PBN format, we quantitatively identified an inverse correlation between UVB irradiation, nuclear factor kappa-B (NFκB) and Caspase 3 activations, and apoptotic activity which could not be demonstrated in the original article due to the qualitative limitation of the Boolean network framework. In addition, we were able to estimate the relevancy of a newly introduced molecular interaction, i.e., the activation of NFκB by Caspase 8, by considering the value of fitted parameter sets and the sensitivity of parameters as indicated by parameter distributions.

## Method and Implementation

### Probabilistic Boolean networks

A probabilistic Boolean network (PBN) is a collection of Boolean networks in which a constituent network governs the state (activity) of a node (molecule) for a random period of time before another randomly chosen constituent network takes over [7]. Formally, a probabilistic Boolean network 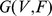 is defined by a set of binary-valued nodes 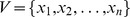 and a family of sets 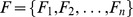. For each 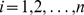 the set 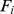 is 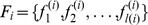 where 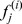 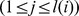 is a possible Boolean predictor function for the node 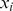 and 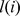 is the number of such predictor functions. A *realisation* of the PBN at a given instant of time is determined by a vector of predictor functions, where the *i*th element of that vector contains the function selected at that time point for 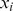. For a PBN with *N* realisations there are *N* possible network transition functions 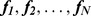 of the form 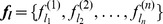, 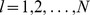, 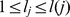, 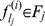 and 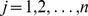. Each network transition function 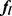 defines a constituent Boolean network of the PBN. In this way the realisations of the PBN can be identified with the constituent Boolean networks.

**Figure 1:**
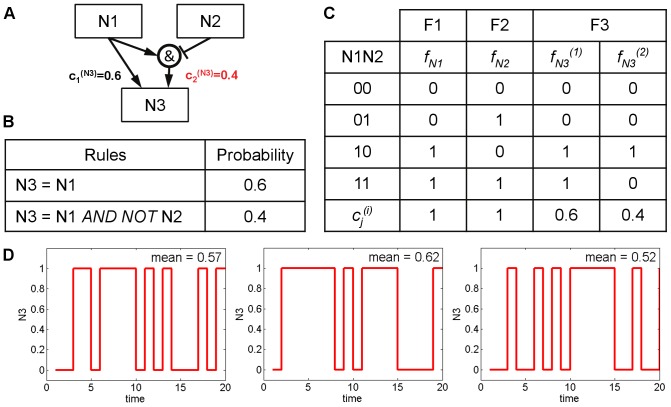
An example model with the corresponding Boolean rules, truth table and model simulation results. [A] The example model consists of 3 nodes with one activation edge and one partial inhibition edge. The weights of both edges are expressed as selection probability next to the arrow. [B] Two representative Boolean rules were assigned with the corresponding selection probabilities (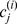) to represent the example model in PBN format. [C] The truth table of the example model demonstrates the state values according to different inputs. Once both inputs (N1 and N2) are active, the output (N3) has a probability of being ON at 0.6 and of being OFF at 0.4 according to the selection probability of Boolean rules. [D] Three separated Monte-Carlo simulations were performed on an instantaneously random PBN of the example model in Figure 1. The state values of N3 are shown on the y-axis as a function of time. The mean of the N3 state values over 20 time steps is given on the upper right corner of each run.

Let 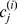 be the probability that the predictor 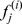, 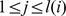, which is selected to determine the value of 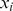 at the next time instance. It follows that 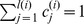. The PBN is said to be *independent* if the predictors for all nodes are selected independently of each other. Assuming independence, there are 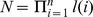 constituent Boolean networks of the PBN and the probability governing the selection of a particular network is given by 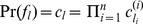 for all 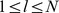. Two selection schemes are possible: the selection of the constituent Boolean network takes place at each consecutive time step (instantaneously random PBN) or there is a random variable which governs whether the PBN is updated in accordance with the current Boolean network or a newly selected one (context-sensitive PBN). In both cases the constituent network is chosen according to the selection probabilities 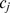, 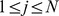. For further details on PBN, we refer to [6], [7] which give a comprehensive overview on probabilistic Boolean networks. An example of a PBN with three nodes is given in Figure 1.

The example model consists of three nodes V = (N1,N2,N3) and the functional classes F1 = {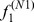}, F2 = {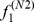}, and F3 = {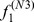,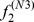}. N1 and N2 are inputs, where N1 activates N3 while N2 partly inhibits N3 (40%). The respective truth table is shown in Figure 1C. Once both N1 and N2 are activated (taking a state value of 1), node N3 could either solely be under the influence of N1 with a probability of 0.6, resulting in the activation of N3 that will take a state value of 1. Or, node N3 could also be under the influence of both N1 and N2 with a probability of 0.4, resulting in the inhibition of N3 that will take a state value of 0. The probabilistic terms that correspond to the selection probabilities (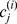) for the Boolean predictor functions are indicated in the truth table. We study this example in the context of instantaneously random PBNs and show three exemplary model simulations in Figure 1D.

There are two constituent Boolean networks of the example model given by the two different Boolean rules for node N3 shown in Figure 1B. These two constituent networks are randomly chosen at each time step of a simulation which for N1 = N2 = 1 results in flips of the state value of N3 between 0 and 1 as shown in Figure 1D.

**Figure 2:**
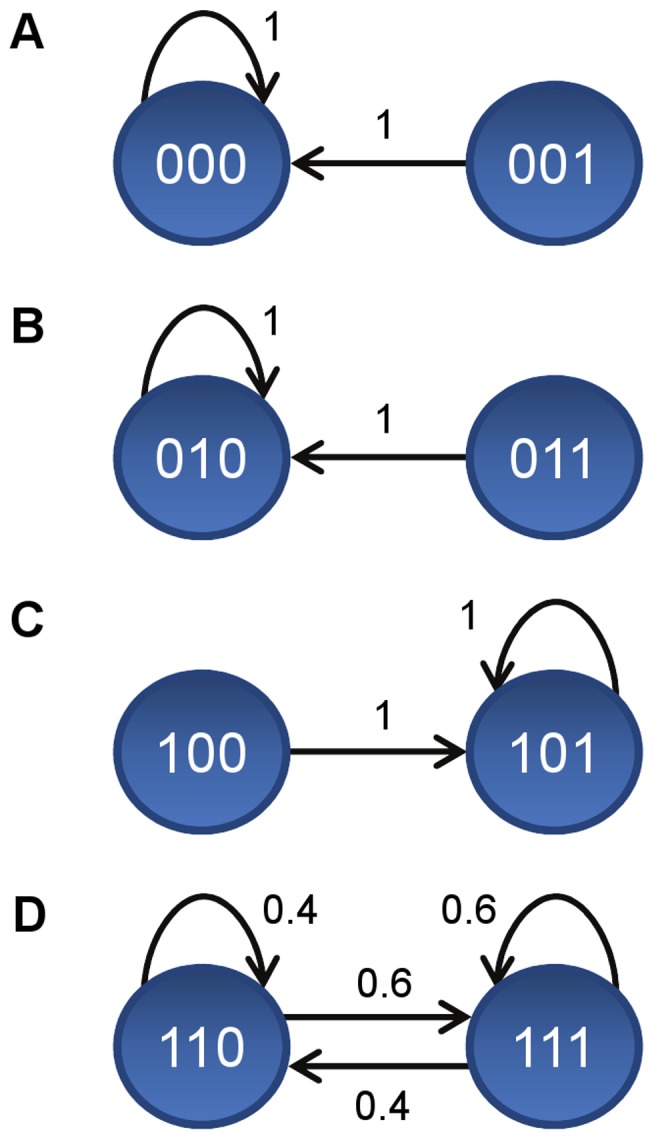
Structure of the dynamics of the example model. The dynamics of the example PBN model presented in Figure 1 is governed by a Markov chain. The graph of the Markov chain consists of four disjoint parts as presented in [A], [B], [C], and [D], respectively. In each graph, the nodes represent the states of the system and the possible transitions between the states are labelled with the respective transition probabilities. Four bottom strongly connected components of the graph that correspond to four irreducible subchains of the Markov chain are shown as follows: 000 [A], 010 [B], 101 [C] and 110, 111 [D].

The dynamics of the PBN is governed by a Markov chain which structure is presented in Figure 2. The nodes represent the states of the system and the possible transitions between the states are labelled with the respective transition probabilities. The graph of the Markov chain consists of four disjoint parts referred to as A, B, C, and D, respectively. There are four bottom strongly connected components of the graph that correspond to four irreducible subchains of the Markov chain: 000 (part A), 010 (part B), 101 (part C) and 110, 111 (part D). It follows that the Markov chain is not ergodic.

With N1 and N2 set to 1, the dynamics of the resulting PBN is given by part D of the Markov chain in Figure 2D which in fact is an ergodic two-state Markov chain. The steady-state probability for N3 to be active is 0.6. This value can be estimated by taking the mean activity over a Monte-Carlo run as shown in Figure 1D. The respective values obtained for 3 independent runs are 0.57, 0.62 and 0.52. In general, longer runs would result in a better estimation of the steady-state probability value.

From a biological point of view, the steady-state probability of a node being active can be interpreted as mean activity of the respective molecule in a cell population normalised to the maximal observed value. Let us assume that some *a priori* knowledge on the model structure is given in the form of a set of constituent Boolean networks, but the selection probabilities are unknown. The above biological interpretation provides basis for considering inferring the selection probabilities from measurement data from different biological conditions (e.g., different ligand stimulations, mutants, or inhibitor treatments). Once selection probabilities are inferred, the relevancy of Boolean interactions can be determined by the values of selection probability and the parameters sensitivity as indicated by their distribution. Furthermore, selection probabilities can be further used to calculate the influences, which reflect the relative importance of parent molecules on the target molecules in the resulting PBN [8].

### optPBN pipeline

**Figure 3:**
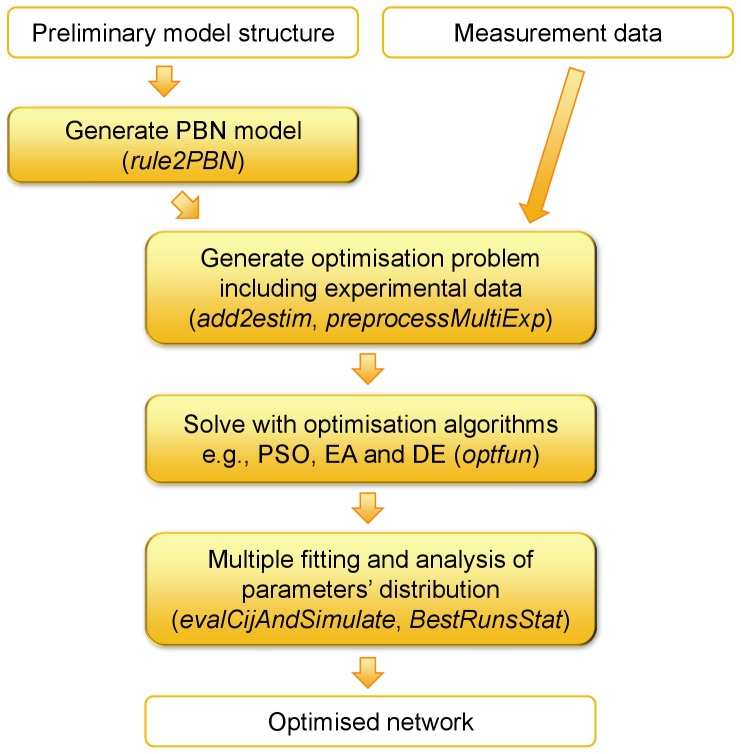
Optimisation pipeline of the *optPBN* toolbox. A preliminary model structure is required as an input for the generation of a PBN model. The generated PBN models from different experimental conditions together with the corresponding measurement data are subsequently combined to generate an integrated optimisation problem which can be solved by various optimisation algorithms. Once the optimisation algorithm(s) generate sufficient amount of good parameter sets, a statistical analysis of the optimised parameter sets (i.e., of PBN's selection probabilities) is performed to indicate the identifiability and the sensitivity of parameters through the consideration on parameters' distribution. The *optPBN* scripts used for each task are given in parentheses.

*optPBN* is a Matlab-based toolbox which operates under the framework of the *BN/PBN toolbox* by Lähdesmäki and Shmulevich [20], see File S1 for the toolbox and File S2 for *optPBN*'s examples. *optPBN* extends the existing functionalities of the original toolbox by allowing 1) for an easy BN/PBN models generation procedure allowing to incorporate prior knowledge, 2) for improved model fitting to multiple experimental data, i.e., the optimisation of selection probabilities for different experimental settings, 3) for a subsequent statistical analysis of the optimised parameters, and 4) for a fast computation on grid-based platforms. A simplified pipeline of the optimisation process in *optPBN* is shown in Figure 3 and a detailed explanation of the pipeline and computational scripts can be found in File S3.

The *optPBN* pipeline starts with the generation of a BN/PBN model from a preliminary model structure which is usually derived from literature. This step can be easily done by assigning different Boolean interactions in a rule-based Boolean modelling format for each molecule. This means, prior information is considered in terms of a set of possible constituent Boolean functions. For each molecule in a network, single or multiple Boolean functions with the corresponding selection probabilities can be assigned to define how often the respective Boolean function will be present in the chosen constituent network. For unknown or uncertain interaction(s), the selection probabilities of these Boolean rules can be inferred later by optimisation to normalised experimental data.

In the next step, an optimisation problem is generated based on the integration of the preliminary BN/PBN model structure and experimental data. The description of each experimental condition (e.g., different ligand stimulations, mutants, or inhibitor treatments) with its respective measurement data are defined as separate modelling cases. The integration step is simplified by applying the script *rule2PBN* to convert the rules and experimental description of each modelling case into the *BN/PBN* toolbox's internal variables (see the documentation in [20] or File S3) and by subsequently applying the script *add2estim* to collect and combine multiple modelling cases into a single global data structure named *estim*. Following this step, the script *preprocessMultiExp* derives only essential information to generate a final integrated optimisation problem which can subsequently be solved by different optimisation algorithms.

*optPBN* can be operated in two optimisation modes: ‘discrete’ and ‘continuous’. In the ‘discrete’ mode each Boolean network from the pool of considered networks is assigned one of two possible values: 0 or 1. Only Boolean networks with value 1 are considered as constitutive Boolean networks of the inferred PBN, each with equal selection probability. In the ‘continuous’ mode the selection probabilities can be any numbers in the range from 0 to 1 with the only constraint that the sum of selection probabilities of all constitutive Boolean networks of the inferred PBN is 1. Additional details on the two optimisation modes can be found in File S3.

To solve the integrated optimisation problem, two different sets of optimisers are used in *optPBN*: 1) particle swarm optimisation (PSO), *pswarmSB* [22], a global optimisation algorithm as described in the *Systems Biology Toolbox 2 (SBToolbox2)* [23], [24], and 2) the evolutionary algorithm (EA) [25] which is integrated in the population-based meta-heuristic optimisation framework *ParadisEO* [26] coupled with a differential evolution algorithm (DE) [27]. We therefore offer two versions of *optPBN*: a stand-alone version which uses *pswarmSB* and a grid-based version which uses the coupled EA and DE algorithms. For additional details on the pipeline of the grid-based version and the algorithms used, please see File S4.

The stand-alone version of the *optPBN* toolbox (PSO-based) was designed for solving simple optimisation problems, e.g., for small networks, while the grid-based version (EA- and DE- based) was customised to be implemented on a large-scale computational platform such as the Grid'5000 [21] for solving complex optimisation problems. The respective objective function of the optimisation process in *optPBN* pipeline is to minimise the sum of squared errors (SSE) between 1) molecular activities as represented by their steady-state probabilities and 2) measurement data in each experimental condition. The interface for communication between the integrated optimisation problem in *BN/PBN* toolbox's internal format and the optimisers, e.g., the conversion from sampled parameter values to selection probabilities (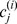) in PBN, is provided in the script *optfun* with a set of adjustable parameters to customise the optimisation process (see File S3).

During the optimisation process, we approximate the marginal steady-state distribution of the output nodes by applying the two-state Markov chain method as presented in the study of Shmulevich *et al.* [28]. The ergodicity of the PBN's underlying Markov chain is ensured by the introduction of perturbations controlled by a small perturbation parameter (p) as introduced by Miranda and Parga [29]. The two-state Markov chain method is subsequently applied to determine the number of simulation steps to be discarded before reaching steady-state (‘burn-in period’, *m*_0_) and the minimal number of time steps (*N*) required to estimate the marginalised steady-state distribution at a pre-defined accuracy. The accuracy of the steady-state approximation can be adapted by adjusting the parameters (ε, r, and s) as described by Raftery and Lewis [30]. Note that the equations used for the calculation of *m*_0_ and *N* as presented in [28] and [30] contain two small errors. We present the correct derivations of these two-state Markov chain approach formulas in File S5 and we applied the correct formulas in the *optPBN* pipeline accordingly.

Starting from an initial parameters setting (e.g. *m*_0_ = 0 and *N* = 100), we iteratively determine a new pair of values for *m*_0_ and *N* from the estimated transition probabilities between the two meta-states. If the new value for *m*_0_+*N* is greater than the previous value, the model is simulated further in order to extend the trajectory to the length given by the new value of *m*_0_+*N*. Then, the transition probabilities are re-estimated from the last *N* states in the trajectory and used to calculate new values for *m*_0_ and *N*. This process is repeated until the new value for *m*_0_+*N* is not greater than its previous value. Finally, the marginalised steady-state probability is estimated with the frequency with which the corresponding state in the two-state Markov chain was sampled within the *N* last elements of the obtained trajectory.

We observed in the investigated case studies that at least 5,000 iterations of selection probability (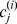) parameter sampling by optimisers for small models (n<10) and at least 7,500 iterations for large model (10<n<100) are sufficient to get a good fit and to obtain representative parameter sets for further statistical analysis. Note that this only holds for the investigated examples and cannot be generalised for other large-scale models. Once the optimisation process is finished, the best parameter set is reported and the model can be re-simulated with the script *evalCijAndSimulate* in order to check the quality of model fitting by comparing simulated steady-state probability to measurement data (see detailed explanation in File S3). Note that under the stochastic events of constituent networks chosen during PBN simulations, the same exact result might not be observed from the re-simulation. Nevertheless, the differences of the results between each simulation are expected to be minimal based on the assumption that the approximation of the steady-state distribution with the two-state Markov chain approach is rather accurate.

After checking model fitting, a representative set of parameters which fit well to measurement data can be chosen for further statistical analysis. The calculation of mean and standard deviation (SD) of the selected set of parameters can be performed by applying the script *BestRunsStat*. The mean of selection probabilities from selected parameter sets indicates to some extent what are the expected selection probabilities for the potential constitutive Boolean networks in PBNs that fit the experimental data. In parallel, the SD value gives an insight on the identifiability for each parameter and parameters' sensitivity can be assessed from parameter distributions. These pieces of information in turn allow for the estimation of the relevancy of Boolean interactions within the context of the study.

## Results and Discussion

In this section, four case studies with different levels of complexity are presented to demonstrate the functionalities of *optPBN*. We applied the *optPBN* pipeline, which approximates marginalised steady-state distribution with simulation of ergodic PBNs coupled with the two-state Markov chain method, to generate the results in this section. The parameters for checking steady-state convergence are set as follows: p = 0.001, r = 0.025, ε = 0.01 and s = 0.95.

**Figure 4:**
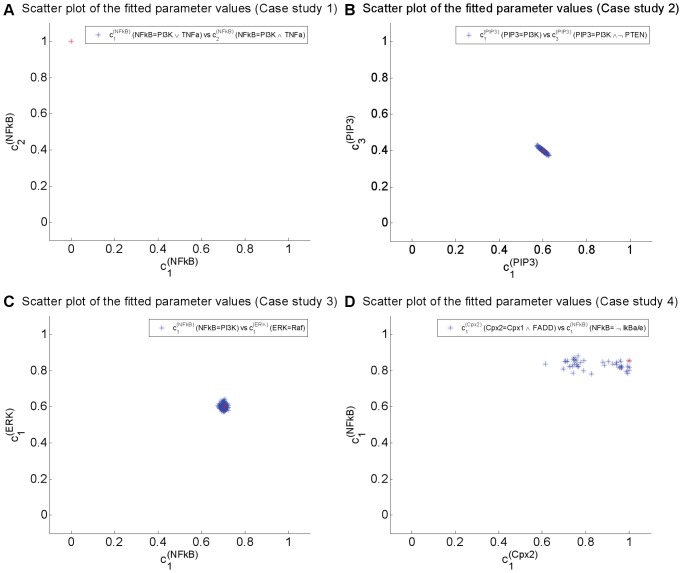
Scatter plots of a set of fitted parameters from all case studies. The distributions of selection probabilities from the best 500 parameter sets in term of optimal cost are shown in [A] for case study 1 (node NFkB), [B] for case study 2 (node PIP3). The dependency among selection probabilities across two nodes are shown in [C] for case study 3 (nodes NFkB and ERK) and [D] for case study 4 (nodes NFkB and complex2). The parameter values for the first 3 case studies form a single cluster which indicates that the respective parameters are identifiable. However, the parameter which influences on NFkB (y-axis) seem to be identifiable in case study 4 but the parameter which influences on complex2 (x-axis) are much sparser. Such observation raises an issue in term of parameters' identifiability. Only the best parameter set from each case study (marked as a red asterisk) was therefore used for further analysis and interpretation.

For each case study, we consider the best 500 parameter sets in terms of the optimal cost to analyse the identifiability of the model parameters and to perform subsequent statistical analyses. The spread of the identified parameters for each case study is shown in Figure 4. The scatter plots show that the obtained parameters are clustered for the first three case studies. However, the parameters in case study 4 are not always clustered. Therefore, we demonstrated the result generated from the best run of each case study which was marked as a red asterisk on Figure 4. A summary of these results can be found in File S6.

### Case study 1: optPBN allows for the identification of suitable Boolean rule(s) in Boolean networks

With respect to optimising qualitative Boolean networks, *optPBN* is capable of identifying a set of suitable Boolean rules from a user-defined list of candidate rules based on experimental data. For this task, *optPBN* is operated in the ‘discrete mode’ for which only 0 and 1 values for the selection probabilities are evaluated (see detail in File S3).

**Figure 5:**
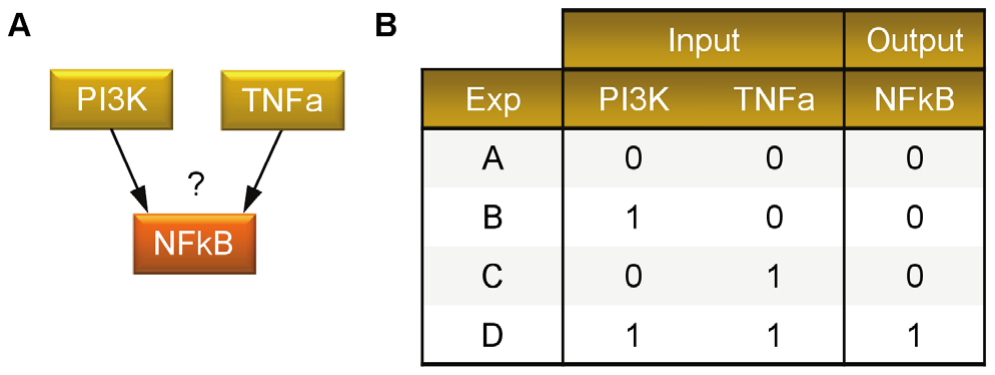
Case study 1. [A] Case study 1 deals with a Boolean network that consists of 3 nodes with an unknown Boolean interaction from the two inputs. [B] The table contains artificial experimental data from four different combinations of input states (Experiments ‘A’, ‘B’, ‘C’, and ‘D’) of case study 1.

In order to demonstrate the respective functionality of *optPBN*, we use case study 1 (Figure 5) as an example. We pre-define a set of five different Boolean rules to represent the potential influence of PI3K and TNFa on NFkB as follows: connect PI3K and TNFa activation with an OR gate (|), connect PI3K and TNFa activation with an AND gate (&), has only an activation from PI3K (PI), has only an activation from TNFa (TN), and has no interactions from either PI3K or TNFa and output is fixed to 0 (Ø). We consider 4 experimental measurements of NFkB, each with a configuration of the input nodes PI3K and TNFa. For each individual measurement, we applied *optPBN* to determine which of the pre-selected rules(s) is capable of explaining the experimental data. The obtained results show that *optPBN* could identify the correct Boolean rules. Then, we applied *optPBN* to all experimental measurements considered simultaneously. In this case, *optPBN* identified the connection of PI3K and TNFa to NFkB with an AND gate (&) as the only suitable Boolean rule which can explain the complete set of experimental data. This case study shows that *optPBN* can be applied for the inference of biological networks in the Boolean formalism. The obtained results are summarised in Table 1.

**Table 1:** Results from the *optPBN* toolbox for case study 1 compared to the original network.

Optimisation results					
Exp|rules	I	&	PI	TN	Ø
A	✓ (0.2)	✓ (0.2)	✓ (0.2)	✓ (0.2)	✓ (0.2)
B	✗ (0)	✓ (0.33)	✗ (0)	✓ (0.33)	✓ (0.33)
C	✗ (0)	✓ (0.33)	✓ (0.33)	✗ (0)	✓ (0.33)
D	✓ (0.25)	✓ (0.25)	✓ (0.25)	✓ (0.25)	✗ (0)
**All (A to D)**	**✗ (0)**	**✓ (1)**	**✗ (0)**	**✗ (0)**	**✗ (0)**

Original network					
**Correct rule**	**✗ (0)**	**✓ (1)**	**✗ (0)**	**✗ (0)**	**✗ (0)**

The table shows the results of optimisation for four different individual datasets (A, B, C and D) and for the combined four datasets (All) compared to the original network. Five different Boolean rules are applied as follows: connect PI3K and TNFa activation with an OR gate (|), connect PI3K and TNFa activation with an AND gate (&), has only an activation from PI3K (PI), has only an activation from TNFa (TN), and has no interactions from either PI3K or TNFa and output is fixed to 0 (Ø). The symbol ‘✓’ indicates that the respective rule can explain the measurement data while the symbol ‘✗’ refers to the contrast observation. The results from the *optPBN* toolbox divide the sum of probabilities, i.e. 1, by the number of correct result(s) in each experiment (given in parentheses) and they all correspond to the correct results.

### Case study 2: optPBN allows for the determination of selection probabilities in probabilistic Boolean networks

In this theoretical case study, we consider the regulation of PIP3 by PI3Kand PTEN. We assume that this process can be modelled with the network presented in Figure 1A, where the nodes N1, N2, and N3 represent PI3K, PTEN, and PIP3, respectively. Nodes N1 and N2 are the so-called input nodes, i.e., they are not influenced by any node in the network and their values are determined by explicit assignment. This makes that the underlying Markov chain consists of four disjoint, non-communicating Markov subchains, one for each of the four different assignments of values to the input nodes.

**Figure 6:**
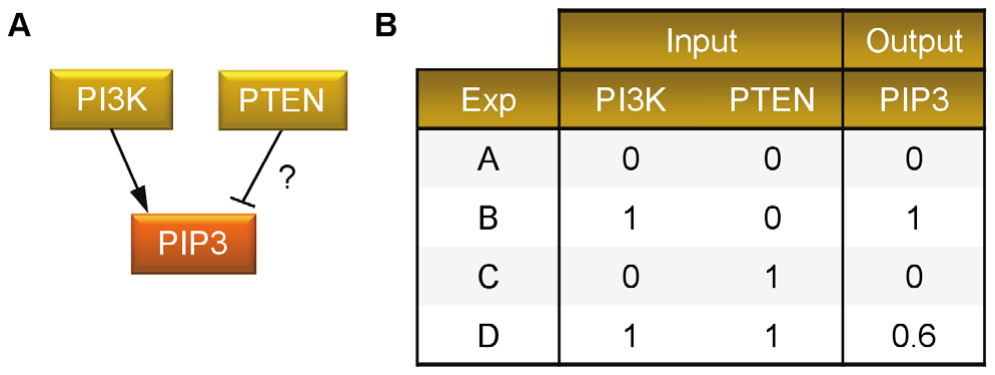
Case study 2. [A] Case study 2 deals with a probabilistic Boolean network that consists of 3 nodes with an unknown type and weight of interaction from PTEN to PIP3. [B] The table contains artificial experimental data from four different combinations of input states (Experiments ‘A’, ‘B’, ‘C’, and ‘D’) of case study 2.

Let us now assume that the model structure is only partially known, i.e., it is known that PI3K activates PIP3, but there is no certain information on whether PTEN activates or inhibits PIP3 and to what extent. Therefore, as a prior knowledge, we consider four different Boolean rules that encode four potential signal flows from PI3K and PTEN to PIP3 as follows: only activation from PI3K (PI), only activation from PTEN (PT), activation from PI3K and inhibition from PTEN (PI&∼PT), and no interaction from either PI3K or PTEN and output is 0 (Ø). Furthermore, four experiments are performed, where various combinations of values for PI3K and PTEN as the initial conditions are considered. As the measured values of PIP3 we take the theoretical values of the underlying Markov chain stationary probabilities determined by the initial conditions. The partially known network structure and the experimental data are shown in Figure 6. Now, we applied *optPBN* to perform the optimisation in the ‘continuous mode’ where an extensive continuous parameter space (the interval from 0 to 1) is explored within the optimisation process to determine the selection probabilities for the four different Boolean rules.

When performing optimisation, the values of the input nodes are fixed to the values specified by the available experimental conditions, one by one. For each experimental dataset only the subchain determined by the experimental condition is considered. In order to make the considered part of the underlying Markov chain ergodic, perturbations are introduced which make the considered subchain irreducible and aperiodic. In this way, the considered part has a unique steady-state probability distribution which can be estimated by the two-state Markov chain approach independently of the choice of the initial state of a simulation. The obtained steady-state probabilities are estimated and the squared difference of the estimated value and the experimental value is calculated. To get the final fit score, the squared differences from all experimental conditions are added.

Two remarks are in place. First, it should be noted that the fit quality of these experiments could be improved by increasing the accuracy for the approximation of steady-state distribution, e.g., by adjusting the parameter ‘r’. Details on model fitting's quality in relationship to the accuracy parameter ‘r’ can be found in File S6. Second, more importantly, the inference results heavily depend on the experimental data. In this case study the set of experimental data was comprehensive in the sense that it covered possible assignments of values to the input nodes: by considering experiments A, B, C, and D in Figure 6 part [B], all the four non-communicating subchains of the Markov chain in Figure 2 are taken into account. If this is not the case, the inference may result in wrong outcomes. According to the results of this case study shown in Table 2, the selection probabilities inferred from all experiments agree well with the selection probabilities of the original network. However, when only experimental data from experiments A and D were taken into account, the optimisation inferred a PBN consisting of all four constituent Boolean networks given by the rules ‘PI’, ‘PT’, ‘PI&∼PT’,and ‘Ø’, with selection probabilities 0.4602, 0.1344, 0.3607 and 0.0447, respectively.

**Table 2:** Results from the *optPBN* toolbox for case study 2 compared to the original network.

Optimisation results				
Exp|rules	PI	PT	PI&∼PT	Ø
A,D	0.4602	0.1344	0.3607	0.0447
**All (A to D)**	**0.6041**	**0**	**0.3959**	**0**

Original network				
**Selection probabilities**	**0.6**	**0**	**0.4**	**0**

The table shows the results of optimisation for two datasets: 1) containing measurement data from experiments A and D 2) containing all measurement data from experiments A, B, C and D. Four different Boolean rules are applied as follows: only activation from PI3K (PI), only activation from PTEN (PT), activation from PI3K and inhibition from PTEN (PI&∼PT), and no interaction from either PI3K or PTEN and output is fixed to 0 (Ø). The selection probabilities inferred from all experiments agree well with the selection probabilities of the original network. The dataset consisting of the measurement from only experiments A and D is insufficient to reconstruct the original network.

In summary, this case study demonstrates that the *optPBN* toolbox can be applied to infer selection probabilities from given comprehensive data. Once the selection probabilities are obtained, they can subsequently be used to estimate the relevancy of Boolean interactions. In addition, they can also be used to determine the influence between molecules as presented in [8].

### Case study 3: optPBN generates comparable results to an existing tool while having a broader functionality

To date, there are several computational tools which are applicable for the optimisation of biological networks in the Boolean formalism. One of the leading tools is *CellNetOptimizer (CellNOpt* a.k.a. *CNO*) introduced by Saez-Rodriguez *et al.* [17]. CellNOpt was applied for building logic-based models of signal transduction networks in different logic formalisms that are trained against high-throughput proteomics data [18].

To illustrate and prove the functionalities of *CellNOpt*, the tool was used to optimise a toy model based on a set of artificial experimental data. The objective function of *CellNOpt* is based on two components: 1) the mean squared error (MSE) deviation between data and predicted states, and 2) a penalised term for increasing model size (È_s_) which is adjustable by a scaling factor (α). By minimising a combination of these two terms, *CellNOpt* was able to identify the Boolean interactions that correspond to experimental data (see Figure S1).

To benchmark our newly developed toolbox, we applied *optPBN* to optimise the compressed version of the toy model in Boolean formalism as presented in the original *CellNOpt* article [18], see also Figure S1. The original toy model comprises 8 nodes. There are 2 input nodes which are TGFa and TNFa with two downstream nodes that can be inhibited by inhibitors (PI3Ki and Rafi). The presence of inhibitor is depicted in the input table with ‘−’ once it is absent and with ‘+’ when it is present. The rest of the nodes are considered as output nodes and are all measured. Here, we applied *optPBN* to optimise the two unknown logic gates for NFkB and ERK.

The original model structure with 6 different experimental conditions were plugged into the *optPBN* pipeline in ‘discrete mode’ as previously described for case study 1. *optPBN* is capable of acquiring the same results as *CellNOpt*, i.e. to identify ‘NFkB = PI3K & TNFa’ and ‘ERK = Raf’ as the correct Boolean rules. In addition, *optPBN* also identified the rule ‘ERK = Raf|NFkB’ as an additional solution that can also explain the data from all experimental conditions (see Figure S1). We also verified that both correct Boolean rules are independent as the optimal costs after assigning these two rules one-at-a-time to be the correct rule are highly similar.

**Figure 7:**
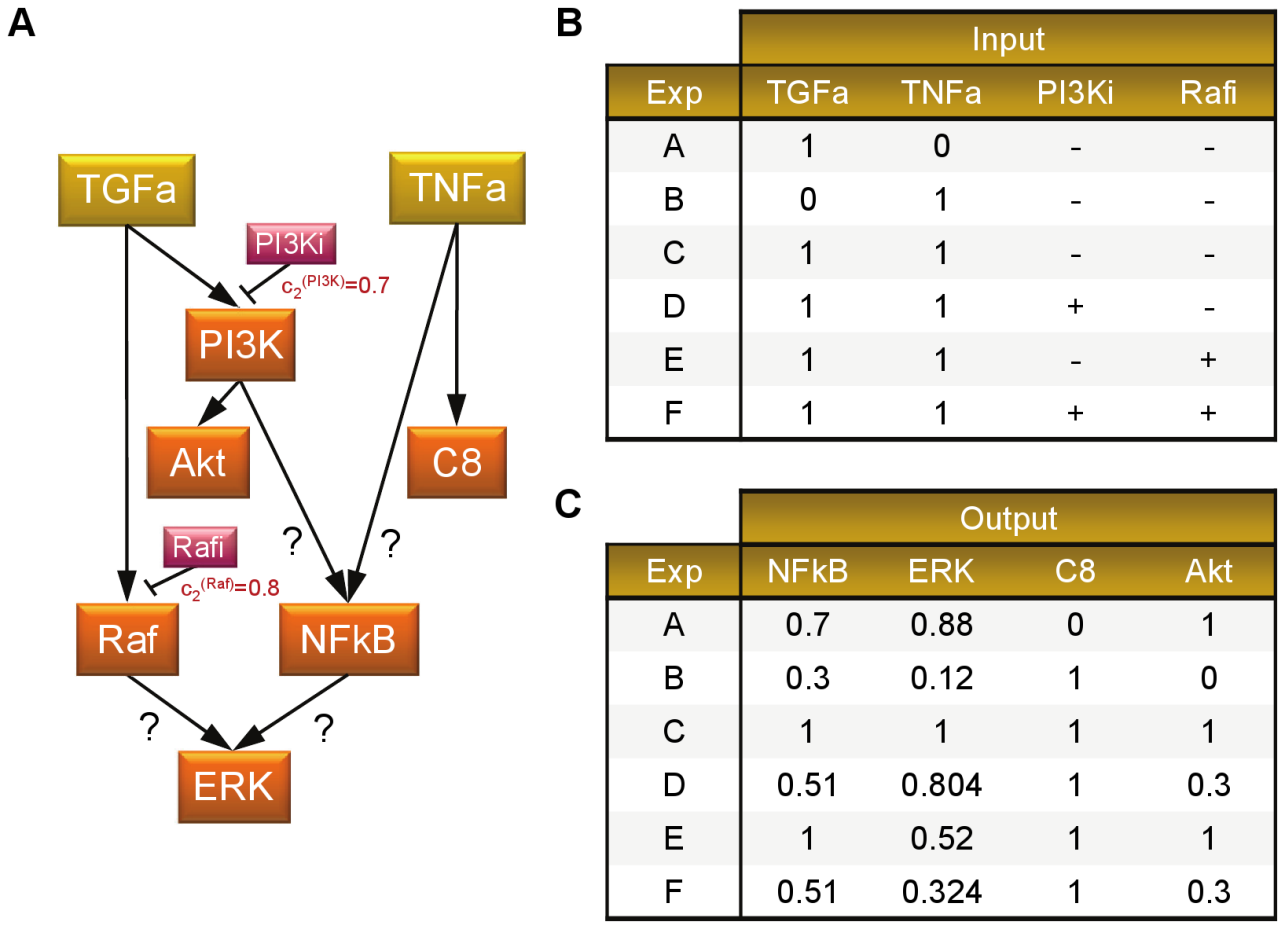
Modified toy model of Saez-Rodriguez *et al.* and corresponding artificial experimental data (case study 3). [A] The modified toy model from Saez-Rodriguez *et al.* [18] is a probabilistic Boolean network that consists of 8 nodes with two unknown weights of Boolean interactions for NFkB and ERK. [B] The table describes the states of inputs and inhibitor treatments for 6 experimental conditions. [C] The corresponding normalised artificial experimental data of the experimental conditions as described in [B]. The 6 experimental conditions based on the combination of stimulus and inhibitor treatments yield different readouts on four downstream molecules.

Then, we extended the current study by applying *optPBN* for the optimisation of a modified toy model based on a new set of artificial data (case study 3) as shown in Figure 7. In this version, we assumed that the weights of molecular activation and the inhibitors' efficacies are not absolute, resulting in a propagation of signals in a non-discrete (continuous) manner. Once output nodes can be activated by multiple molecules, i.e., NFkB can be activated by PI3K and TNFa while ERK can be activated by Raf and NFkB, we consider disjoint activating signals from both inputs which are sum up to a full activation. When inhibitor is additionally present, the activating signal is reduced proportionally to the inhibitor's efficacy.

Considering e.g. experiment D, both inputs are ON and there are two unknown weights of activation from PI3K and TNFa towards NFkB with the presence of PI3K-inhibitor treatment. If we assume that the activating signal from PI3K is 70% and from TNFa is 30% with the presence of PI3K-inhibitor that inhibits PI3K signal at 70%, the signal for the activation on NFkB in this experiment can be calculated from the sum of the remaining PI3K signal after inhibition (100%−70% = 30%) multiplied by the weight of PI3K's activation (70%), resulted in the signal value of 0.3*0.7 = 0.21. This signal is then combined with the disjoint activating signal from TNFa (30% or 0.3). The sum of activating signals for NFkB node is therefore 0.51 in this experimental setting.

To perform an optimisation study on this modified toy model, *optPBN* was applied in the ‘continuous mode’ as previously described for case study 2. The optimisation results as shown in Table 3 are in a good agreement with the selection probabilities of the original model.

**Table 3:** Results from the *optPBN* toolbox for case study 3 compared to the original network.

Optimisation results				
Outputs	NFkB		ERK	
Exp|rules	PI	TN	RA	NF
**All (A to F)**	**0.708**	**0.292**	**0.603**	**0.397**

Original network				
**Selection probabilities**	**0.7**	**0.3**	**0.6**	**0.4**

The table shows the optimisation results for the dataset which combines all six experiments from A to F (All) in case study 3. Two different Boolean rules are applied for NFkB and another two rules for ERK as follows: activation signal from PI3K (PI) or TNFa (TN) to NFkB, and activation signal from Raf (RA) or NFkB (NF) to ERK. The results from the optimisation of modified toy model from Saez-Rodriguez *et al.* are corresponded with the correct results.

In summary, the results from the two toy model studies demonstrate that the optimised networks generated from *CellNOpt* and *optPBN* are similar when operated in a discrete (qualitative) optimisation mode. At the same time, *optPBN* offers an additional functionality of a continuous (quantitative) optimisation mode to identify selection probabilities which might yield additional insight into the relevancy of interactions within the network.

### Case study 4: optPBN allows for the optimisation of an apoptotic network at scalable computational time and for the estimation of interactions' relevancy in a context-specific manner

#### Optimisation of an apoptotic network in the PBN framework

**Figure 8:**
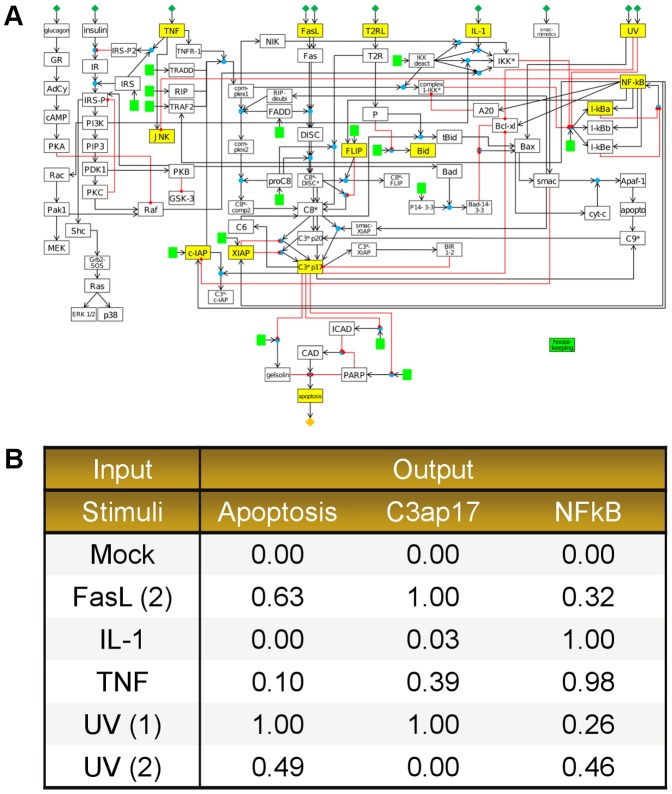
Boolean model of apoptosis by Schlatter *et al.* and normalised experimental data (case study 4). [A] The Boolean model of apoptosis by Schlatter *et al.* [4] consists of 86 nodes with 125 Boolean interactions. The model was analysed with *CellNetAnalyzer (CNA)* to study the correlations between 10 different inputs from different cytokines stimulations and UVB irradiations towards apoptosis. [B] The normalised experimental dataset were generated based on the experimental data as presented in the original article (see details in File S7). The inverse correlation between UVB irradiation, NFκB and P17 form of activated Caspase 3 (C3ap17) activations, and apoptotic activity is quantitatively observed in the original measurement data.

Schlatter *et al.* introduced a large-scale Boolean network of apoptosis in hepatocytes that consists of 86 nodes and 125 interactions as shown in Figure 8. [4] The assigned Boolean interaction for each molecule was derived from literature. After the Boolean model was built, it was subsequently validated by experimental data which were categorised into three discrete values: no activity ‘0’, low activity ‘1’, and high activity ‘2’. The analysis of Schlatter's model was conducted in *CellNetAnalyzer (CNA)*, a Boolean network and constraint-based models analyser which allows for the calculation of logical steady-states [31]. As the original model structure comprises many feedback loops, 13 interactions were removed from the model in order to generate a new model variant which delivers fixed point steady-states and thus is compatible to be analysed in CNA. The analysis revealed the effects from different cytokines stimulations and UVB irradiations towards apoptosis in hepatocytes, but only in a limited qualitative manner [4].

Based on the original study, we applied *optPBN* to optimise Schlatter's model in the PBN formalism. We converted the multi-value Boolean model of apoptosis into a binary PBN model which comprises 96 nodes and 106 interaction functions (‘initial apoptosis model’). We used the selected set of Boolean interactions as described in the original article with minimal modifications on a few Boolean rules to make them suitable for modelling in the PBN format (see File S2). Our initial aim is to optimise selection probabilities of our PBN model in order to return the steady-state probabilities of 3 output nodes, i.e., Apoptosis, Caspase 3 and NFkB, which match the measurement data. We optimised the selection probabilities of Boolean rules for 7 target nodes: IKK, IkBa, IkBe, complex2, caspase8 and caspase3 (both at low and high activities), which are connected to the 3 output nodes. This results in the optimisation of 17 selection probabilities.

Next, pre-processing of the original measurement data on hepatocytes for the three output nodes: Apoptosis, Caspase 3 and NFkB, was performed by background subtraction and normalisation to the maximal value. Saturation of Caspase 3's signals was assumed in our study (see File S7). Then, the normalised experimental data and the PBN model description were combined into an integrated optimisation problem which was subsequently solved with *optPBN* in ‘continuous mode’.

For this case study six different experimental conditions which were experimentally validated are given. During optimisation six subchains of the underlying Markov chain are considered, each determined by fixing an input node's value in accordance with the experimental condition. The fixed value of the input node is not perturbed, but all the other nodes can be perturbed, which makes the subchain ergodic and in consequence having a unique steady-state distribution. It should be noted that performing the optimisation with use of a more rich set of experimental data, i.e., for conditions which correspond to setting the input nodes to different combinations of values, could provide more insight into the network interactions.

#### Optimisation of a complex network at scalable computational time

To evaluate the fitting cost during the optimisation process, the marginalised steady-state probabilities for activity of one molecule at a time for a set of output nodes (Apoptosis, Caspase 3 and NFkB) needed to be estimated. As previously presented in the description of the *optPBN* pipeline, this was achieved by applying the two-state Markov chain approach with the accuracy set to 0.025 (r = 0.025). Although the size of the underlying Markov chain of the PBN is huge, i.e., 2^96^ states, it turned out that in all three cases (Apoptosis, Caspase 3 and NFκB) the two-state Markov chains were well-mixing: there were frequent transitions between the two meta-states of the two-state Markov chains that were considered on top of the underlying Markov chain of 2^96^ states. In consequence, the marginalised probabilities could be estimated from trajectories of length less than 4000 (see File S6). Given this, we were able to perform the optimisation task in a feasible amount of computational time.

To confirm the accuracy of our results, we performed additional analyses by fixing one set of selection probabilities (randomly generated) and started the simulation from random initial conditions as well as from the extreme cases where initial conditions for all nodes are either zero or one. We found that the variations between these 3 cases are minimal, all less than 0.01 (1%). Also, we generated another set of results with higher accuracy (r = 0.01) where the number of required time steps is increased approximately 6 folds. We found that the steady-state distributions of output nodes which were estimated at the two levels of accuracy (r = 0.025 and r = 0.01) are almost the same, i.e., they differ by less than 0.01 (1%) in all case. This indicates that the chosen parameters lead to a good estimate of the steady-state distribution of the considered model. We also performed the same analysis multiple times for different parameter sets where we obtained similar results. We present a comprehensive datasheet of the analysis performed for one parameter set in File S8.

Due to the large size of the apoptotic network (Figure 8), the stand-alone version of *optPBN* pipeline which runs on a standard local computer (1 CPU Intel@2.99 GHz, 2 cores/CPU, 3.25 GB RAM) is not suitable to solve the optimisation problem. Since the optimisation required 12 hours of computational time to evaluate approximately 5,000 parameter samples on a standard computer, we applied the grid-based version of *optPBN* to solve this optimisation problem on the Grid'5000 using 10 server machines (1 server machine comprises 2 CPUs Intel@1.995 GHz, 4 cores/CPU, 15 GB RAM, see detailed documentation on the installation and execution of the grid-based pipeline in File S4 and File S9). The optimisation process was operated in the ‘continuous mode’ on 80 parallel processing cores where the results were delivered in a timely manner (approximately 10 minutes to evaluate 15,000 parameter samples). The run-time analysis of four case studies reveals a reduction of computational time from 35 to 175 folds when running the same optimisation tasks on Grid'5000 with 80 cores. More details on this analysis are shown in Table 4.

**Table 4:** Run-time analysis of grid-based *optPBN* pipeline.

Model	Iterations	Stand-alone version (1 core)	Grid-based version (80 cores)	Improvement (folds)
1	1000	305s (5m5s)	8s	38.1
	5000	1093s (18m13s)	16s	68.3
2	1000	321s (5m21s)	9s	35.7
	5000	1205s (20m5s)	16s	75.3
3 (modified model)	1000	1302s (21m42s)	18s	72.3
	5000	6151s (1h42m31s)	39s	157.7
4 (extended structure)	1000	16783s (4h39m43s)	99s (1m39s)	169.5
	5000	45503s (12h38m23s)	259s (4m19s)	175.7

The table shows the computational time of the optimisation process required by the grid-based version of *optPBN* pipeline operated on 80 cluster cores in comparison to the one required by the stand-alone version running on a single local machine. The run-time analysis was performed on the four case studies for 1,000 and 5,000 parameter samplings to approximate the steady-state probability distribution of output nodes. The results reveal a remarkable reduction of the computational time from 35 to 175 folds. Abbreviations: s = seconds, m = minutes, and h = hours.

#### Approximation of steady-state distributions by optPBN reveals more quantitative insight into biological data

For comparison, the original results from Schlatter's study (Orig.) and the set of results from the optimisation of the ‘initial apoptosis model’ by *optPBN* (Init.) are shown in Table 5. We were able to identify a PBN model structure with a set of selection probabilities that could match relatively well to the measurement data quantitatively, while the results from the original model are only limited to 0 and 1 value. The fitting costs of the original model and of the ‘initial apoptosis model’ based on the calculation of SSE are 1.002 and 0.328, respectively. This indicates that the apoptosis model in the PBN formalism fits the measurement data better.

**Table 5:** Original and *optPBN* results for the Boolean model of apoptosis (case study 4).

	Apoptosis				C3ap17				NFkB			
Exp	Orig.	Init.	Ext.	Meas.	Orig.	Init.	Ext.	Meas.	Orig.	Init.	Ext.	Meas.
Mock	0	0.0000	0.0054	0.00	0	0.0168	0.0082	0.00	0	0.0168	0.0027	0.00
FasL (2)	1	0.9208	0.7170	0.63	1	0.9505	0.8491	1.00	0	0.0198	0.1501	0.32
IL-1	0	0.0028	0.0063	0.00	0	0.0042	0.0013	0.03	1	0.8873	0.8481	1.00
TNF	0	0.0020	0.0018	0.10	0	0.4073	0.4049	0.39	1	0.7889	0.8723	0.98
UV1	1	0.9920	0.9868	1.00	1	0.9966	0.9901	1.00	0	0.0023	0.0033	0.26
UV2	0	0.4681	0.4914	0.49	0	0.0016	0.0012	0.00	1	0.3083	0.2932	0.46

The results from logical steady-state analysis in *CellNetAnalyzer* of the original Boolean model from Schlatter *et al.* [4] (Orig.) are shown in comparison to the *optPBN* results generated from the ‘initial apoptosis model’ (Init.), a probabilistic Boolean network (PBN) model which was modified from the original Boolean model, and from the ‘extended apoptosis model’ (Ext.), a PBN model variant with an additional literature-derived Boolean interaction from Caspase 8 to NFκB. The sum of optimal costs based on the calculation of sum of square error between simulated steady-state probabilities and measurement data (Meas.) are 1.002, 0.328, and 0.199, respectively. The inverse correlation between UVB irradiation (UV1 = 300 £/m^2^, UV2 = 600 £/m^2^), NFκB and P17 form of activated Caspase 3 (C3ap17) activations, and apoptosis are observed in a limited qualitative manner in the original model in Boolean Network format, while this correlation is observed quantitatively in the initial and extended apoptosis models in PBN format. Note that the model fitting of NFκB in the case of Fas ligand activation, ‘FasL (2)’, in the extended apoptosis model is improved as a result from the additional molecular interaction derived from literature.

In addition, the inverse correlation between the intensity of UVB irradiation, the activations of NFκB and Caspase 3, and the apoptotic activity were also identified in a quantitative manner. Namely, a stronger UVB irradiation (i.e., UV2 = 600 £/m^2^>UV1 = 300 £/m^2^) resulted in a stronger NFκB pathway activation (0.3083 against 0.0023) but a weaker Caspase 3 activation (0.0016 against 0.9966) and a weaker apoptotic activity (0.4681 against 0.9920). In contrast, the original study of Schlatter *et al.* could only identify this relationship in a limited qualitative manner [4].

#### optPBN allows for an estimation of interactions' relevancy in a context-specific manner

As can be seen in Table 5, the fitted ‘initial apoptosis model’ (Init.) failed to explain some of the experimental data. For instance, the fitting of NFκB in a condition with a high concentration of Fas ligand stimulation (FasL (2)) is not in a good agreement with the experimental measurement (0.0198 against 0.32). This observation raises a question whether the set of considered molecular interactions was sufficient to model the context-specific apoptotic signalling in the hepatocytes.

In fact, NFκB can also be activated through Caspase 8 with a mechanism distinct from that of tumour necrosis factor alpha (TNFα) for cytokine production as described in [32], [33]. Therefore, we modified the Boolean rules to take this information into account and thereby derived a new model called ‘extended apoptosis model’ (see detail in File S2). The results from the optimisation on the ‘extended apoptosis model’ (Ext.) are shown in Table 5. We found that the ‘extended apoptosis model’ fits the experimental data better (cost of 0.199) than the ‘initial apoptosis model’ (cost of 0.328) and the inverse correlation between UVB intensity, NFκB and Caspase 3 activations, and apoptotic activity is still preserved. In addition, we also found that the discrepancy between the simulated model state and the measurement data of NFκB in the FasL (2) experiment is decreased after the addition of the new molecular interaction derived from literature.

In Figure 4, we presented the best 500 values for selection probabilities 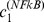 and 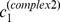 in term of fitting cost. By taking 1-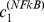 and 1-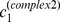, we could determine the values for the selection probabilities 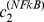 and 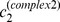 respectively. The mean and the SD values for these selection probabilities are given in Table 6. These statistics were confirmed in 3 independent optimisation runs with 500 best parameter sets considered in each run (see File S2).

**Table 6:** The distributions of fitted selection probabilities on the extended apoptosis model.

Molecule	Functions	Mean	SD
NFkB	= (∼I_kBa|∼I_kBe)	0.8354	0.0209
	= (∼I_kBa|∼I_kBe)|(C8a|C8a_2)	0.1646	0.0209
complex2	= complex1 & FADD	0.8480	0.1105
	= complex1 & FADD & RIP_deubi	0.1520	0.1105

The Boolean rules of two target molecules in the ‘extended apoptosis model’ and the parameter distributions of the top 500 fitted selection probabilities in terms of optimal cost are shown. A low standard deviation (SD) value comparing to the corresponding mean value indicates that model is sensitive to the parameter of the respective Boolean interaction. This information highlights the relevancy of the respective interaction within the context of study e.g., the case of nuclear factor kappa-B (NκFB) activation by Caspase 8 (C8a or C8a_2) together with the absence of nuclear factor of kappa light polypeptide gene enhancer in B-cells inhibitor alpha and epsilon (IκBa and IκBe) inhibitions. In contrast, a high value of standard deviation comparing to the mean value as seen in the case of TNF receptor-1 signalling complex 2 (complex2) activation by deubiquitinated form of receptor associated receptor kinase 1 (RIP_deubi) suggests that the model might not be sensitive to the respective parameter in this study. The relevancy of this interaction within the context of apoptotic signalling in primary hepatocytes is therefore in question.

We found that the selection probability 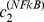 for the Boolean rule which represents the co-influence of Caspase 8 and nuclear factor of kappa light polypeptide gene enhancer in B-cells inhibitor alpha and epsilon (IκBα/IκBε) degradations on the activation of NFκB obtained a mean value of 16% with SD of 2%. The non-zero mean value indicates that this interaction is important to explain the experimental data in the context of our study. Also, the narrow distribution of the values of selection probability for this Boolean rule suggests that the model is sensitive to this parameter. Such information therefore highlights the relevancy of this newly introduced interaction. In contrast, the distribution of the values of the selection probability 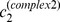 which describes the influence of deubiquitinated form of receptor associated kinase 1 (RIPdeubi) on the activation of TNF receptor-1 signalling complex 2 (complex2) is more spread. This might suggest that the model is less sensitive to this parameter. Therefore, the relevancy of this interaction in the context of our study is still in question.

We tried to further investigate the influence of RIPdeubi on complex2 by following the methodology as presented in [8]. However, we observed that, the required length of the trajectory to estimate the joint distribution on the parent nodes of complex2 is very long and therefore it is practically infeasible to perform this analysis in a reasonable amount of time with the current implementation of the *optPBN* toolbox. Thus, we further estimate the relevancy of this interaction by considering the model topology within the context of our study instead.

Considering the model topology as presented in [4], the Boolean interaction in question represents positive signals on the activation of complex2 by RIPdeubi via two sources (see Figure 8): 1) A positive feedback loop from activated Caspase 8 (C8*) + unknown proteins in type 2 apoptosis (P)→truncated Bid (tBid)→Bax→Smac→RIPdeubi→complex2→C8*-complex2→C8*, and 2) The positive signal from UVB radiation (UV)→Bax→smac→RIPdeubi→complex2. According to the original study, the activation of type 2 receptor (T2R) was only considered and experimentally validated in the context of Jurkat cells, but not hepatocytes [4]. Therefore, the node P which is activated by T2R will never be activated and the feedback loop as shown in 1) is not active in our PBN model (which only fits to the hepatocyte data). In parallel, the activation of complex2 by UVB (via RIPdeubi) requires the presence of TNF receptor-1 signalling complex 1 (complex1). This means the positive interaction in 2) will only be valid when TNF and UVB co-stimulate. Nevertheless, this condition was not validated in the original study [4] nor was constrained in the PBN model either. Thus, the interactions on complex2 activation from 1) and 2) via RIPdeubi as described are not relevant in the context of primary hepatocyte. These interactions could therefore be removed from the PBN model in our study.

We demonstrated that *optPBN* can be applied to determine the selection probabilities in PBN which can subsequently be used to estimate the relevancy of molecular interactions in the context of study. Such information in turn leads to the generation of computational models which could represent the dynamics of biological networks in a context-specific manner.

## Conclusions

We present *optPBN*, a novel optimisation toolbox which provides a simple yet comprehensive pipeline for the generation of integrated optimisation problems in the PBN formalism which can readily be solved by various optimisers on local or grid computational platforms. The *optPBN* toolbox offers two modes of optimisation, discrete and continuous, for the selection of appropriate constituent Boolean networks for the PBN from the pool of available choices and for the determination of selection probabilities from experimental data, respectively. The two modes can be applied for the optimisation and/or inference of the networks in both qualitative and quantitative manner.

*optPBN* was tested and compared against existing optimisation tools for the Boolean formalisms where *optPBN* delivers similar results and it also offers quantitative optimisation. The updated version of *CellNOpt*, *CellNOptR*, is also capable of handling quantitative optimisation, but the respective Boolean models have to be converted into fuzzy logic or ordinary differential equation (ODE) modelling frameworks beforehand [17], [34]. We also demonstrated that *optPBN* allows for the optimisation of an apoptotic network, leading to the generation of an optimised PBN model with the corresponding selection probabilities that fit experimental data. Such results do not only yield a better quantitative insight into biological networks, they can also be further used to evaluate interactions' relevancy within the network in a context-specific manner. Lastly, the computational time which is a major limitation when dealing with complex optimisation problems can be better handled by applying the grid-based implementation of the *optPBN* optimisation pipeline.

### Limitations

Even though *optPBN* offers many simple-to-use functionalities, there are some limitations that come along with the simplicity of the toolbox. First, our approach always requires prior knowledge on the possible interactions between molecules which are given in the form of potential constituent Boolean networks. There exist approaches using Bayesian network with Boolean variables for reconstructing biological networks which do not have this limitation, see e.g., [35]. Second, the formulation of the rule-based modelling has to be in a specific order when there is a combination between parameter and constant value in the assignment of different Boolean rules. For instance, given an output which can be activated by an input (a parameter is assigned to the rule) while it can also be inhibited by an inhibitor (the constant value ‘0’ is assigned to the rule), the Boolean rule which represents the activation by an input has to come first (see more examples in the help section of the script *rule2PBN* in the *optPBN* toolbox). Lastly, *optPBN* uses only one global data structure (estim) to store and process all information of the network so that the integrated optimisation problem can be solved simultaneously for all experimental cases. This setting might not be applicable to solve the optimisation problem where different parameter values are expected for each experimental case (i.e., the optimisation of local parameters). The optimisation of such local parameters is not yet available in the current version of the toolbox.

### Outlook

First, we aim to improve *optPBN* to be capable of optimising local parameters. In addition, we plan to introduce the concept of penalisation for increasing model size (È_s_ and α) as implemented in *CellNOpt* and *CellNOptR* as a part of our objective function in order to generate better results. Second, we foresee that other global optimisation techniques, e.g., Simulated Annealing, Pattern Search methods, or Mode Hopping Metropolis sampling could be integrated into the *optPBN* pipeline. Third, the ambiguity of Boolean rules formulation to properly represent biochemical reactions is yet to be addressed. Lastly, we envisage many useful applications from implementing the *optPBN* toolbox to study biological systems such as the inference of gene regulatory networks from microarray data and the identification of crosstalk signalling's relevancy in mammalian signal transduction networks based on experimental data in a context-specific manner.

### Software Availability and Requirements

**Project name:** optPBN
**Project home page:** http://sourceforge.net/projects/optpbn
**Operating system(s):** Platform independent
**Programming language**: Matlab (and C++ for grid-based version)
**Other requirements**: BN/PBN Toolbox, Systems Biology Toolbox 2 (with Message Passing Interface (MPI) and ParadisEO for grid-based version)
**License**: GNU GPL v3.0 (with CeCILL license required for ParadisEO framework)
**Restrictions**: no restrictions except for commercial use

## 

## Supporting Information

Figure S1 **Compared results from** ***optPBN*** **and** ***CellNOpt*** **on the original toy model of Saez-Rodriguez** ***et al.*** [A] The model structure of the original toy model of Saez-Rodriguez *et al.* [18] is shown on the left panel. The experimental descriptions and the corresponding artificial measurement data are shown in the left and right tables respectively. [B] The results from *CellNOpt* under a defined set of size penalty (0≤α<0.23 and È_s_ = 0.58) identifies the AND (&) gate for the connection between PI3K and TNFa to NFkB. The interaction from Raf was identified as the only factor that activates ERK. [C] The results from the optimisation with *optPBN* toolbox in discrete mode are in a good agreement with *CellNOpt* for NFkB. Furthermore, *optPBN* also discovered the OR (|) gate for the connection between Raf and NFkB to ERK as an additional solution.
(PDF)

File S1 **Stand-alone version of** ***optPBN*** **toolbox.** The compressed zip file contains all scripts of *optPBN* toolbox in stand-alone version. Also, it contains three scripts of *optPBN* toolbox in grid-based version with the tag ‘_G5K.m’ for the analysis of optimisation results obtained from the grid-based pipeline which runs on a local computer. To install the stand-alone version, a wrap-up script in Matlab (install.m) is provided for the ease of installation. The installations of the *BN/PBN* toolbox [20] and the optimisation toolbox of Systems Biology Toolbox 2 (SBtoolbox2) [24], which are required for the running of *optPBN* toolbox, are also included in the wrap-up script. The grid-based version of the *optPBN* toolbox is provided separately and can be downloaded at https://sourceforge.net/projects/optpbn. It comprises all packages needed for running the algorithm on a cluster or on a grid-based infrastructure. This includes the ParadisEO 1.1 framework (evolutionary algorithms and parallelisation support), MPICH2, LibXML2, GSL, MCR and the *optPBN* grid-based implementation in itself. A detailed description on the installation and execution of the grid-based pipeline can be found in File S9.
(ZIP)

File S2 **Computational scripts of all examples and the corresponding original result files.** The compressed zip file contains the *optPBN* optimisation pipeline in the form of Matlab scripts (.m) for all examples used in this study (case study 1, case study 2, toy models of Saez-Rodriguez *et al.*, i.e. case study 3, and Boolean model of apoptosis of Schlatter *et al.*, i.e. case study 4). For the results presented in this article, the integrated optimisation problems of the first 3 case studies were optimised by using the stand-alone version of the *optPBN* pipeline applying particle swarm optimisation as the optimiser on a single local machine. The last case study (Schlatter's model, i.e. case study 4), due to a complex optimisation problem, was optimised by using the grid-based version of the *optPBN* pipeline applying a combined differential evolution and evolutionary algorithms as the optimisers on the Grid'5000. The corresponding results from the optimisations of each model presented in the article are included in a matrix format (.mat and .log) for further analysis on the distributions of optimised parameters. A complete set of result files for four case studies and the results from additional analysis as presented in File S8 can be downloaded at https://sourceforge.net/projects/optpbn.
(ZIP)

File S3 **Manual of the** ***optPBN*** **toolbox.** The PDF manual provides a detailed description of the *optPBN* optimisation pipeline. A step-by-step guideline on how to use the *optPBN* toolbox together with the explanation of the core idea for each computational script is provided in the document.
(PDF)

File S4 **Grid-based pipeline of** ***optPBN*** **toolbox.** The PDF documentation provides a description on the grid-based pipeline of the *optPBN* toolbox. It also describes a strategy to combine two optimisation algorithms, evolutionary algorithm (EA) and differential evolution algorithm (DE), as a single optimiser. An optimisation run on Grid'5000 is demonstrated as an example.
(PDF)

File S5
**Derivations of the two-state Markov chain approach's formulas.** The PDF documentation provides the corrected derivations of the two-state Markov chain approach's formulas in relation to the derivations presented in the original work of Raftery and Lewis [30]. The term *λ* which refers to 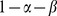 in the calculation of *m*_0_ should be replaced with 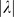 (i.e., 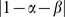). In addition, Φ which denotes the standard normal cumulative distribution function in the calculation of *N* should be substituted with the inverse of its function, Φ^−1^, after derivations.(PDF)

File S6 **Results of** ***optPBN*** **for four case studies.** The spread sheet provides a complete set of results generated from the *optPBN* pipeline for the four case studies. Different numbers of (selection probability) parameter samplings from optimisers and a range of accuracy parameter ‘r’ from 0.01 to 0.05 are explored. Computation time and quality of model fitting were reported for both stand-alone and grid-based versions.
(XLSX)

File S7 **Normalised and justified experimental data for the study of apoptosis model from Schlatter** ***et al.*** The spread sheet provides a detailed description of the measurement data that were used for the optimisation of the Boolean and probabilistic Boolean models of apoptosis from Schlatter *et al.* The pipeline on background subtraction and normalisation of experimental data with the justification on the saturation of the signal for p17 form of activated Caspase 3 is described in detail.
(XLS)

File S8 **Analysis of the approximation of steady-state distributions with different initial conditions and accuracies.** The spread sheet presents the steady-state probability of output nodes in case study 4 which are approximated by *optPBN* applying a fixed set of selection probabilities (randomly generated) with different sets of initial conditions. Three sets of initial conditions (random, all zeros, and all ones) together with two levels of accuracies (r = 0.025 and r = 0.01) were explored. Steady-state probabilities of output nodes and parameters of two-state Markov chain approach are shown. The differences of these values across different sets of initial conditions and across two levels of accuracies are also presented.
(XLS)

File S9 **Installation guide for the grid-based pipeline of** ***optPBN*** **and an execution example on Grid'5000.** The PDF documentation provides a list of commands to set-up the grid-based version of the *optPBN* toolbox on a cluster or on a grid-based infrastructure. An example set of commands to reserve resources and to execute an optimisation task on Grid'5000 is also included.
(PDF)
