# Correction: optPBN: An Optimisation Toolbox for Probabilistic Boolean Networks

**DOI:** 10.1371/journal.pone.0105913

**Published:** 2014-08-12

**Authors:** The PLOS ONE Staff

## Notice of Republication

This article was republished on July 24, 2014, to correct Figure S1, which would not open. The publisher apologizes for the error. Please view this article again to download the file.
