# Supplementary material for: optPBN: An Optimisation Toolbox for Probabilistic Boolean Networks
Source: PLoS One. 2014 Jul 1;9(7):e98001. doi: 10.1371/journal.pone.0098001 (PMC4077690; doi:10.1371/journal.pone.0098001)
Supplement: Figure S1 — Compared results from optPBN and CellNOpt on the original toy model of Saez-Rodriguez et al. [A] The model structure of the original toy model of Saez-Rodriguez et al. [18] is shown on the left panel. The experimental descriptions and the corresponding artificial measurement data are shown in the left and right tables respectively. [B] The results from CellNOpt under a defined set of size penalty (0≤α<0.23 and Ès = 0.58) identifies the AND (&) gate for the connection between PI3K and TNFa to NFkB. The interaction from Raf was identified as the only factor that activates ERK. [C] The results from the optimisation with optPBN toolbox in discrete mode are in a good agreement with CellNOpt for NFkB. Furthermore, optPBN also discovered the OR (|) gate for the connection between Raf and NFkB to ERK as an additional solution. (PDF) [file pone.0098001.s001.pdf]

A Model structure of the original Toy model (adapted from Saez-Rodriguez *et al.*, doi: 10.1038/msb.2009.87)

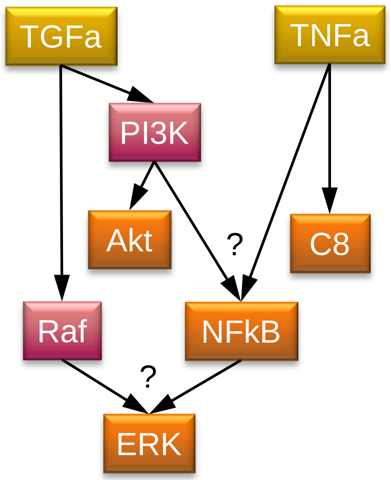

	Input			
Exp	TGFa	TNFa	PI3Ki	Rafi
A	1	0	-	-
B	0	1	-	-
C	1	1	-	-
D	1	0	+	-
E	1	1	+	-
F	1	0	-	+

	Output			
Exp	NFkB	ERK	C8	Akt
A	0	1	0	1
B	0	0	1	0
C	1	1	1	1
D	0	1	0	0
E	0	1	1	0
F	0	0	0	1

B CellNOpt results adapted from the original article of Saez-Rodriguez *et al.* (doi: 10.1038/msb.2009.87)

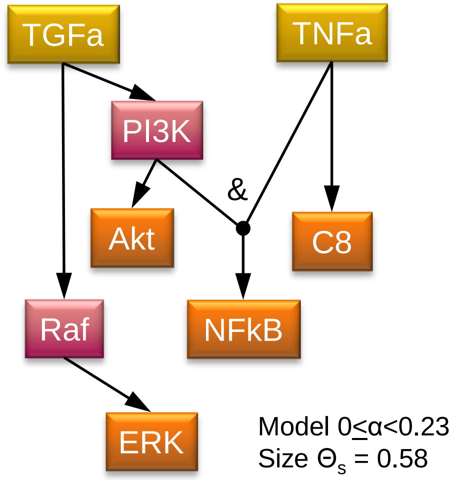

C Result from the optimization with optPBN toolbox in discrete optimization mode; Left: NFkB, Right: ERK

NFkB

Exp	|	&	PI3K	TNFa	Ø
All	×(0)	✓(1)	×(0)	×(0)	×(0)

ERK

Exp	|	&	Raf	NFkB	Ø
All	✓(0.5)	×(0)	✓(0.5)	×(0)	×(0)
