# Supplementary material for: optPBN: An Optimisation Toolbox for Probabilistic Boolean Networks
Source: PLoS One. 2014 Jul 1;9(7):e98001. doi: 10.1371/journal.pone.0098001 (PMC4077690; doi:10.1371/journal.pone.0098001)
Supplement: File S1 — Stand-alone version of optPBN toolbox. The compressed zip file contains all scripts of optPBN toolbox in stand-alone version. Also, it contains three scripts of optPBN toolbox in grid-based version with the tag ‘_G5K.m’ for the analysis of optimisation results obtained from the grid-based pipeline which runs on a local computer. To install the stand-alone version, a wrap-up script in Matlab (install.m) is provided for the ease of installation. The installations of the BN/PBN toolbox [20] and the optimisation toolbox of Systems Biology Toolbox 2 (SBtoolbox2) [24], which are required for the running of optPBN toolbox, are also included in the wrap-up script. The grid-based version of the optPBN toolbox is provided separately and can be downloaded at https://sourceforge.net/projects/optpbn. It comprises all packages needed for running the algorithm on a cluster or on a grid-based infrastructure. This includes the ParadisEO 1.1 framework (evolutionary algorithms and parallelisation support), MPICH2, LibXML2, GSL, MCR and the optPBN grid-based implementation in itself. A detailed description on the installation and execution of the grid-based pipeline can be found in File S9. (ZIP) [file pone.0098001.s002.zip › optPBN_stand_alone_v2.1/SBTOOLBOX2/optimization/auxiliary/EvA2/JE2Doc.pdf]

JavaEvA 2 Short Documentation

Marcel Kronfeld

Dept. of Computer Architecture,

Prof. Dr. Andreas Zell,

University of Tübingen
URL: http://www.ra.cs.uni-tuebingen.de/software/JavaEvA

We like to thank all former and current developers of JavaEvA (Jürgen Wakunda, Holger Ulmer, Felix Streichert, and Hannes Planatscher) and all the students involved in the developement.

Contents

1. Introduction 4
2. Quick Start 5
3. External Interfaces 10
4. Quickly Adding a Problem Class 14
5. Using the JavaEvA 2 API 15
6. Further Reading 23

Bibliography 24

1. Introduction

JavaEvA 2 (a Java implementation of Evolutionary Algorithms, revised version 2) is a comprehensive heuristic optimization framework with emphasis on Evolutionary Algorithms. It is a revised version of the JavaEvA [**7**] optimization toolbox, which has been developed as a resumption of the former EvA software package [**8**].

JavaEvA 2 integrates several derivation free optimization methods, preferably population based, such as Evolution Strategies, Genetic Algorithms, Differential Evolution, Particle Swarm Optimization, as well as classical techniques such as multi-start Hill Climbing or Simulated Annealing.

JavaEvA 2 aims at two groups of users. Firstly, the end user who does not know much about the theory of Evolutionary Algorithms, but wants to use Evolutionary Algorithms to solve an application problem. Secondly, the scientific user who wants to investigate the performance of different optimization algorithms or wants to compare the effect of alternative or specialized evolutionary or heuristic operators. The latter usually knows more about evolutionary algorithms or heuristic optimization and is able to extend JavaEvA 2 by adding specific optimization strategies or solution representations.

This document is, as the title says, not an extensive manual on the JavaEvA 2 framework, but instead a short introduction hoping to ease access to JavaEvA 2. Thus, the document is mainly sketched along use-cases and tries to deliver knowledge on a top-down basis, with most important things first and details where required. Still, as JavaEvA, just as mostly any larger software package, can become tricky sometimes, it is not always possible to explain things without cross-references. We hope that this document will, anyways, be a valuable helper in working with JavaEvA 2.

This document contains, of course, a Quick Start guide (Sec. 2) also explaining the graphical user interface (GUI, Sec. 2.3). Sec. 3 contains hints on how to use JavaEvA 2 with external programs, e.g. MATLAB. We provide a quick-and-simple way to add an application problem implementation in Sec. 4 and describe more details of the API in Sec. 5 to access further options and functionality. Finally, we propose some literature sources for readings on Evolutionary and Heuristic Optimization in Sec. 6 for the interested users.

2. Quick Start

The following sections give a short introduction in the main aspects of using JavaEvA 2, explaining the possibilities accessible by the GUI. Even if you want to use the API without the GUI, we recommend to try some optimization runs through the GUI first, as it will help to learn about the concepts used in the framework.

2.1. Running JavaEvA. To quickly test JavaEvA 2, we recommend you download the jar-package *JE2Base.jar* and start the GUI. The jar-file can be downloaded from the JavaEvA homepage[1] [**1**].

To start under GNU/Linux, you can just type:

```
$ java -cp JE2Base.jar javaeva.client.EvAClient
```

Note that "$" stands for the command prompt, which needs not to be typed. In the same or a similar way, you can start it on all other platforms that support Java. For technical reasons, we discourage using the Java option *-jar* with JavaEvA 2.

If you want to work on the source code directly, note that it is also vital to copy the resource folder to the location where the compiled class files are located. You can then again start the GUI or optimize through the API (Sec. 5). However we advise you to learn to know the GUI a little before digging in the source code.

2.2. Some Words on the Words. As JavaEvA 2 is mainly about Evolutionary and Heuristic Optimization, some of the terms and notions are borrowed from the area and used in this document. As they may not be familiar to all who want to use the framework, we give a short summary here.

We aim at optimizing a target function without knowing much about it, and find a certain position in the search space which minimizes the function, called the *solution*. During search, we use a specific search strategy, the *optimizer*, which usually looks at several positions in parallel. Those are all *potential solutions*, because we don't know the real one yet. For the potential solutions we evaluate the target function. The value received is often called *fitness* in analogy to Darwin's Theory of Evolution, where "the fitter ones survive". For the same reason, potential solutions are sometimes called *individuals*, and the set of potential solutions stored by the optimizer at a time may be called *the population*. Many of the implemented optimization strategies employ operators in analogy to natural *mutation*, *crossover* and *selection*.

There is nothing mystical about that, and of course the analogy is often exaggerated. Evolutionary Optimization is an algorithmic tool that serves mostly technical purposes. That it works in a computer is by no means a sign that we fully understand natural evolution or can proof anything about it. This said, of course, we would never doubt that natural evolution in fact works.

This document will not explain in detail how the implemented optimizers work, as there is enough literature out there handling these topics. We refer to Sec. 6 for suggestions on further reading.

[1] http://www.ra.cs.uni-tuebingen.de/software/JavaEvA/

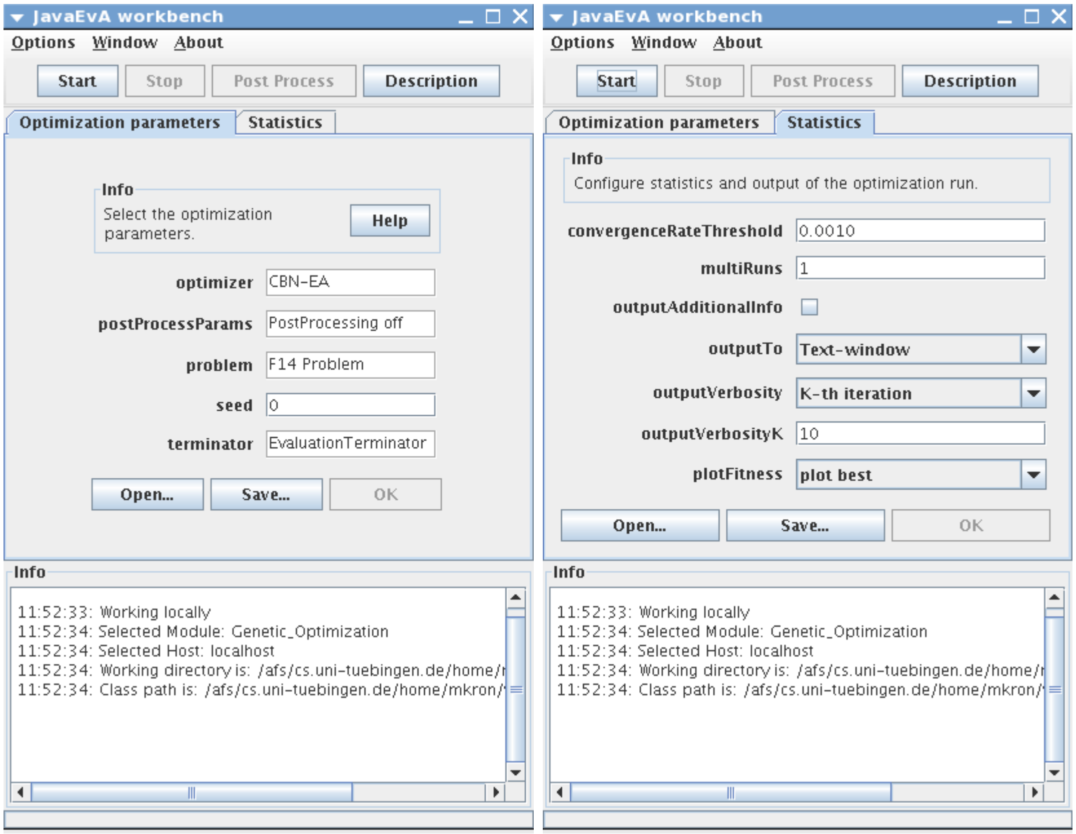

FIGURE 1. Screenshots of the workbench window, with optimization (left) and statistics (right) parameters.

2.3. Using the GUI. From the GUI, also called the JAVAEVA workbench, all important components of an optimization run can be accessed and configured. To change the optimization method, for example, click on the field labeled with "*optimizer*" and select the desired algorithm from the drop-down menu. Basically, you thereby select a Java class and create an instance, whose public properties are displayed in the window immediately with their standard values. For your optimization run, you may configure the parameter values directly through the input fields. A short description will be displayed by tip-text above the name of each parameter. The JAVAEVA GUI has two main tabs: the optimization parameter tab and the statistics tab (Fig. 1), the components of which will be summarized in the following.

2.3.1. *The Workbench Window.* The optimization parameters:

***Optimizer*:** Select the main optimization method. You can choose between classical as well as evolutionary and swarm-based optimization methods. For quick optimization, just use the standard values of the parameters and try several different optimizers.

***Post-processing parameters*:** In some cases, post processing of the results is desirable, e.g. if you want to improve the single found solution by small hill climbing steps, or if you want to retrieve more than one solution from a clustering optimization approach.

***Problem*:** The instance of the target function to be optimized is specified here. You can select from the benchmark problems delivered with the package or inherit from the problem class yourself (Sec. 5).

***Random Seed*:** As most algorithms in JavaEvA 2 incorporate stochastic components, the random seed is critical for the specific outcome of a run. For replicable results, set a seed value > 0. To receive statistically relevant results, test several times with a seed of 0, which means that the system time is used as seed for each new run, or use the multi-run option.

***Termination Criterion*:** Set the criterion by which to stop an optimization run, e.g. stop after n fitness evaluations.

The Statistics parameters:

***Convergence Rate Threshold*:** Provided the target value is zero, convergence is assumed if a value smaller than this threshold is reached. For multi-run experiments, the number of hits is counted using this criterion.

***Number of Multi-runs*:** To achieve statistically meaningful results on how well a certain optimizer works on a given problem, set this number to do several runs in a row. The plot will be averaged, while all intermediate data can be collected in an output file or text window.

Additional Information Output: In addition to typical data output such as best and average fitness at a time, some problems may be requested to display additional information in textual form, usually a representation of the best individual.

***Output To*:** Textual information can be shown in a text box, or redirected to a file, or both. The output file will be stored to the current directory with a descriptive name containing the timestamp of the start of the run.

Output Verbosity: Select the verbosity of the textual output, possible settings are "no output", "final results", "k-th iterations" and "all iterations". An iteration is usually a generational cycle, meaning that for "all iterations", intermediate data is printed after each generation.

Verbosity Parameter k: Define the interval parameter for the "k-th iterations" setting of the output verbosity, by which intermediate data is printed.

***Fitness to Plot*:** Define which fitness values to be displayed in the plot window after every generation. Select "best", "worst" or both.

Finally, there are three buttons on top. *"Description"* shows some very general information on the main JavaEvA module. The "*Start Optimization*" button, as expected, starts an optimization run using the parameters set, or multiple runs sequentially if *multiRuns* is set higher than one. During optimization, the "*Stop*" button can abort the (multi-)run.

2.3.2. *The Plot Window.* During the optimization run, the progress of the solution is plottet to a graph in a separate window (Fig. 2). Usually, the fitness of the best individual of every generation is drawn in Y-direction along the number of function calls in X-direction. Be aware that for multi-objective problems, only the first fitness dimension is shown. The 2-dimensional multi-objective problem classes have, however, a pareto-front viewer which displays the population in the two fitness dimensions sequentially. For higher fitness dimensions it is more practical to use external tools for visualization. Figure 2, by the way, shows the

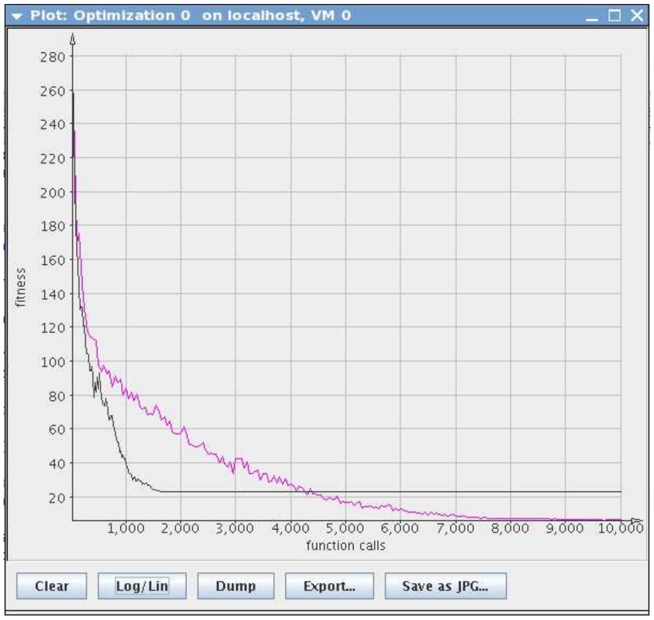

FIGURE 2. Plot window comparing a simple (5,20)-ES and PSO on Rastrigin's after 10 runs each.

fitness progress averaged over 10 runs of a (5,20)-ES and a PSO strategy on Rastrigin's Problem: PSO converges slower, but finds better results on the long run, while ES settles earlier on higher plateaus.

The visible buttons have the following functions:

***Clear*:** Remove all graphs from the plot window.
***Log/Lin*:** Switch between linear and log-scaled view. Most benchmark problems in JavaEvA are implemented with the minimum fitness at zero, so that the log-scale view allows to compare and analyze convergence behaviour in detail. Of course, if the target fitness may become zero or negative, log-scale view is impossible.
***Dump*:** Export the contained data to standard output. For each graph, a column is created in the same order they were generated.
***Export*:** Create the same output as *Dump* and save it to a file.
***Save as JPG*:** Create a JPEG image of the plot window and save it to a file.

2.3.3. *Basic Optimization using* JavaEvA 2. To get a grip on JavaEvA 2 and what optimization means, it is best to run some experiments on the implemented standard benchmarks. To do that, start the GUI and select a benchmark problem, e.g. the F1-Problem consisting in a simple hyper-parabola. Leave the post-processing deactivated. Then choose an optimizer, such as Evolution Strategies with standard parameters, set the termination criterion to EvaluationTerminator with 10,000 fitness calls and push the "Start Optimization" button at the top of the window. Two additional windows will now open up: the plot window with a fitness graph, and a text box displaying the optimization progess in textual form. The final result will be printed into the text box at the end of the run, as well.

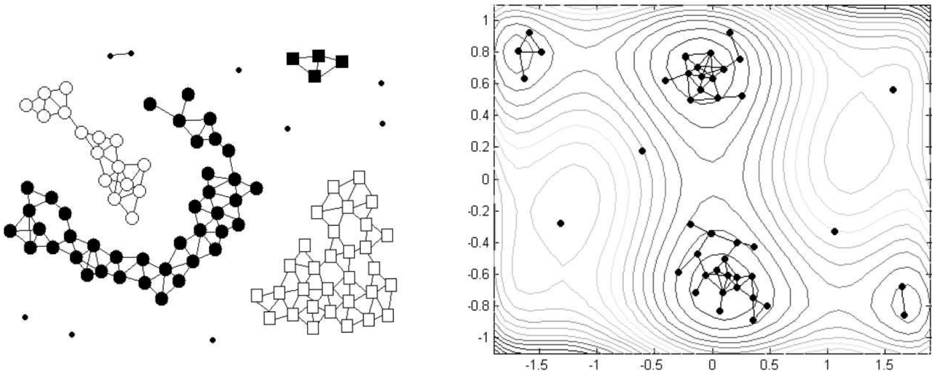

FIGURE 3. Examples for density based clustering (Streichert et al. [**6**]).

If you play around with some optimizer settings, e.g. you try different values for μ and λ or activate the plusStrategy checkbox, you will notice changing performance of the ES. On problems with in discrete space, such as the B1-benchmark problem, for example, a Genetic Algorithm is often superior to an Evolution Strategy. You can try this if you clear the plot window, select the B1-problem and run the ES a few times. Now, switch to the Genetic Algorithm and run the optimization a few more times.

Notice, however, that by changing from the F1-problem to the B1-problem, the internal representation of individuals may change. As B1 is a typical binary problem, it uses GAIndividuals by default, which are based on binary vectors, while F1 uses double vectors. Be aware, that not all optimizers in JAVAEVA 2 are built to work on all types of indviduals.

2.3.4. *Post-Processing.* To see how post processing works, you can select the *FM0Problem* from the problem list, which is a simple target function with a global and a local optimum. Select the *ClusterBasedNiching* algorithm as the optimizer. Now click on the *postProcessingParams* and activate them. For a clustering distance of $\sigma = 0.1$ and $\approx 5,000$ hill climbing steps, the optimizer should print out just a few solutions in the text box, the first of which hopefully are the optima near (1.7/0) and (-1.44/0).

Post-processing serves mainly two purposes: filter redundant solutions and refine the search results. Redundant solutions occur naturally in population-based heuristics. The optimizer handles several potential solutions in parallel, and it is hoped that they all converge on the global optimum during the run. Or for multi-modal problems which have several local optima, it can be desirable to have parts of the population converge in different areas of the solution space. In any case, one usually wants to retrieve *the* solution set or a refined global optimum. For this purpose, we employ a clustering approach which takes the whole solution set and merges similar solutions to an associated subset. For each of these bulks, only the best individual is returned in the filtered solution set.

Of course the size of this filtered set depends on the degree of convergence in the original set and on the clustering criterion. We employ density based clustering [**4**], which associates any two individuals which have a distance of less than the clustering parameter σ (Fig. 3). This is an intuitive approach that does not require a predefined number of clusters, in contrast to k-means, for example. By defining σ, you thus define the resolution you grant your solution set.

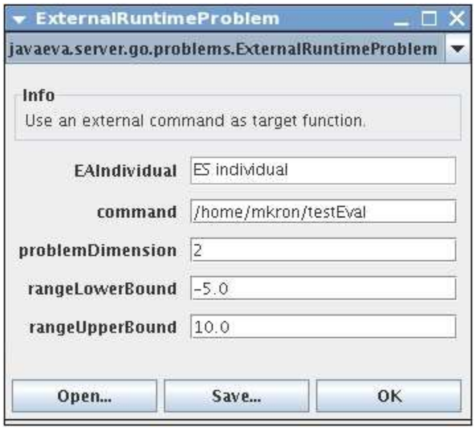

FIGURE 4. Screenshot of an external runtime problem configuration.

As the solution set always contains the last state of the heuristic optimization, one may hope that it is converged. But of course often it is not fully converged, or maybe the strategy even rediversifies the population from time to time, meaning that some part of the set it is converged while other individuals are freshly initialized and thus by no means optimal. So after filtering out redundancy, you might also want to refine the returned set a little. This can be done directly by Hill Climbing (HC) in the post-processing step by setting *postProcessSteps* to the number of evaluation calls you want to invest in the refinement.

If you set σ for clustering and performed hill climbing, then there will be another clustering step right after the HC process, to remove redundancy that emerged by the additional HC optimization.

2.4. Additional Packages. To add additional packages to use them with the JAVAEVA 2 base package, you can just add them to the class path. For instance, to use the additional JE2Probs package containing a larger set of benchmark problems, place them both in your working directory and type (GNU/Linux):

```
$ java -cp JE2Base.jar:JE2Probs.jar javaeva.client.EvAClient
```

You should now be able to select from a larger set of optimization problems in the GUI. Note that different platforms use different characters as path separators (':' in GNU/Linux). To add your own classes to the JAVAEVA 2 framework (see Sec. 4), you need to add your local development path to the classpath, for example:

```
$ java -cp JE2Base.jar:JE2Probs.jar:/home/username/OwnClassDir \
    javaeva.client.EvAClient
```

Note that JAVAEVA 2 will search all classpath entries for compatible classes, so you should only add those packages which are really required.

3. External Interfaces

3.1. Optimizing Through an External Command. For easy optimization using a runnable program, we provide the `ExternalRuntimeProblem` class. So if you have a runnable

function implementation called "*testEval*" (or "*testEval.exe*"), that takes a series of double valued arguments and produces a result value as output (on *stdout*), you can select `ExternalRuntimeProblem` in the GUI, enter the path and program name into the command field, adapt boundaries and the problem dimension and just start optimization. For a programm called *testEval* with a two-dimensional solution space, which you want to optimize within $[-5, 10]^2$, the configuration can be seen in Fig. 4. In the example, *testEval* may be called and, in the single objective case, should produce output as in:

```
$ /home/username/testEval 7.1642923 -4.2346211
28.1371802
$ _
```

JavaEvA 2 starts a system process to run the external program and converts input/output using String objects, which is by itself rather slow. Still, it may outweigh the costs of reimplementing it anew and can even be negligible if the runtime of the calculation is of higher order of magnitude. It may also just come in handy if you want to try what JavaEvA 2 might do for you.

3.2. Optimization from Matlab.

3.2.1. *Quick Howto.* To tackle optimization problems already existing in Matlab, we provide a simple Matlab interface comprising in a Matlab class definition. If you have an existing Matlab function you want to optimize with some standard algorithms implemented in JavaEvA 2, you can do this now directly from your Matlab console. To do so, follow these steps:

(1) Download the JE2Base.jar and add it to the Matlab classpath, e.g. by typing `javaaddpath /home/username/JE2Base.jar` in the Matlab console.
(2) Download the Matlab JEInterface code and extract it to your Matlab working directory within its own class directory "@JEInterface".
(3) Define the range of your search space using a $2 \times d$ matrix consisting of the lower and upper bounds of the allowed space, e.g. enter `R=[[-5 -5 -5];[5 5 5]]` to define a 3-dimensional search space with bounds -5/5 in every dimension.
(4) For a target function *fun* to be minimized, create a JEInterface in Matlab by typing, for example: `JI=JEInterface('JI', @fun, R)`. Notice that *fun* must be accessible from your working directory, it should not be placed in the @JEInterface directory.
(5) To view the possible optimization strategies, type `showOptimizers(JI)`.
(6) You can now select an optimizer and use its ID to start the optimization, e.g. `JI=optimize(JI,1)` for a standard ES.
(7) Wait for the optimization to finish and type `getResult(JI)` to get the best solution found.

3.2.2. *Details on JEInterface.* The main `optimize` command starts actually two new Java threads handling Java callbacks to Matlab within the single Matlab thread. The result of the optimization will be written back to the JEInterface instance and is not a direct output value of the `optimize` call. There will be a text notice in the Matlab console as soon as the optimization has finished. Notice that in Matlab object oriented style, an object `Obj` cannot be modified simply by calling the mutator, e.g. `setParameter(Obj, p1, val1)`, but must be reassigned for every mutating call, as in: `Obj=setParameter(Obj, p1, val1)`. After optimization, you

may retrieve the result by calling `getResult` on the interface instance. Or, in case you want to retrieve multiple solutions to the target function, you may start a post processing step and retrieve a list of solutions using `getMultipleSolutions`.

Some optimizers allow the solutions to get worse during the optimization run. This allows them to overcome local optima and increases the chance to find a global one. But on the other hand it means that the result of the last optimization iteration is not necessarily the best solution found during the whole run. Therefore, JavaEvA 2 saves the best solution found external to the optimizer, which is what is returned by the `getResult` method.

Most optimization strategies have specific parameters influencing their performance. The `optimize`-call gives access to standard parameter settings. To modify specific parameters, use the `getDesc`-method to list available parameters of an optimizer. You may then use the method `optimizeWith` and deliver specific parameters in name/value pairs to configure a specific run, where the names must correspond to a member variable of the optimizer as listed by `getDesc` and the value object must of course be of the correct type.

For further details, check the method overview in the list below. Each interface method (except the constructor) has at least a parameter `JI`, designating the JEInterface object to work on.

`JI=JEInterface(interfaceName,fhandle,range[,optset,fargs])`: JEInterface constructor: *interfaceName*: the name bound to the JEInterface in Matlab as char. *fhandle*: handle of the target function. *range*: a 2×dim array defining the solution subspace with lower and upper bounds. *optset*: an (optional) *optimset* structure of which *MaxFunCalls*, *TolX* and *TolFun* are used as termination criteria, while *Display* may trigger verbose output. *fargs*: (optional) additional static parameters to the target function as single list. Returns the new JEInterface instance.

`showOptimizers(JI)`: Print a list of optimization strategies accessible through JEInterface by ID numbers.

`JI=optimize(JI,optType[,resultFilePrefix])`: Start the optimization using the strategy indicated by the ID *optType*. Optionally write verbose results to an output file with prefix *resultFilePrefix* in the working directory. Returns the modified JEInterface instance. The method also opens a message box with a cancel button to stop the optimization.

`[sol,fit]=getResult(JI)`: After optimization: return the final solution and its fitness. During optimization: return the current best solution and its fitness.

`getMessage(JI)`: Return a text message describing the termination state of the optimization, e.g. the number of function calls performed.

`JI=postProcess(JI,steps,sigmaClust[,nBest])`: Do post-processing of the last optimization results and export the solution set to the interface instance (cf. Sec. 2.3.4). If *sigmaClust* > 0 a clustering step is performed (density based clustering), so that any solutions in a distance below *sigmaClust* are associated. Notice that *sigmaClust* is treated relative to the problem range. If *steps* > 0, then a hill climbing step is performed after clustering for the given number of evaluations. If *nBest* is given, it defines the maximal number of solutions to be exported to the interface instance. Post processing can be stopped by `JI=stopOptimize(JI)` just as the optimization itself.

`[sols,fits]=getMultipleResults(JI)`: If a post processing step has been performed: retrieve the solution set in a matrix and calculate the corresponding fitness values.

`getOptions(JI)`: Get the current *optimset* structure of the JEInterface instance.

`getOpt(JI,optName)`: Get the value of a specific option *optName* from the *optimset* structure of the JEInterface instance.

`JI=setOpt(JI,optName,optVal)`: Set a specific option *optName* of the *optimset* structure in `JI` to the value *optVal*. Returns the modified JEInterface instance.

`JI=setOptions(JI,optset)`: Set a whole new *optimset* structure *optset*. Returns the modified JEInterface instance.

`getDesc(JI,optType)`: Show information about an optimizer class together with the parameters that can be modified. The available settings are analogous to the optimizer options presented by the Java GUI.

`optimizeWith(JI,optType[,outputFilePrefix][,memName,memVal]*)`: Optimize using an optimizer by ID and specific settings for this optimizer. The settings given must conform to the options shown by `getDesc(JI,optType)`. The available settings are analogous to the optimizer options presented by the Java GUI. The method also opens a message box with a cancel button to stop the optimization.

`JI=stopOptimize(JI[,'kill'])`: Stop a running optimization process, which is usually done by the cancel button. In case brute force was used (STRG-C), this method may be used with the `'kill'` argument to try to clean up Java threads still running as "zombies".

`setResultJE`: (Internal) Write-back method required internally by JavaEvA 2. Calling it by hand may interfere with optimization.

`setResultArrayJE`: (Internal) Write-back method required internally by JavaEvA 2. Calling it by hand may interfere with optimization.

`getProgress(JI)`: (Internal) During optimization, return the number of fitness calls performed until now.

`runEvalLoopJE`: (Internal) Start a JavaEvA run, optimization or post processing.

The methods are listed in about the order a user might require them in. The constructor as well as all mutating methods return a JEInterface object which is to be assigned to a variable, preferably the same as the one assigned on construction. This is a drawback of the Matlab object concept: mutators are actually also constructors and copy the whole Matlab object, but they do not copy the referenced Java instance.

Notice that the last three methods, `setResultJE`, `setResultArrayJE` and `evaluateJE`, are used internally by the Java part of the interface and should not be called by hand. To find out more about the optimization strategies you can access through JEInterface, use the `showOptimizers` method and take a look at the EA references, e.g. the technical report on JavaEvA [**7**].

3.2.3. *Optimization Options.* Vital for the usage in Matlab are the termination criteria of the optimization run. We adopt the builtin *optimset* structure in analogy to the Matlab function *fminsearch,* using the three parameters described in the list below. To find out more about *optimset*, check the Matlab documentation.

***MaxFunEvals*:** Maximum number of function evaluations allowed.

***TolX*:** Convergence tolerance in solution space, measured absolutely. This means that the run is seen as converged, if the best individual (the best parameter set) does not change more than the given threshold for a certain time.

***TolFun*:** Convergence tolerance in fitness space, measured absolutely. This means that the run is seen as converged, if the best fitness found does not change more than this threshold for a certain time.

Display: Triggers online output during an optimization process on the Matlab console. Allowed values are '`off`'/'`final`'/'`notify`' and '`iter`'. Due to the optimset restrictions, '`notify`' corresponds to "show every k-th iteration" while '`iter`' corresponds to "show all iterations" compared to the GUI options. The default is '`off`'.

If no *optimset* struct is given to the constructor, the default values for *TolX* and *TolFun* are the same as in Matlab (10^{-4}), while the default value for *MaxFunEvals* is usually 10^4. You can check the options by calling *getOptions* for a JEInterface instance. In analogy to *fminsearch*, the three criteria will be logically combined as in ($MaxFunEvals$ OR ($TolX$ AND $TolFun$)), meaning that reaching *MaxFunEvals* is a hard stopping criterium, while *TolX*/*TolFun* must be both fulfilled to stop the run, if *MaxFunEvals* has not been reached. To change options on an existing object `JI`, call for example `JI=setOpt(JI, 'TolFun', 1e-7)` to set the TolFun convergence threshold to 10^{-7}. If you don't want to regard convergence and just have the optimization perform a certain number of evaluations, set *TolX* and *TolFun* to zero, e.g. by typing `JI=setOptions(JI, optimset(getOptions(JI), 'TolX', 0, 'TolFun', 0))`. Be aware that at least one termination criterion must be defined through the options, or the optimization will not start.

4. Quickly Adding a Problem Class

It is easy to integrate your own Java-coded problem in JavaEvA 2 and optimize it using the GUI. Preconditions for the quick way are only the two following ones:

- A potential solution to your problem can be coded as a double vector or a BitSet.
- You can implement the target function such that it produces a double valued fitness vector from the double vector or BitSet.

If these simple conditions are met, you have the advantage of being able to use the full "Java-Power" to implement a target function and optimize it in JavaEvA 2. Just follow these steps:

- Create an empty class (let's say, `ExampleProblem`) and assign it to the package `simpleprobs`. Put it in a directory called "simpleprobs" within your working directory.
- Have the class inherit from `simpleprobs.SimpleDoubleProblem` or, depending on which datatype you want to use, from `simpleprobs.SimpleBinaryProblem`. Both base types can be used directly from the JavaEvA 2 jar-file (which of course needs to be on the java classpath).
- Implement the method `public int getProblemDimension() {...}` whithin your ExampleProblem, which returns the number of dimensions of the solution space, i.e. the length of the x vector or size of the `BitSet`, respectively. The problem dimension may be a variable defined in your class, but it must not change during an optimization run.
- Implement the method `public double[] eval(double[] x)` for double coded problems or `public double[] eval(BitSet bs)` for binary coded problems, whithin your ExampleProblem, where the fitness of a potential solution is calculated.

- Start the JavaEvA 2 GUI. *Make sure that your working directory is in the Java classpath*! From the problem list, select the *SimpleProblemWrapper* class as optimization problem. The wrapper class allows you to select your ExampleProblem as target function. If you implemented a double valued problem, you may also set a default range parameter, defining the positive and negative bound of the solution space allowed. Now select your prefered optimization method and start the optimization.

5. Using the JavaEvA 2 API

This chapter describes how to incorporate JavaEvA 2 into existing projects through the Java API.

5.1. Accessing Standard Optimizers. A standard optimizer can be defined as a more or less popular optimization algorithm with operators and parameters predefined in a general way so that a user may use it out-of-the-box and expect good optimization results in general. Of course, every expert may have an own notion on what parameters are preferable in general. Therefore we give two usage examples using the API. In any case, a necessary step is to extend a JavaEvA 2 base class constituting the target function. You may, again, use the simpleprobs package described in 4. However, this makes it necessary to use the wrapper class `SimpleProblemWrapper`, which is great for direct GUI usage but may make programming a bit more complicated. We therefore recommend here to go one step higher in hierarchy and extend `AbstractProblemDouble` or `AbstractProblemBinary`.

5.1.1. *The Abstract Problem Classes.* The problem class subtree encapsulates properties of target functions that can be directly optimized by the JavaEvA 2 framework and starts at the `AbstractOptimizationProblem` class. The basic properties of the problem class are functional:

(1) The problem is itself initialized,
(2) the problem knows how to initialize a set of potential solutions, and
(3) the problem evaluates a set of potential solutions.

To implement your own target function in JavaEvA 2, we recommend inheriting from `AbstractProblemDouble` or `AbstractProblemBinary`, depending on your preferred data preppresentation. Integer and program-data-based problems are currently not covered by this document.

The class `AbstractProblemDouble`. The `AbstractProblemDouble` class in the package `javaeva.server.go.problems` encapsulates methods useful to implement double valued target functions. Let's assume you want to implement a function $f_{\text{target}}(x) = y$ with $f_{\text{target}} : \mathbb{R}^n \to \mathbb{R}^m$. Important for double-valued problems is the range of the solution space, defining in any dimension the minimum and maximum value allowed. By setting the `defaultRange` member variable (method `setDefaultRange`) in your constructor, you can easily define a symmetric range $[-dR,\ dR]^n$. If you need a different range definition, you may overload the methods `getRangeLowerBound(int dim)` and `getRangeUpperBound(int dim)` which are to return the upper or lower range limit for a given dimension.

The `AbstractProblemDouble` class also contains a noise parameter and an individual template. The *noise* can be used to add gaussian fluctuation to any target function. If $noise > 0$, it defines the standard deviation of the random disturbance of $f_{\text{target}}(x)$. The individual template

defines the data representation for potential solutions. It also contains evolutionary operators which work directly on the representation, see Sec. 5.3.2 for a usage example.

We will now give short comments on further important member functions:

`public Object clone():` Object copy operator. We recommend implementing a copy constructor `public YourProblem(YourProblem o)` and refering to it from within the `clone()` method. You may call `cloneObjects` from AbstractProblemDouble to copy the super members. Make sure that the copy constructor copies all necessary member variables you added to your class.

`public void evaluate(AbstractEAIndividual individual):` Main evaluation method. We recommend not to override it.

`public abstract double[] eval(double[] x):` Essential evaluation method. Override it to implement the desired target function $f_{target}(x)$. Make sure that the delivered solution vector is always of the same dimensionality m. Even if your problem is one-dimensional, return an array of length 1 with the single fitness value as the only field.

`public abstract int getProblemDimension():` Return the problem dimension in solution space. Make sure it is constantly equal to n during an optimization run. If you implement a corresponding method `public void setProblemDimension(int n)` and define your class within the same package, you may change the dimension through the GUI.

`public void initProblem():` Called before optimization in general. If you define member variables of your class which are not constant, we recommend setting them to initial values in this function. If you override, make sure you call the super-method from within your version.

`public void initPopulation(Population population):` This method is called before optimization with a certain population. It initializes the population to the problem specific range using the individual template and calls a method of the `Population` collection to initialize the individuals randomly whithin the range. If you want to alter the way the individuals are initialized, we recommend overriding this method. Call the super-method from your implementation and make changes to the individuals afterwards.

For an example on how to extend `AbstractProblemDouble` you can look at the `F1Problem` class which implements a simple parabola function.

The class `AbstractProblemBinary`. The binary variant is widely analogous to `AbstractProblemDouble`. The main differences are that there is no range definition (the range is always $0^n - 1^n$) and that the template is of a different type, namely `GAIndividualBinaryData`, and delivers a Java `BitSet` as data representation instead of a double vector. The `eval` function signature changes accordingly, and to implement a target function $g_{\text{target}}(x) = y$ with $g_{\text{target}} : \{0,1\}^n \to R^m$, you need to work on a `BitSet`[2] object.

`public abstract double[] eval(BitSet bs):` Essential evaluation method. Override it to implement the desired target function $g_{\text{target}}(b)$. Make sure that the delivered solution vector is always of the same dimensionality m. Even if your problem

[2] Concerning `BitSet`: it may be valuable to note that when looping over a `BitSet`, it is preferable to use the self defined problem dimension as index limit instead of `size()` or `length()` methods of `BitSet`.

Algorithm 1 Simple `OptimizerFactory` usage example.

```
import javaeva.OptimizerFactory;
import javaeva.server.go.problems.F1Problem;

public class TestingF1PSO {
  public static void main(String[] args) {
    F1Problem f1 = new F1Problem();
    double[] sol;
    OptimizerFactory.setEvaluationTerminator(50000);
    sol = OptimizerFactory.optimizeToDouble(OptimizerFactory.PSO, f1, null);
    System.out.println("Found solution: ");
    for (int i=0; i<f1.getProblemDimension(); i++) System.out.print(sol[i] + " ");
    System.out.println();
  };
}
```

ID	Short Description
STD_ES	A standard (15,50)-Evolution Strategy.
CMA_ES	(15,50)-Evolution Strategy with Covariance Matrix Adaptation.
STD_GA	Standard Genetic Algorithm with elitism
PSO	Particle Swarm Optimization with constriction.
DE	Differential Evolution
TRIBES	Tribes: an adaptive PSO
RANDOM	Random search (Monte-Carlo)
HILLCL	Multi-start hill climbing
CL_HILLCL	Clustering multi-start hill climbing
CBN_ES	Clustering-based Niching ES

TABLE 1. Overview over algorithms accessible through OptimizerFactory.

is only one-dimensional, return an array of length 1 with the single fitness value as only field.

For an example on how to implement binary functions, look at the `B1Problem` class which realizes a simple minimize bits problem.

5.1.2. *The `OptimizerFactory`.* To access default optimization algorithms easily, we have defined an `OptimizerFactory` class. It allows to specify an optimization algorithm by an ID number and mainly takes a problem class and an optional output file as input. For example, if you have a double-valued problem class and just want to retrieve one solution, use the `optimizeToDouble(final int optType, AbstractOptimizationProblem problem, String outputFilePrefix)` method, which returns the solution as a double vector.

Table 1 gives an overview over the currently accessible optimization strategies. You may use `showOptimizers()` to get a string with a summary of the implemented algorithms with ID associations. A short example is shown in Listing 1, where PSO is used to optimize the simple hyper parabola (in 10 dimensions by default). The PSO with $50,000$ evaluations should find a solution very close to zero, e.g. absolutely below 10^{-30} in every dimension.

5.2. Termination Criteria. To configure the termination criteria of an optimization process started through the OptimizerFactory, use the setTerminator family. The default terminator stops at a maximal number of fitness evaluations, e.g. $10,000$. To change the number

of evaluations performed to, for example, 50,000, call `OptimizerFactory.setEvaluation-Terminator(50000)` before starting the optimization, as in Alg. 1.

For more flexible termination criteria, there are several Terminator parameter classes you can use and set them directly using `OptimizerFactory.setTerminator(term)`. Available are the following variants:

`EvaluationTerminator`: Construct with a maximal number of evaluations. Constructor parameters:
- `maxEval`: integer number of evaluations to be performed at maximum.

`GenerationTerminator`: Terminate after a given number of generations. As not all algorithms use constant population sizes, we suggest to use EvaluationTerminator preferably. Constructor parameters:
- `gens`: integer number of generations to be performed at maximum.

`FitnessValueTerminator`: A minimum fitness value (vector) must be reached to terminate. Construct with a double array representing the target fitness value. Constructor parameters:
- `v`: double array fitness vector to be reached.

`FitnessConvergenceTerminator`: Terminate as soon as there is hardly any improvement in fitness for a certain period of time (*stagnation period*), defined by a number of evaluations or generations. "Hardly any improvement" is defined by a threshold *th*, which can be set as absolute or relative threshold. In the absolute case, termination occurs if the best fitness changes less than *th* for a stagnation period p, meaning $\forall\, i \in \{0, 1, ...p-1\} : |f(x^*_{t-i}) - f(x^*_{t-p})| < th$, where $f(x)$ stands for the fitness at position x, while x^*_t is the best solution found in iteration t. In the relative case, termination occurs if the fitness changes less than a certain percentage for a stagnation period, meaning $\forall\, i \in \{0, 1, .., p-1\} : |f(x^*_{t-i}) - f(x^*_{t-p})| < |f(x^*_{t-p})| \cdot th$. Constructor parameters:
- `thresh`: double valued fitness threshold *th*.
- `stagnPeriod`: integer length of the stagnation period.
- `bFitCallBased`: boolean flag indicating evaluation-based or generation-based stagnation period.
- `bAbsolute`: boolean flag indicating absolute or relative *th*.

`PhenotypeConvergenceTerminator`: In analogy to the `FitnessConvergenceTerminator`, terminate as soon as there is hardly any change in the best phenotype, meaning that $\forall\, i \in \{0, 1, ...p-1\} : |x^*_{t-i} - x^*_{t-p}| < th$ for the absolute case and $\forall\, i \in \{0, 1, .., p-1\} : |x^*_{t-i} - x^*_{t-p}| < |x^*_{t-p}| \cdot th$ in the relative case. Constructor parameters:
- `thresh`: double valued phenotype norm threshold *th*.
- `stagnPeriod`: integer length of the stagnation period.
- `bFitCallBased`: boolean flag indicating evaluation-based or generation-based stagnation period.
- `bAbsolute`: boolean flag indicating absolute or relative *th*.

`CombinedTerminator`: For effective optimization, one might want to combine several criteria in a boolean way, i.e. terminate if 20,000 evaluations have been performed OR the best fitness doesn't change for 1,000 evaluations. To allow for this, we provide the `CombinedTerminator` class which just takes to terminator instances and combines them logically. Thus, terminators can be nested as required. Constructor parameters:

`t1`: First terminator to combine.
`t2`: Second terminator to combine.
`bAnd`: Boolean flag indicating whether to use conjunctive (AND, for bAnd == true) or disjunctive (OR, for bAnd == false) combination.

Notes on terminators:

- Note that the termination criterion is always checked after one iteration, i.e. one generation. This means that, if the number of evaluations is not a multiple of the population size or the population size is variable, the maximum number of evaluations may be slightly exceeded. The same holds for the stagnation time in convergence terminators.
- Concerning convergence terminators: Note that for flat plateaus in fitness space, the fitness may hardly change while there is still progress in the solution space. On the other hand, note that highly nonlinear problems may hardly change in phenotype but still change considerably in fitness. When using convergence terminators, we suggest to set the stagnation period sufficiently high.

For a usage example that creates and sets a terminator which stops if either $20,000$ evaluations have been performed or both best fitness and phenotype converged for $1,000$ evaluations, see Listing 2. After a combined termination, one might want to know why the run actually stopped. The method `terminatedBecause()` implemented in `OptimizerFactory` as well as any terminator object will return a String object describing the exact reason, while the method `lastEvalsPerformed()` informs on how many evaluations were required. The PSO in the given example should require about $5,000 - 7,000$ evaluations to meet the two convergence criteria.

5.3. Customizing the Optimization. Beyond standard optimizers, you may wish to customize optimization parameters manually or iterate over different settings in a loop. To do this, you need to access the optimization parameter structure `GOParameters` and alter its values before starting optimization. A `GOParameters` instance contains all settings required for an optimization run: target function, optimizer, random seed, post-processing options and termination criterion; basically all that is also set through the workbench window of the GUI (Sec. 2.3.1). To alter specific settings, it is sufficient to alter a `GOParameters` instance generated by the `OptimizerFactory` and then start the optimization using this altered parameter instance.

5.3.1. *Accessing Parameters.* Look at Listing 3 for an example on how to customize optimization parameters. To find out about which operators are implemented and usable, it is again easiest to check out in the GUI, where there are also short descriptions available.

Note that in example 3, there will actually be $1,050$ evaluations performed, which is the first multiple of the population size of 150 that exceeds the maximum evaluation limit of $1,000$ set. To change the population size before a run, simply set a new population of the target size. Any population will be initialized to contain the given number of individuals by the problem class, usually in a random distribution over the problem range.

5.3.2. *Setting Evolutionary Operators.* In Listing 4, a (1+5) CMA-ES is configured and run on a simple bimodal target function with the global optimum near (1.7/0) and a local one near (-1.44/0). The (1+5)-CMA-ES is powerful and will find the global optimum most of the

Algorithm 2 Terminator combination example.

```
import javaeva.OptimizerFactory;
import javaeva.server.go.problems.F1Problem;
import javaeva.server.go.operators.terminators.*;

public class TestingTerminators {
  public static void main(String[] args) {
    F1Problem f1 = new F1Problem();
    double[] sol;
    // A combined terminator for fitness and phenotype convergence
    CombinedTerminator convT = new CombinedTerminator(
            // fitness-based stagnation period, absolute threshold
            new FitnessConvergenceTerminator(0.0001, 1000, true, true),
            new PhenotypeConvergenceTerminator(0.0001, 1000, true, true),
            CombinedTerminator.AND);
    // Adding an evaluation terminator with OR to the convergence criterion
    OptimizerFactory.setTerminator(new CombinedTerminator(
              new EvaluationTerminator(20000),
              convT,
              CombinedTerminator.OR));
    sol = OptimizerFactory.optimizeToDouble(OptimizerFactory.PSO, f1, null);
    System.out.println(OptimizerFactory.terminatedBecause());
    System.out.println(OptimizerFactory.lastEvalsPerformed()
                         + " evals performed, found solution: "
                         + OptimizerFactory.terminatedBecause()
                         + " Found solution: ");
    for (int i=0; i<f1.getProblemDimension(); i++) System.out.print(sol[i] + " ");
    System.out.println();
  };
}
```

time. Sometimes, however, due to its relatively high selection pressure and elitistic strategy, it will converge in the local optimum, depending on the random initialization.

Lines 20-23 of Listing 4 show how to access evolutionary operators directly. What happens here is that the template individual delivered with the problem class (because the problem defines the representation) is modified to use the given mutation and crossover operator and probabilities. Typical for ES, the mutation probability p_m is relatively high, while the crossover probability p_C is rather low. One could also use `fm0.getIndividualTemplate().setMutationOperator(...)` etc. to set the operators and probabilities one by one just as through the GUI. Notice that not all implemented heuristics make use of individual evolutionary operators. Several come with their own operator definitions, such as DE and PSO, for example. The Hill Climbers, on the other hand, do use individual mutation but override individual probabilities to $p_m = 1$ and $p_c = 0$ by definition.

5.3.3. *A Multi-Modal Example with Post-Processing.* When looking at the last example in 5.3.2 working on a bimodal function, one might ask how to retrieve more than just one optimum of the target function. In JavaEvA 2 there are some optimizers implemented which are specialized on this task. Listing 5 shows an example using the clustering-based niching EA (CBN-EA) [**6**] and post-processing to identify both optima of the target function.

Notice that two post-processing cycles are performed (lines 26 and 33). Any repeated post-processing iteration performed on the `OptimizerFactory` uses the same initial state. This means that if there is no new optimization cycle, any new post-processing will work on the same set of solutions, namely the result population of the last optimization.

Algorithm 3 Customized optimization example.

```java
import java.util.BitSet;
import javaeva.server.go.operators.selection.SelectXProbRouletteWheel;
import javaeva.server.go.operators.terminators.EvaluationTerminator;
import javaeva.server.go.populations.Population;
import javaeva.server.go.problems.B1Problem;
import javaeva.server.go.strategies.GeneticAlgorithm;
import javaeva.server.modules.GOParameters;

public class TestingGAB1 {
  public static void main(String[] args) {
    B1Problem b1 = new B1Problem();
    BitSet sol;
    // default go-parameter instance with a GA
    GOParameters gaParams = OptimizerFactory.standardGA(b1);
    // add an evaluation terminator
    gaParams.setTerminator(new EvaluationTerminator(1000));
    // set a specific random seed
    gaParams.setSeed(2342);

    // access the GA
    GeneticAlgorithm ga = (GeneticAlgorithm)gaParams.getOptimizer();
    // set no elitism
    ga.setElitism(false);
    // set roulette wheel selection
    ga.setParentSelection(new SelectXProbRouletteWheel());
    // set population size 150
    ga.setPopulation(new Population(150));

    // run optimization and print intermediate results to a file with given prefix
    sol = OptimizerFactory.optimizeToBinary(gaParams, "ga-opt-results");
    System.out.println("Found solution: ");
    for (int i=0; i<b1.getProblemDimension(); i++) System.out.print(sol.get(i)+" ");
    System.out.println();
  };
}
```

This is useful, for example, to search for optima using different resolutions iteratively. In our example, we however just demonstrate the different results without (line 26) and with clustering (line 33). If you run the example a few times, it will happen quite often that after the first post-processing with clustering only, more than the two optima are returned, while after the second step with hill climbing, this will happen rather seldomly. All in all, CBN locates the two optima in most of the cases and post-processing helps to identify the real hot spot. In Fig. 5, two graphs show the target function and the states of an exemplary CBN-run after 500 (left) and 1,500 evaluations (right).

The FM0Problem is of course very simple, 2-dimensional and having only two optima in the defined range. For harder problems, a few thousand evaluations will not suffice, and for highly multi-modal target functions, e.g. if there are thousands or tens of thousands of local optima, things get really tough. The current implementation of CBN is able to find more optima than the population size defined, because it is able to reinitialize a converged cluster during a run, saving a representative to an archive. But for problems with a lot of deceptive optima, it might also be a good strategy to concentrate on finding one global optimum, and, as it won't be found in most of the runs, look at the results of the single-run solution set.

Algorithm 4 Setting up a (1+5) CMA-ES.

```java
import javaeva.OptimizerFactory;
import javaeva.server.go.individuals.AbstractEAIndividual;
import javaeva.server.go.operators.crossover.CrossoverESDefault;
import javaeva.server.go.operators.mutation.MutateESCovarianceMartixAdaption;
import javaeva.server.go.operators.terminators.EvaluationTerminator;
import javaeva.server.go.problems.FM0Problem;
import javaeva.server.go.strategies.EvolutionStrategies;
import javaeva.server.modules.GOParameters;

public class TestingPlusCMAES {
  public static void main(String[] args) {
    // a simple bimodal target function, two optima
    FM0Problem fm0 = new FM0Problem();
    AbstractEAIndividual bestIndy;
    // create standard ES parameters
    GOParameters esParams = OptimizerFactory.standardES(fm0);
    esParams.setTerminator(new EvaluationTerminator(2000));
    // set a random seed based on system time
    esParams.setSeed(0);

    // set evolutionary operators and probabilities
    AbstractEAIndividual.setOperators(
                    fm0.getIndividualTemplate(),
                    new MutateESCovarianceMartixAdaption(), 0.9,
                    new CrossoverESDefault(), 0.1);

    // access the ES
    EvolutionStrategies es = (EvolutionStrategies)esParams.getOptimizer();
    // set a (1+5) selection strategy
    es.setMu(1);
    es.setLambda(5);
    es.setPlusStrategy(true);

    // run optimization and retrieve winner individual
    bestIndy = (AbstractEAIndividual)OptimizerFactory.optimizeToInd(esParams, null);
    System.out.println("Found solution: "
                    + AbstractEAIndividual.getDefaultDataString(bestIndy));
  };
}
```

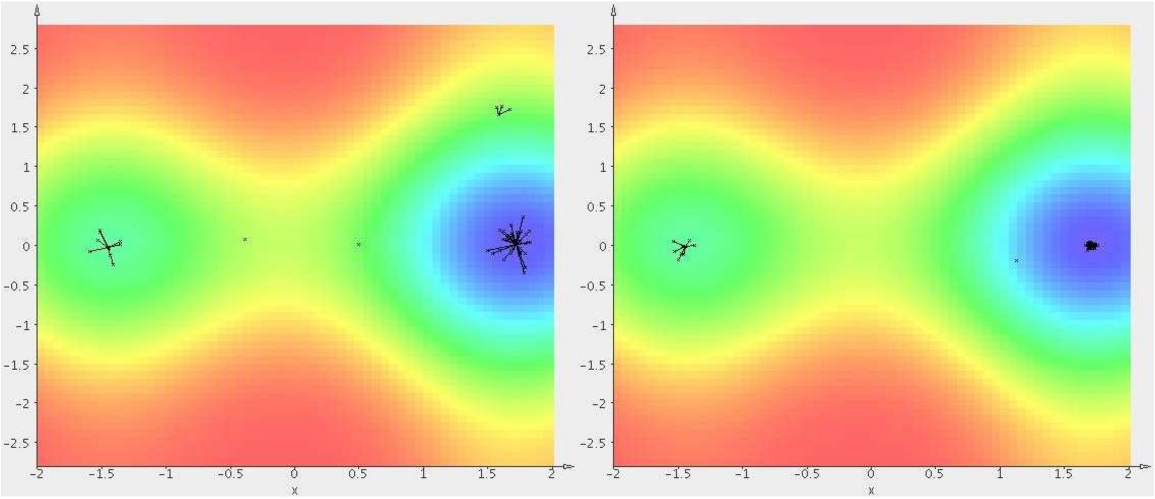

FIGURE 5. Exemplary states of CBN on the simple bimodal FM0Problem.

Algorithm 5 Solving a bimodal function with CBN and post-processing.

```java

import java.util.Vector;
import javaeva.OptimizerFactory;
import javaeva.server.go.individuals.AbstractEAIndividual;
import javaeva.server.go.operators.postprocess.PostProcessParams;
import javaeva.server.go.operators.terminators.EvaluationTerminator;
import javaeva.server.go.problems.FM0Problem;
import javaeva.server.modules.GOParameters;

public class TestingCBNPostProc {
  public static void main(String[] args) {
    FM0Problem fm0 = new FM0Problem();
    AbstractEAIndividual best;
    Vector<AbstractEAIndividual> ppSols;

    GOParameters esParams = OptimizerFactory.cbnES(fm0);
    esParams.setTerminator(new EvaluationTerminator(2000));
    esParams.setSeed(0);

    best = (AbstractEAIndividual)OptimizerFactory.optimizeToInd(esParams, null);

    System.out.println("Found solution: "
            + AbstractEAIndividual.getDefaultDataString(best));

    // post-process with clustering only
    ppSols = OptimizerFactory.postProcessIndVec(new PostProcessParams(0, 0.1, 5));
    System.out.println("After clustering: ");
    for (AbstractEAIndividual indy : ppSols) {
      System.out.println(AbstractEAIndividual.getDefaultDataString(indy));
    }

    // post-process with clustering and hill climbing
    ppSols = OptimizerFactory.postProcessIndVec(new PostProcessParams(1000, 0.1, 5));
    System.out.println("After clustering / HC: ");
    for (AbstractEAIndividual indy : ppSols) {
      System.out.println(AbstractEAIndividual.getDefaultDataString(indy));
    }
  };
}
```

6. Further Reading

As noted before, this introduction does not cover algorithmic basics. We recommend the introduction of Engelbrecht [**3**] for newer strategies (DE, PSO) and Bäck et al. [**2**] for classical Evolutionary Algorithms. To go into details of JavaEvA 2, a look on the technical report of 2005 [**7**], though slightly outdated, will still be helpful, as well as the thesis of Felix Streichert [**5**], one of the main authors of JavaEvA.

Bibliography

[1] JavaEvA Project Homepage. `http://www.ra.cs.uni-tuebingen.de/software/JavaEvA`. Accessed in March 2008.

[2] Thomas Bäck, David B. Fogel, and Zbigniew Michalewicz, editors. *Evolutionary Computation 1: Basic Algorithms and Operators*. IOP Publishing Ltd., Bristol, UK, UK, 1999.

[3] Andries Engelbrecht. *Computational Intelligence: An Introduction, 2nd Edition*. Halsted Press/Wiley, New York, NY, USA, 2007.

[4] Martin Ester, Hans-Peter Kriegel, Jörg Sander, and Xu Xiaowei. A density-based algorithm for discovering clusters in large spatial databases with noise. In *2nd International Conference on Knowledge Discovery and Data Mining*, page 226.

[5] Felix Streichert. *Evolutionary Algorithms in Multi-Modal and Multi-Objective Environments*. PhD thesis, University of Tübingen, Germany, 2007.

[6] Felix Streichert, Gunnar Stein, Holger Ulmer, and Andreas Zell. A clustering based niching method for evolutionary algorithms. In *Evolution Artificielle, Proceedings of the 6th International Conference (EA 2003)*, page 293.

[7] Felix Streichert and Holger Ulmer. JavaEvA - a java based framework for evolutionary algorithms. Technical Report WSI-2005-06, Universitätsbibliothek Tübingen, Germany, 2005.

[8] Jürgen Wakunda and Andreas Zell. EvA - a tool for optimization with evolutionary algorithms. In *Proceedings of the 23rd EUROMICRO Conference*, Budapest, Hungary, 1997.
