# Supplementary material for: optPBN: An Optimisation Toolbox for Probabilistic Boolean Networks
Source: PLoS One. 2014 Jul 1;9(7):e98001. doi: 10.1371/journal.pone.0098001 (PMC4077690; doi:10.1371/journal.pone.0098001)
Supplement: File S3 — Manual of the optPBN toolbox. The PDF manual provides a detailed description of the optPBN optimisation pipeline. A step-by-step guideline on how to use the optPBN toolbox together with the explanation of the core idea for each computational script is provided in the document. (PDF) [file pone.0098001.s004.pdf]

Manual of optPBN toolbox

This manual was aimed to give users the general concept of the pipeline for the optimisation of probabilistic Boolean network (PBN) employing *optPBN* toolbox. In this manual, we demonstrate the pipeline script for the optimisation of case study 2 by particle swarm optimisation algorithm (PSO) as an example for detailed explanation. The main concept and output(s) of each function in the pipeline will be explained step-by-step where it should give users sufficient information to understand and apply the functions properly. From this point on, the exemplary pipeline script will be divided into multiple parts and will be shown in the green boxes. The explanation and the outcome from the respective parts will be presented below the scripts.

Modes of optimisation, discretised level(s) of probabilities, and toggle switch of optimisation process

```
% Optimisation parameters
Optimization=0; % 0 = No optimization (review result), 1 = optimize
Mode=2; % 1 = Discrete mode, 2 = Continuous mode
Discretize_level=2; % Two discrete values (0,1) - default setting
opt_iterations=5000; % number of iterations during optimisation
```

optPBN can be operated in two different optimisation modes, 'discrete' and 'continuous'. The optimisation in 'discrete mode' only explores a certain number of probability levels. For instance, assigning 2 levels of probability (default setting) would limit the explored state space of probability to only 0 and 1. This optimisation mode is suitable for a rapid identification of corresponding Boolean rules from a given set of predefined rules in Boolean networks e.g., in case study 1 and in the original toy model from Saez-Rodriguez *et al.*, i.e. case study 3 [1].

On comparison, the continuous state space of probability from 0 to 1 is explored in 'continuous mode'. An intensive explorative of state space by the optimisers allows *optPBN* to identify the corresponding selection probabilities in PBNs based on the given set of normalized data e.g. in case study 2, in the modified toy model of Saez-Rodriguez *et al.*, i.e. case study 3 and in a modified PBN model of apoptosis adapted from Schlatter *et al.*, i.e. case study 4 [2].

In case the optimisation will be performed (Optimization=1), then the number of iterations during the optimisation process can also be assigned to the parameters 'opt_iterations'. About 5000 iterations are recommended for general optimisation problem.

In order to facilitate the setting of optimisation process, the assignments of optimisation mode and discretised probability levels can be assigned at the beginning of the script. In addition, a toggle switch option to bypass the optimisation process is also given so that the users can reuse the same pipeline to review the optimisations results which was already performed. A set of result files which have been presented in the manuscript for each model is also provided in Supporting Information: File S2.

Approaches to check for the convergence of steady-state distributions and corresponding parameters

```
% Select an approach to check for convergence of steady-state distribution

Approach=1; % 1 = Main (ergodic PBN+TSMC), 2 = Alternative (PBN without
perturbation+random IC)

if Approach==1
    % If Approach==1, please choose Two-state Markov Chain parameters and
perturbation scheme
    epsilon=0.001; % range of transition probability [Default=0.001]
    r=0.025; % range of accuracy (most sensitive) [Default=0.025]
    s=0.95; % probability to acquire defined accuracy [Default=0.95]
    p=0; % perturbation in Shmulevich's scheme [Default=0, 0%]
    pMir=0.001; % perturbation in Miranda's & Parga's scheme [Default=0.001,
0.1%]
    initial_nsteps=100; % initial simulation steps [Recommended at 100 steps
for n < 100]
elseif Approach==2
    % If Approach==2, please choose parameter related to Monte-Carlo
simulation
    iterations=10; % number of iterations (initial condition samplings)
    nsteps=50; % number of time steps
end
```

In the *optPBN* pipeline, we also offer two approaches to approximate steady-state distributions of output nodes. The first approach, namely the 'main approach', employed ergodic PBN (obtained by introducing perturbation parameter, 'pMir' in Miranda and Parga scheme [3]) followed by using two-state Markov chain (TSMC) method to check for the initial time steps before reaching steady-state distribution (m0) and for the required time steps of simulation to acquire marginalised steady-state distributions of output nodes at a desired accuracy (N). Another approach, namely the 'alternative approach', approximate steady-state distributions of output nodes in PBN without perturbation by starting with multiple random initial conditions and then perform Monte-Carlo simulation with sufficient time steps (users' pre-defined) until reaching steady-state.

The two approaches for the approximation of steady-state distribution can be chosen by defining the corresponding number on the 'Approach' variable. For each of the approaches, additional parameters related to the chosen approach can be adjusted. For instance, the TSMC parameters (epsilon, r, s, and initial time steps) and perturbation parameters (p and pMir) can be adapted in the main approach to fit users' desired accuracy. The most sensitive parameter in this approach is 'r' which has the correlation with 'N' as follow "$r \propto 1/N^2$". The lower 'r' is, the longer simulated time steps 'N' would be. This setting resulted in a better approximation of steady-state but will also increase the computational time respectively. More details on these parameters can be found in [4,5]. In parallel, the fixed numbers of initial condition samplings (iterations) and of time steps (nsteps) can also be defined for the alternative approach.

Building a global parameter, storing model structures and measurement data for optimisation

```
%% Define model structure and different experiments

% Assign estim as global parameter
global estim

% Adjust parameter for optimization and store parameters in estim
estim.Approach=Approach;
if Approach==1
    estim.epsilon=epsilon;
    estim.r=r;
    estim.s=s;
    estim.p=p;
    estim.pMir=pMir;
    estim.iterations=1;
    estim.nsteps=initial_nsteps;
elseif Approach==2
    estim.iterations=iterations; %maximal number of simulation in each round
    estim.nsteps=nsteps; %number of time steps for simulation
end
estim.NrExps=0;%number of initial experiment (automatically changed)
estim.fileName='tempFIT_Case_study_2.mat'; %for saving optimization results
estim.display=1; %display output optimization intermediates?
estim.thresh=0.01; %threshold for accepting fit
estim.stopAtFirst=0; %stop when reaching the first good fit (below threshold)

% Mode of optimization
if Mode==1
    estim.optLevels=Discretize_level; %use probability levels;
elseif Mode==2
    estim.optLevels=0; %No discretization; '0' = continuous probabilities
end
```

In order to generate an integrative optimisation problem to be solved by various solvers, a global parameter (or variable) called ‘estim’ is created. This ‘estim’ parameter will be used to store all model structures and measurement data as well as necessary parameters related to the optimisation which will then be passed into the optimisation process. Several options of optimisation process can be stored in the ‘estim’ parameter as shown in the script.

Apart from using ‘estim’ to collect optimisation parameters, details on the other options are explained as follow: ‘estim.NrExps’ refers to the number of experimental conditions during the optimisation (will be automatically assigned later). The ‘estim.fileName’ option refers to the name of the output file in matrix format (.mat) which will be generated from the optimisation pipeline. This result file can be reused later for further statistical analyses. The ‘estim.display’ is set to display the intermediate result during optimisation. The ‘estim.thresh’ option is set to choose a threshold in order to stop the optimisation process and to filtrate for a numbers of good parameter sets (will be discussed later). The ‘estim.stopAtFirst’ option is set to stop the process when there is at least one parameter set with the

‘optimal cost’ (this term will be explained and discussed later) that passes below the threshold. Last, the ‘estim.optLevels’ option, which defines the mode of optimisation, will automatically be assigned based on the settings at the top of the script.

```
% Define number of experiments to optimize
optimizeExp=1:4;

% Generate different experiments in a for-loop
for counter=optimizeExp
    rules={};
    switch counter
        % Define different model structures and measurements for each case
        case 1
            % PI3K
            rules=[rules; {'PI3K = 0','1'}];
            % PTEN
            rules=[rules; {'PTEN = 0','1'}];
            measStates={'PIP3'};
            meas=[0];
        case 2
            % PI3K
            rules=[rules; {'PI3K = 1','1'}];
            % PTEN
            rules=[rules; {'PTEN = 0','1'}];
            measStates={'PIP3'};
            meas=[1];
        case 3
            % PI3K
            rules=[rules; {'PI3K = 0','1'}];
            % PTEN
            rules=[rules; {'PTEN = 1','1'}];
            measStates={'PIP3'};
            meas=[0];
        case 4
            % PI3K
            rules=[rules; {'PI3K = 1','1'}];
            % PTEN
            rules=[rules; {'PTEN = 1','1'}];
            measStates={'PIP3'};
            meas=[0.6];
    end
```

According to the experimental conditions in case study 2, four model cases are assigned based on the combination of the two inputs using ‘switch’ function. In each model case, different model structures (i.e., different state of PI3K and PTEN in this case) are assigned together with the corresponding output state(s) and measurement data. Users can choose to perform the optimisation process on all experimental cases or on only selected experiment(s) by defining the ‘optimizeExp’ variable.

The molecular interactions in the model are defined in a simple Boolean rule format. The model structure in each rule is stored in the ‘rules’ variable, appending one-by-one. The name(s) of the measured state(s) is stored in ‘measStates’ variable and the respective measurement data are stored in the ‘meas’ variable. Different structures of each model case and the corresponding measurement data generated from the ‘switch’ function are then put in a for-loop.

```
% ---------------------------------
% Optimizing nodes
% ---------------------------------

% PIP3
rules=[rules; {'PIP3 = PI3K','1'}]; % Positive interaction from PI3K
rules=[rules; {'PIP3 = PTEN','1'}]; % Positive interaction from PTEN
rules=[rules; {'PIP3 = PI3K & ~PTEN','1'}]; % Positive interaction from PI3K
and negative interaction from PTEN
rules=[rules; {'PIP3 = 0','1'}]; % No interaction

% Convert rules to PBN parameters via rule2PBN function
[n,nf,nv,F,varF,cij,state_names]=rule2PBN(rules)
```

Apart from the subset of rules which define different model structure in each model case, the Boolean rules for the other model structures which are the same in every model case are placed outside the ‘switch’ function but are still inside the for-loop. At the end of the ‘rules’ assignment, a collection of ‘rules’ will be converted into a BN/PBN model by the function ‘rule2PBN’ and the model will be stored as BN/PBN toolbox’s internal variables [6].

The BN/PBN toolbox defines the followings internal variables: n = number of nodes, nf = number of interactions for each node, nv = number of variables that involve in each function, F = all possible results from each interaction function (truth table), varF = the variables’ positions which involve in Boolean function, cij = the selection probabilities of each Boolean rule, state_names = names of the nodes in the model. An extracted output from the rule2PBN script for case 4 of case study 2 (PI3K = 1 and PTEN = 1) is shown in the blue box below. For more explanation on each parameter in BN/PBN toolbox’s internal format, please refer to the help file of ‘pbnRnd.m’ in BN/PBN toolbox.

```
n =

     3

nf =

     1     1     4

nv =

     0     0     1     1     2     0

F =

     1     1     0     0     0     0

     1     1     1     1     0     0

    -1    -1    -1    -1     1    -1

    -1    -1    -1    -1     0    -1

varF =

     1     1     1     2     1     1

    -1    -1    -1    -1     2    -1

cij =

     1     1     1

    -1    -1     1

    -1    -1     1

    -1    -1     1
```

```
% Specify initial conditions
ics=[]; % no specification
% ics=[1:n; zeros(1,n)]' % all initial conditions are 0

% Choose nodes to be optimized
% optimizeStates=state_names; % For all nodes with more than 1 interaction
function
optimizeStates={'PIP3'};  % For certain nodes

% Add up all parameters in estim global parameter format
estim=add2estim(estim,n,nf,nv,F,varF,cij,state_names,optimizeStates,ics,measS
tates,meas)

end

% Preprocessing estim parameter (sorting) to generate common optimization
problem
estim=preprocessMultiExp(estim);

paramNr=estim.paramNr; % Extract parameter numbers from estim for further use
```

Next, the initial condition of each state can be assigned in the 'ics' variable and the nodes to be optimised can be set in the 'optimizeStates' variable (i.e., PIP3 in this study). Once all necessary components of the model were provided, the script 'add2estim' will use the global parameter 'estim' to store all variables that were generated from each experimental case and will subsequently save each of them as a separated experiment (listed as 'exp') inside 'estim'.

The pipeline script as presented until this point is operated in a large for-loop so that a number of model cases will be generated according to the assigned 'optimizeExp' variable. After the operation in the for-loop is complete, the 'estim' global parameter will be processed again by the function 'preprocessMultiExp'. The respective function will collect, sort, and combine the model states which will be optimised in each experimental case in order to make a common list for all model cases. After the pre-processing, the 'estim' parameter is now considered as an integrated optimisation problem which can be solved by optimisation algorithms. The 'estim' parameter in the form of an integrated optimisation problem for case study 2 is shown in the blue box below.

```
estim =

        Approach: 1

         epsilon: 1.0000e-03

               r: 0.0250
```

```
s: 0.9500

p: 0

pMir: 1.0000e-03

iterations: 1

nsteps: 100

NrExps: 4

fileName: 'tempFIT_Case_study_2.mat'

display: 1

thresh: 0.0100

stopAtFirst: 0

optLevels: 0

exp1: [1x1 struct]

exp2: [1x1 struct]

exp3: [1x1 struct]

exp4: [1x1 struct]

optimizeStates: {'PIP3'}

paramNr: 4

paramNrList: 4
```

As shown, all optimisation options which were assigned previously are stored in the global parameter 'estim'. Also, the details of each model case are stored in the sub-parameters 'exp1' to 'exp4' inside 'estim'. Each 'exp' parameter stored the model structure which is different in each experimental case together with respective measurement data and initial conditions. The last part of the output displays the common optimised states, 'optimizeStates', the number of all parameters which will be optimised, 'paramNr', and the number of parameters which are involved in each optimise state, 'paramNrList'.

In case study 2, there are four selection probabilities to be optimised for PIP3. As there is only one node to be optimised in this model, the values of 'paramNr' and 'paramNrList' are the same. If there is more

than one node to optimised, all numbers of selection probabilities to be optimised will be added up and shown in ‘paramNr’ while the numbers of optimised selection probability for each node to be optimised will be shown in ‘paramNrList’ as a series of numbers.

Optimisation of the integrated optimisation problem with particle swarm optimisation algorithm (PSO)

```
%% Optimization

if Optimization==1

    tic

    % Delete previous temp file for fitting
    delete(estim.fileName)

    % Define parameter OPTIONS for optimization
    OPTIONS.maxfunevals=opt_iterations; %number of iterations cycles
    OPTIONS.highbounds=ones(1,paramNr);%set higher bound of parameter values
    OPTIONS.lowbounds=zeros(1,paramNr);%set lower bound of parameter values

    % Choose method for optimization (apply particle swarm algorithm for the
optimization here)
    [optParams,FVAL,EXITFLAG] = pswarmSB(@opt_fun,zeros(1,paramNr),OPTIONS)

    Elapsed_time=toc
    save(estim.fileName,'Elapsed_time','-append')

end
```

After assigning the ‘estim’ global parameter to collect all necessary information from the model, it can then be passed into the optimisation section once the toggle switch of the optimisation process is ON (optimization=1). At the beginning of this section, the previous output file will be deleted to ensure that the new result from the optimisation process will be stored as a new output file. Additional options for the optimisation process with particle swarm algorithm can later be customised as follow: the numbers of sampling parameter sets to be optimised, ‘OPTIONS.maxfunevals’ (which will be set according to the pre-defined value at the beginning of the script) and the higher and lower bounds of parameter values, ‘OPTIONS.highbounds’ and ‘OPTIONS.lowbounds’. Because the parameters to be optimised are indeed the selection probabilities of Boolean rules, the range of optimised parameters values will be between zero and one. Therefore, the higher bounds are set to 1 and lower bounds are set to 0 respectively.

The next part of this section contains the principal function for the optimisation process. In the stand-alone version of *optPBN* toolbox, particle swarm global optimisation, ‘pswarmSB’ [7], from the Systems Biology Toolbox 2 (SBTB2) [8,9] is used as the optimiser. The inputs for ‘pswarmSB’ are the objective

function, ‘opt_fun’, initial guess of probability parameters (if applicable), and the optimisation options ‘OPTIONS’ as previously defined.

The ‘opt_fun’ function provides an interface between the integrated optimisation problem as defined in ‘estim’ and the optimisation solver (i.e., ‘pswarmSB’ in this case). The chosen approach to approximate steady-state distributions of output nodes (main or alternative approach) will be applied accordingly. Based on the setting as defined in the script, approximately 5,000 parameter sets will be generated and fitted against experimental data. The results from the optimisation process will be saved in the filename 'tempFIT_Case_study_2.mat' which can be reused for further statistical analyses. Once the optimisation process is complete, the computational time is recorded (with tic-toc function) and is also saved in the result file. The initial results from the optimisation process are reported as shown in the blue box below.

```
Elapsed time is 1204.876829 seconds.

Maximum number of iterations or objective function evaluations reached

   IterCounter: 65

 ObjFunCounter: 5061

     PollSteps: 12

 SuccPollSteps: 1

optParams =

 0.4974      0   0.3260      0

FVAL =

  0


EXITFLAG =

   IterCounter: 65

 ObjFunCounter: 5061

     PollSteps: 12

 SuccPollSteps: 1
```

As shown, the time taken for the optimisation process is firstly displayed. Then, the best set of raw parameter values obtained from the optimisation is stored in 'optParams' variable. These raw parameter values were internally converted into the selection probability (cij) values for each Boolean function by the 'opt_fun' script before it was passed to be evaluated with the objective function. During the conversion, raw parameter values were scaled up or down so that the sum of all parameter values is equal to one (represent the sum of all probabilities). For instance, the values 0.4974 and 0.3260 in 'optParams' were scaled up to 0.6041 and 0.3959 inside the 'opt_fun' script respectively during the evaluation.

The parameter set shown in 'optParams' is the best parameter set identified from approximately 5,000 sampling parameter sets with the lowest objective function, 'FVAL'. The 'FVAL' value or so called 'optimal cost' refers to the cumulative addition of sum of squared error (SSE). The SSE value represents the difference between simulated steady-state distributions of output states generated from the sampling parameters compared to measurement data as demonstrated in equation 1.

$$SSE = \sum_{i=1}^{n} (simulated - measurement)^2 \text{ .... (eq. 1)}$$

In general, the lower optimal cost is, the better sampling parameter set gives model outputs that matches to the measurement data. The optimal cost is therefore an indicator to determine the quality of model fitting. The remaining parameters i.e., 'EXITFLAG' shows the conditions which were reached to exit the optimisation process. As shown, the 'ObjFunCounter' parameter shows the final counter number of parameter set which were sampled. In this optimisation run, 5,061 parameter sets were sampled, a bit exceeded the assigned number at 5,000. For the explanations on 'IterCounter', 'PollSteps' and 'SuccPollSteps' parameters, please refer to the documentation of 'pswarmSB' in SBtoolbox2 [7-9].

Model fitting's quality evaluation and preliminary interpretation of the result

```
%% Identification history: identify best parameter set etc.
[bestOptFuncValue,bestRun,bestx]=bestParams(estim,~estim.stopAtFirst)

%% Generate optimal cij & Generate one empirical solution
simFlag=1; %simulate?
cijs=evalCijAndSimulate(estim,bestRun,simFlag);
```

Once the optimisation process is complete and a set of optimised parameters are generated and stored as internal variables. The recently generated internal variables of the result can be directly used for further analysis, or, the analysis can later be performed in another session by loading the result file (.mat) which contains these variables. Since 'estim' is required in the pipeline of analysis, it has to be recreated again if the analysis will be performed in a separated session from the optimisation. This can

be done by toggle off the optimisation switch at the beginning of the script (optimization=0) and run all scripts until reaching the analytical part shown in the green box above.

In the analytical section of the pipeline, the 'estim' global parameter is used to display the history of the optimisation process with the 'bestParams' function. This function returns the following outputs: 'bestOptFuncValue' which shows the optimal cost of the best identified parameter set (equal to FVAL value), 'bestRun' which shows the index of the best parameter set (e.g., the 848th set), and 'bestx' which shows the raw parameter values of the best parameter set (same as shown in the 'optParams' parameter).

As most of the outputs from 'bestParams' function are similar to the previous ones, the printed outputs of this section are not shown here. In case the estim.stopAtFirst option was not active (estim.stopAtFirst=0), the unsorted 'FVAL' values of all optimised parameter sets during the optimisation will be plotted together with the sorted one. The unsorted and sorted FVAL plots and the zoom-in view of the sorted plot for this case study are shown below.

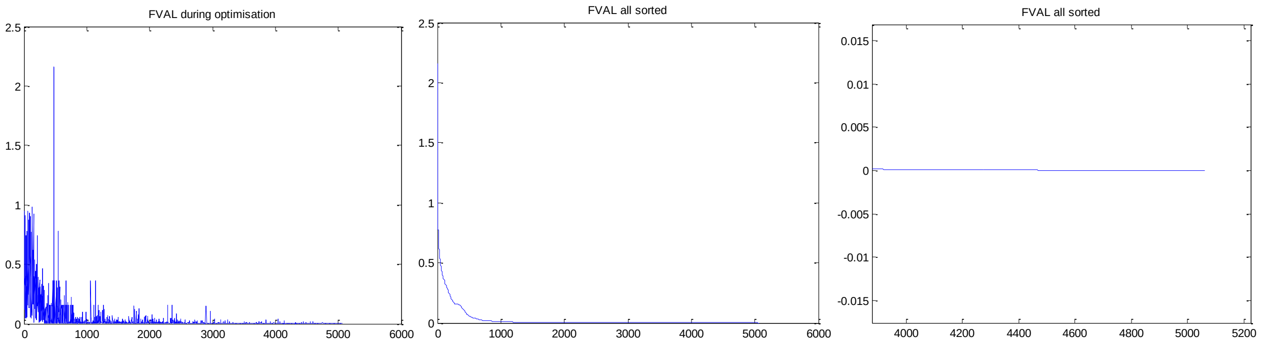

From the plots, the optimal costs range from approximately 0 to 2.16. These plots provide a general idea on how the optimal solutions are converged during the optimisation process. Also, it gives a first clue on how to set an appropriate threshold to select only some good parameter sets for further analyses (this topic will be discussed later the next section of the script).

The next function, 'evalCijAndSimulate', allows for evaluating selection probability (cij) and for simulating the model based on raw parameter values. The output of the function is a common set of optimised selection probabilities (cijs) that fits to all experimental cases. If simulation will be performed (simFlag=1, default setting), the options related to simulation in 'estim' as well as the model structures and measurement data are reused for simulation.

Once the 'cij' values for each Boolean interaction have been evaluated, the function 'pbnStationary_TS' is called inside the 'evalCijAndSimulate' function to approximate steady-state distributions of model states in the form of probability distribution function (pdf). In general, 'pdf' describes the relative likelihood of a random variable to occur at a given time point. In the context of our study, we assume that the calculated 'pdf' refers to the random state values in steady-state. This can be approximated in the main approach by taking the mean of random values which have already entered steady-state (after

m0 time steps) for minimal required number of steps to acquire pre-defined accuracy (N). This step requires an additional script ‘TwoStateMC_MargDist.m’ to determine a set of parameters from Markov chains (alpha and beta) in order to calculate m0 and N for the approximation of marginal distributions of output genes (see more detail in [4,5]). For the alternative approach, pdf can be approximated by perform Monte-Carlo simulation for a longer time period and taking the mean of only second half of simulated results as steady-state distributions.

In the report of ‘evalCijAndSimulate’ function, the calculated ‘pdf’ values (as predicted mean state value at steady-state) of each optimised node are compared against the measurement data for each model case to determine the quality of model fitting. The sum of squared differences between the predicted state values and the measurement data (i.e., SSE) are displayed accumulatively after the evaluation on each model case. To provide a short overview on the output of ‘evalcijAndSimulate’ function, the result from case 3 and case 4 (experiments 3 and 4) which refers to the results from ‘only input from PIP3’ and ‘both inputs (PIP3 and PTEN)’ conditions are shown in the blue box below.

```
Run Nr 848 used.

cij =

    1.0000    1.0000    0.6041

   -1.0000   -1.0000         0

   -1.0000   -1.0000    0.3959

   -1.0000   -1.0000         0


Number of nsteps = 101

EXPERIMENT 3:

    'State'    'Model'    'Meas'

    'PI3K'     [    0]    [ NaN]

    'PTEN'     [    1]    [ NaN]

    'PIP3'     [    0]    [   0]

acc_diff =

     0
```

```
Number of nsteps = 2032

EXPERIMENT 4:

    'State'    'Model'     'Meas'

    'PI3K'     [     1]    [   NaN]

    'PTEN'     [     1]    [   NaN]

    'PIP3'     [0.6083]    [0.6000]

acc_diff =

   6.8355e-05
```

If the main approach was applied, the number of time steps used to approximate steady-state distributions (N) for each model case will be shown. Then, the predicted states values of the molecules in the column 'Model' are directly compared to the measurement data in the column 'Meas' for each model case. The differences between these two columns are sum up to the accumulated optimal cost shown in the parameter 'acc_diff'. The predicted state values of all molecules are simulated based on the best parameter set. For the states without measurement data, the measurement is shown as Not a Number ('NaN') and they are not included into the calculation of accumulated optimal cost.

As an additional outcome, the 'cijs' values (same as 'cij' value, not shown) display the best common selection probabilities for each Boolean function of each molecule in the model. For example, the third column of 'cij' refers to 'PIP3'. Therefore, the first Boolean function which is 'PIP3=PI3K' has the selection probability of 0.6041. This infers that the weight of PIP3 activation solely by PI3K is roughly 60% from the overall activation. In parallel, the third function, 'PIP3=PI3K & ~PTEN', with the selection probability of 0.3959 infers that the weight of PIP3 activation by PI3K which is simultaneously inhibited by PTEN is approximately 40%. The rest of the interaction functions (second and fourth) with selection probabilities equal zero infers that the respective Boolean rules ('PIP3=PTEN' and 'PIP3=0') should not be included in the model as they are not corresponding to the given experimental data.

Even though we demonstrated that the weight of interaction from each input on PIP3 can be preliminary determined, these selection probabilities are derived from only one set of parameters. We should also get an overview of the results from a set of fitted parameters by examining their distributions to assess the identifiability. The process for selecting only good parameter sets can be achieved by setting a filtering threshold or to simply define and include the exact amount of best parameter sets in term of optimal cost. The selected parameters are then used to perform statistical analyses, i.e., the calculation of mean and standard deviation (S.D.) in our pipeline, for further analysis and interpretation. The scripts to perform such analysis are shown in the green box below.

Statistical analysis of multiple optimised parameter sets and interpretation

```
%% Statistics for best runs (bestOptFuncValue threshold)
% Use fold of bestOptFuncValue as threshold off point
% thresh=1.5*bestOptFuncValue
% threshRelative=1 %relative (or absolute) threshold?

% Use relative threshold as cut off point
thresh=estim.thresh; %refer to predefined threshold in estim
threshRelative=0; %relative (or absolute) threshold?

% Use certain number of the best runs; [] = use threshold value
SelectedNrRuns=500;

% Calculate the best mean and best distribution as final result
[cijMeans,cijStds,bestxMean,cijsAll]=bestRunsStat(estim,thresh,threshRelative
,SelectedNrRuns);
```

The filtering cut-off to obtain good parameter sets can be assigned by using either the fold of 'bestOptFunValue', the relative threshold value, or to select a precise number of best parameter sets. For setting up a threshold, the criteria for choosing the threshold are rather subjective so it depends on the perspective of the person who analyses the data. For instance, we might set a threshold at 0.0005 so that 1,992 out of 5,047 parameter sets will be selected for further analyses (not shown). In parallel, the pipeline also offers an option to select an exact number of parameter sets with the lowest optimal cost for further statistical analysis by assigning the parameter 'SelectedNrRuns'.

In this case study, 500 optimised parameter sets (top 10%) will be chosen for further statistical analysis. This process can be done by assigning the number '500' on 'SelectedNrRuns' parameter and run the 'bestRunsStat' function. The results from the respective function yield four outputs; 'cijMeans', which are the means of selection probabilities from the selected parameter sets, 'cijStds', which are the standard deviations of selection probabilities from the selected parameter sets, 'bestxMean' which is the mean of raw parameter values from the selected parameter sets, and 'cijsAll' which are the list of all selection probabilities. Also, the mean of optimal costs from the selected 500 parameter sets is displayed in 'MeanFVAL'. The outputs of this script are shown as follow:

```
MeanFVAL =

1.3553e-05

NrRunsIncluded =

  500

bestxMean =

   0.4907    0.0000    0.3249    0.0000
```

After obtaining the selected parameter sets, the identifiability of parameters can be assessed by plotting their distributions. Also the mean of selected parameter sets can also be re-simulated e.g., 100 times to evaluate its quality. The scripts for performing such analyses are displayed as follow:

```
%% Plot distribution of optimised parameters

PlotModelState=[3]; % PIP3
ChosenBoolRules=[1,2,3,4];

cijs1=[];cijs2=[];cijs3=[];cijs4=[];

for counter=1:length(PlotModelState)
    for counter2=1:size(cijsAll,4)
        current_cijs=cijsAll(:,:,1,counter2);
        cijs1=[cijs1;
current_cijs(ChosenBoolRules(1),PlotModelState(counter))];
        cijs2=[cijs2;
current_cijs(ChosenBoolRules(2),PlotModelState(counter))];
        cijs3=[cijs3;
current_cijs(ChosenBoolRules(3),PlotModelState(counter))];
        cijs4=[cijs4;
current_cijs(ChosenBoolRules(4),PlotModelState(counter))];
    end
end

figure
axis([-0.1,1.1,-0.1,1.1])
hold on
plot(cijs1,cijs3,'b*','MarkerSize',10)
% plot(cijs2,cijs4,'r*','MarkerSize',10)
BestCij=cijs(:,:,1);
plot(BestCij(1,PlotModelState),BestCij(3,PlotModelState),'r*','MarkerSize',10
)

title('Scatter plot of the fitted parameter values (Case study 2)')
xlabel('$c_1^{(PIP3)}$','interpreter','latex')
ylabel('$c_3^{(PIP3)}$','interpreter','latex')

legend({'$c_1^{(PIP3)}$ (PIP3=PI3K) vs $c_3^{(PIP3)}$ (PIP3=PI3K $\land\lnot$
PTEN)'},'interpreter','latex')

%% Generate histogram of the optimal cost from bestxMean (or bestx) value
% histBestX(bestx,10)
histBestX(bestxMean,100);
title('Fitting cost evaluation of bestxMean (Case study 2)')
h = findobj(gca,'Type','patch');
set(h,'FaceColor',[.6 .6 .6],'EdgeColor','w')
set(gca,'XTickLabel',num2str(get(gca,'XTick').'))
legend(['Best fitting cost = ' num2str(bestOptFuncValue)])
```

As shown, users could plot the distribution of selected selection probabilities by extracting the values from 'cijsAll' variable. Also, the identified mean value of selected parameter sets (bestxMean) can be used to re-evaluate optimal costs which are subsequently plotted as a histogram by applying the script 'histbestX'. The figures generated from the script in this section are displayed below:

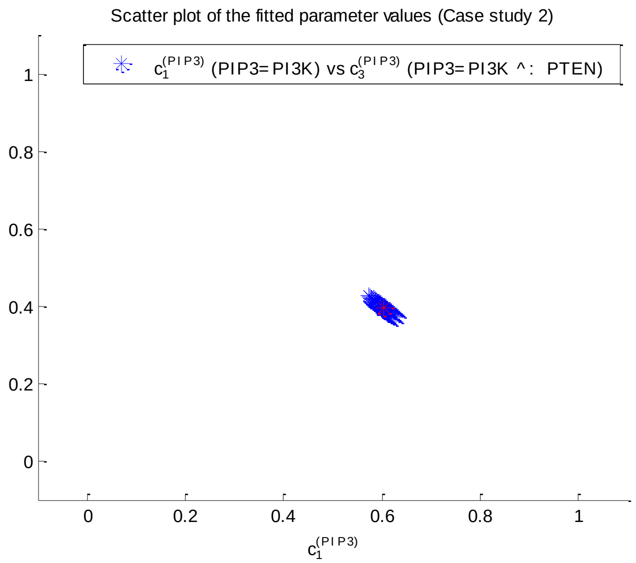

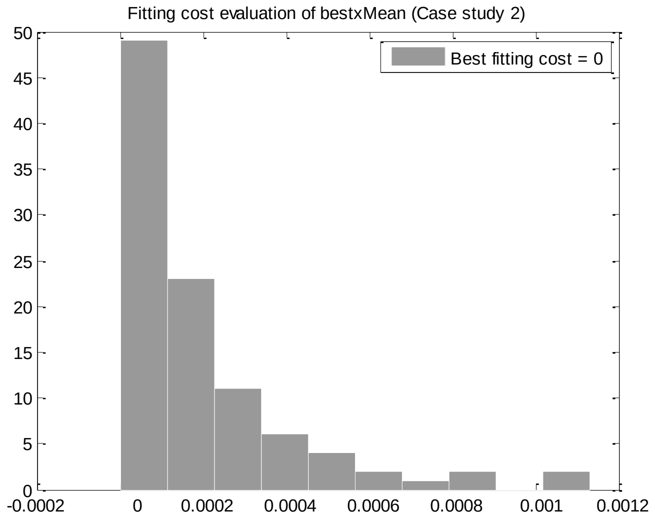

The parameter distributions of output node(s), i.e. PIP3 in this case, for all four assigned Boolean functions are shown in the left figure and the best run was marked with a red asterisk. As seen, only selection probabilities of first and third Boolean functions (cij1, $C_1^{(PIP3)}$ and cij3, $C_3^{(PIP3)}$) have non-zero values and the sum of the two are one. This indicates that the selection probabilities of second and fourth Boolean functions (cij2, $C_2^{(PIP3)}$ and cij4, $C_4^{(PIP3)}$) are practically zero value. The cluster of cij1 ($C_1^{(PIP3)}$) and cij3 ($C_3^{(PIP3)}$) parameters infers that these two parameters are identifiable as their distributions are not sparse. Therefore, the mean values of these parameters should still fall into the centre of the cluster and are justified for further interpretation. However, once identified parameter values are sparse (e.g., in case study 4), the interpretation from the best run is much preferred as the mean values might not always fall in the solution space and they might in turn lead to wrong interpretations.

On the right figure, the model was re-evaluated 100 times for optimal cost based on the mean value of selected parameter sets. The results are displayed on a histogram. One could observe that the re-simulated optimal costs are still very close to zero which is also close to the best fitting cost.

With additional information from these two figures, users are ensured that the performed statistical analysis and the interpretation based on the mean value are also reasonable for case study 2. Nevertheless, since not all parameters in our case studies are identifiable (e.g., in case study 4), we reported the results from the best run of each case study in the manuscript.

In case the mean value is used as a mean for interpretation, *optPBN* also has a wrap-up function of the results from ‘bestRunsStat’ function (not shown). The output of the function displays the rules of each model case together with the corresponding means (cijMeans) and standard deviations (cijStds). The results from the wrap-up function for the fourth model case are shown in the blue box below.

```
Report_Fitted_exp4 =

   'Rules'                   'Mean'          'S.D.'

   'PI3K = 1'                [        1]     [        0]

   'PTEN = 1'                [        1]     [        0]

   'PIP3 = PI3K'             [   0.6017]     [   0.0098]

   'PIP3 = PTEN'             [7.8984e-06]    [7.7773e-05]

   'PIP3 = PI3K & ~PTEN'     [   0.3983]     [   0.0098]

   'PIP3 = 0'                [8.5107e-06]    [1.7038e-04]
```

According to the results from the wrap-up function, the mean selection probability of the first Boolean function (PIP3=PI3K) is 0.6017 with the respective standard deviation of 0.0098. Therefore, this can be interpreted that the weight of PIP3 activation solely by PI3K mostly varies in the range of 59% to 61% (range of mean +/- 1 S.D.). In parallel, the weight of the third Boolean function, ‘PIP3=PI3K & ~PTEN’, should be mostly varied in the range of 39% to 41% (mean: 0.3983, S.D.: 0.0098). The remaining two Boolean functions have very low mean values (practically zero) and high standard deviation values comparing to the corresponding mean values. This indicates that the interpretation from bestRun and from the mean of fitted parameter sets are resembled once parameters are all clustered together like in this case study.

In addition, the selection probabilities from the best parameter sets or the mean and S.D. values from the fitted parameter sets (once their distributions are not sparse) help to determine which Boolean interaction(s) is/are relevant in the context of study. Once the value of selection probability is approaching zero e.g. in the case of $C_2^{(PIP3)}$ and cij4, $C_4^{(PIP3)}$, it suggests that these two Boolean rules, which reflect the types and sources of interaction, might not be relevant in the context of study and therefore should not be included in the model. In contrast, non-zero values of selection probabilities e.g., in the case of $C_1^{(PIP3)}$ and $C_3^{(PIP3)}$ with low standard deviations comparing to their mean values indicate that these two Boolean rules, which reflect other types and sources of interaction, are important to explain the given experimental data. Thus, these interactions are relevant in the context of our study.

There is an additional final remark on the quality of steady-state probability approximation, we found that increasing number of selection probability parameter samplings, increasing accuracy (via adjusting 'r' in the main pipeline and adjusting 'nsteps' in the alternative approach) and increasing number of iterations (only in the alternative approach) can reduce the variability of results and can also improve the approximation of steady-state distributions towards the exact solution. However, such assignments will also increase the computational time accordingly (see more analysis in Supporting Information: File S6).

Additional function 'shuffleCijs' for qualitative assessment of all assigned Boolean rules

Apart from the functions to perform optimisation and statistical analyses on PBN model as shown previously, there is also an additional function which allows for a rapid identification and relevancy assessment of all assigned Boolean rules in qualitative manner (see the 'shuffleCijs' function at the end of the pipeline script). To describe the functionality of this function in detail, the original version of toy model from Saez-Rodriguez *et al.* [1] which contains discrete state values (0 or 1) is demonstrated.

In this setting, we are not interested in determining parameter values quantitatively as in case study 2. On the other hand, we are interested in finding all possible Boolean rules from the given set which correspond to the measurement data. Because of the qualitative nature of measurement data (see Supporting Information: Figure S1), there is a high chance that there will be more than one corresponding Boolean function that could equally match the experimental data. In parallel, it is possible that there would be just only one possible answer identified during the optimisation process while the other possibilities are ignored. Therefore, the function 'shuffleCijs' is introduced to solve this problem.

```
%% Perturb estimated cijMeans per state and calculate correct means
[cijMeansShuff,cijStdsShuff]=shuffleCijs(cijMeans,bestxMean,'perState',estim.
thresh);
```

The principal concept of the 'shuffleCijs' function is to pass the input from 'bestxMean' parameter (see the explanation in the previous section) to all Boolean rules in all possible orders. Then, the Boolean rules with newly converted 'cij' values are subsequently re-assessed for optimal costs. This process is performed in order to check whether the identified 'cij' value of one Boolean rule can also match the experimental data by apply the same 'cij' value to the other Boolean rules or not. If there are more than one corresponding Boolean rules for one molecule that pass the defined threshold (estim.thresh), the 'cijMeansShuff' parameter will show the equally distributed 'cij' values for each of the possible answer with the respective standard deviations in 'cijStdsShuff' parameter as outputs (not shown).

To make the explanation simpler, the figure below on the left shows the 'cijMeans' and 'cijStd' values of NFkB (first column) and of ERK (second column) from the analyses on the original toy model of Saez-Rodriguez *et al.* [1] As seen, there are two corresponding Boolean rules for ERK with unequal probabilities where it could be interpreted that only the first Boolean rule corresponds to the data.

```
mean:
          0    0.9690
     1.0000         0
          0    0.0310
          0         0
          0         0
std:
          0    0.1435
          0         0
          0    0.1435
          0         0
          0         0
```

By applying the 'shuffleCijs' function, the 'cijMeansSuff' parameter reveals the results of 0.5, 0, 0.5, 0, 0 on the second column which infer that there are indeed two possible answers (Explanation: the sum of all parameter values (1) is divided by 2 and yield 0.5 for the two corresponding functions, see the figure on the right).

```
EXPERIMENT6:
mean:

          0    0.5000
     1.0000         0
          0    0.5000
          0         0
          0         0
```

References:

1. Saez-Rodriguez J, Alexopoulos LG, Epperlein J, Samaga R, Lauffenburger DA, Klamt S, Sorger PK (2009) Discrete logic modeling as a means to link protein signaling networks with functional analysis of mammalian signal transduction. Molecular Systems Biology 5(331): 1-19.
2. Schlatter R, Schmich K, Vizcarra IA, Scheurich P, Sauter T, Borner C, Ederer M, Merfort I, Sawodny O (2009) ON/OFF and Beyond – A Boolean Model of Apoptosis. PLoS Computational Biology 5(12): e1000595.
3. Miranda EN, Parga N (1989) Noise effects in the kauffman model. Europhysics Letters 10:293–298.
4. Shmulevich I, Gluhovsky I, Hashimoto RF, Dougherty ER, Wei Zhang (2003) Steady-state analysis of genetic regulatory networks modelled by probabilistic Boolean networks. Comparative and Functional Genomics 4: 601-608.
5. Raftery AE, Lewis S (1991) How many iterations in the Gibbs sampler?. In: Bayesian Statistic 4: Proceedings of the Fourth Valencia International Meeting. Berger JO *et al.*, editors. Oxford: Oxford University Press. pp. 763-773.
6. BN/PBN Toolbox [http://code.google.com/p/pbn-matlab-toolbox]
7. Vaz AIF, Vicente LN (2007) A particle swarm pattern search method for bound constrained global optimization. Journal of Global Optimization 39(2): 197-219.
8. Schmidt H, Jirstrand M (2006) Systems Biology Toolbox for MATLAB: A computational 5.platform for research in Systems Biology. Bioinformatics 22(4): 514-515.
9. Systems Biology Toolbox 2 [http://www.sbtoolbox2.org]
