# Supplementary material for: optPBN: An Optimisation Toolbox for Probabilistic Boolean Networks
Source: PLoS One. 2014 Jul 1;9(7):e98001. doi: 10.1371/journal.pone.0098001 (PMC4077690; doi:10.1371/journal.pone.0098001)
Supplement: File S4 — Grid-based pipeline of optPBN toolbox. The PDF documentation provides a description on the grid-based pipeline of the optPBN toolbox. It also describes a strategy to combine two optimisation algorithms, evolutionary algorithm (EA) and differential evolution algorithm (DE), as a single optimiser. An optimisation run on Grid'5000 is demonstrated as an example. (PDF) [file pone.0098001.s005.pdf]

The grid-based pipeline of optPBN toolbox

The grid-based version of *optPBN* toolbox allows having solutions evaluated seamlessly in parallel on remote machines, thus bringing a brute force computational power speed-up. The execution time gained by running the algorithms on a cluster or on a grid mainly depends on the number of available resources. Furthermore, both the evolutionary algorithm (EA) and the differential evolution algorithm (DE) are run in separate threads and exchange solutions in asynchronous manner in order to gain a rapid convergence of solutions.

During an optimisation run, each of the two algorithms sends solutions for evaluation to remote nodes. After a predefined number of iterations, the nodes enter into a waiting state and a subset of selected population is sent to the other algorithm. Next, the incoming solutions are integrated in the local population via a replacement strategy that guarantees a constant population size. All communication, i.e., the sending of data over the network, is managed in a transparent manner by the ParadisEO framework via message passing interface (MPI) primitives. Once the replacement is done and the fitness of each solution is received, the algorithms resume and continue their execution locally.

It should be noted that the communication steps are not synchronized between the two algorithms. For instance, DE could carry the evaluation step while EA is undergoing selection. Also, the remote evaluation of the candidate solutions is done with no synchronization or other

dependency relationship. This allows the time-discrepancies among different evaluations to have only a minor impact on the total execution time. Once a remote node finished evaluating a solution, it signals its state as available thus allowing new tasks to be sent to it. For any additional information regarding this paradigm, please consult [1].

The behind-the-scene logical structure of the grid-based application consists of several nodes including a master node, a scheduler and worker (slave) nodes. The master node is used in this specific setup to run both algorithms. The scheduler (completely transparent from a user point of view) is used to manage the available pool of resources. Namely, it receives task requests and the data that needs to be transferred to the slave nodes. Then, it distributes the workload and sends back the results once the parallel evaluation is completed.

All logical nodes can be deployed on a single or on different physical machines. In both cases, a series of threads are spawned in order to take advantage of multi-core architectures. The exact mapping of the logical nodes to physical machines is controlled via the underlying MPI ‘infrastructure’ as directed by an XML file called schema.xml. The ‘runner’ entries, as assigned in the code, specify what algorithms to execute, e.g. 1 for the first algorithm, 2 for the second and so forth. Next, a ‘scheduler’ entry specifies on which node the scheduling algorithm should be placed. In the last step, a list of worker nodes is assigned, generally by allowing a one-to-one mapping with the physical machines, in order to execute the task. A summarized workflow of this process on physical machines is given below.

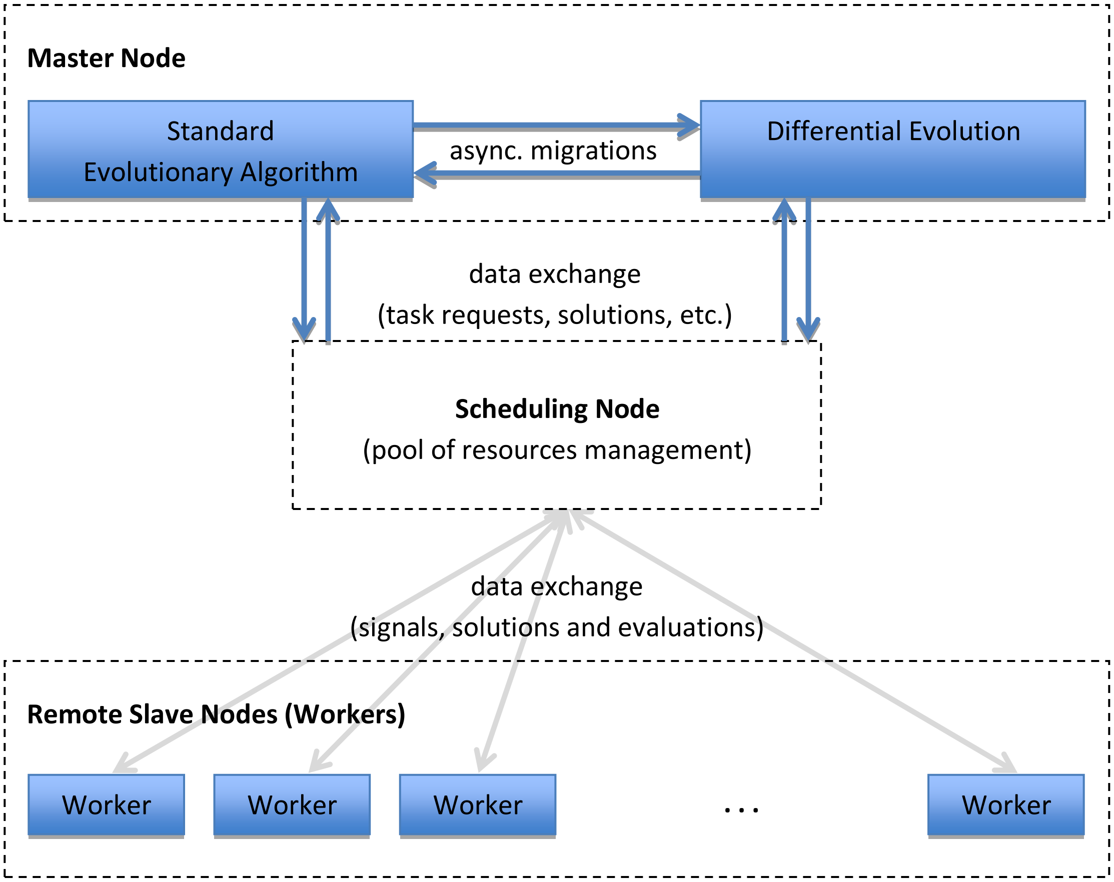
Master Node
Standard
Evolutionary Algorithm
async. migrations
Differential Evolution
data exchange
(task requests, solutions, etc.)
Scheduling Node
(pool of resources management)
data exchange
(signals, solutions and evaluations)
Remote Slave Nodes (Workers)
Worker
Worker
Worker
. . .
Worker

Grid-based optimisation by optPBN on Grid'5000 (G5K)

To illustrate the pipeline of *optPBN* toolbox on a grid-based infrastructure, we introduce Grid'5000 (G5K), a large scale nation-wide infrastructure for grid research in France and collaborating countries, as an example. Currently, there are 9 sites in France (Bordeaux, Grenoble, Lille, Lyon, Nancy, Reims, Rennes, Sophia, Toulouse), one site in Porto Alegre in Brazil, and one site in Luxembourg. The initial aim of G5K in 2003 was to reach 5000 processors in the platform. It has been later reframed at 5000 cores, and was reached during winter 2008-2009. [2]

In the grid-based *optPBN* pipeline, we classified the process into 3 steps, pre-processing, grid-based execution, and post-processing. An overview of the grid-based pipeline is given in the following diagram.

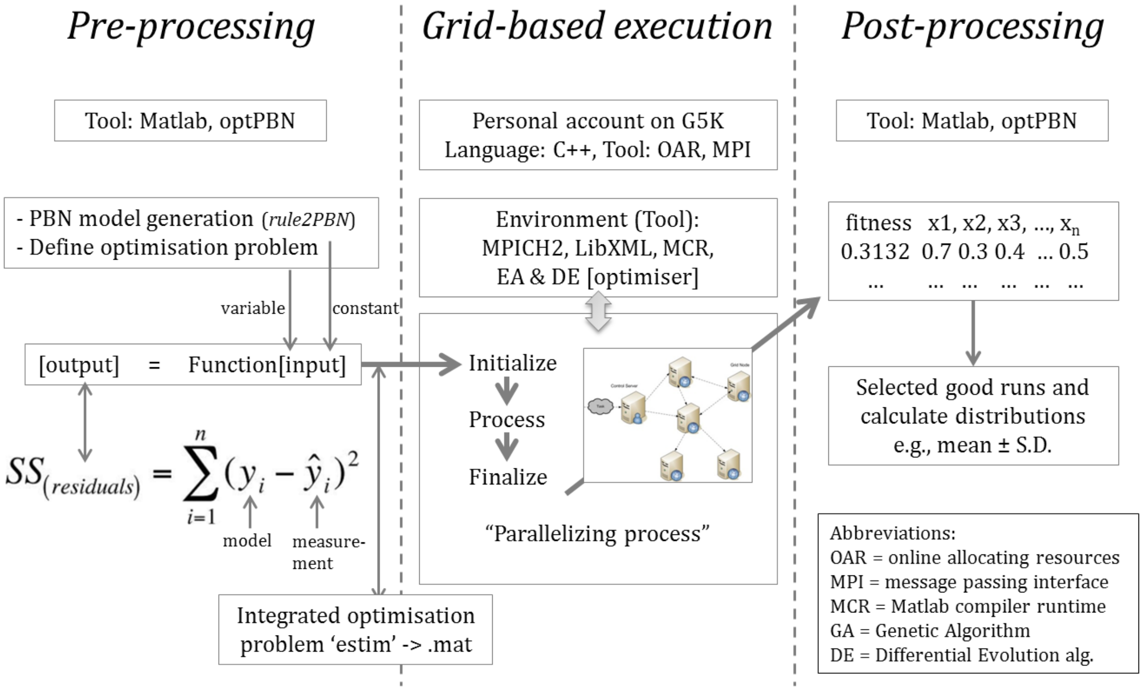

In the pre-processing step, a preliminary model structure and measurement data are used as the inputs. The scripts *rule2PBN, add2estim* and *preprocessMultiExp* (in the stand-alone version of *optPBN* toolbox) are applied to combine the two inputs into an integrated optimisation problem which is stored as a Matlab global data structure called 'estim'. The function used to evaluate the fitness quality is sum of square error (SSE or SS) where model structure is defined as constant input and the sampled parameters which represent selection probabilities in PBNs are defined as variable input. After 'estim' is generated on a local computer, it is saved as a matrix file (.mat) which can be transferred to be solved on a cluster or a grid-based infrastructure such as Grid'5000.

Prior to proceeding with the grid-based execution step, users have to set-up the grid-based version of *optPBN* toolbox on a cluster or a grid-based infrastructure. The installation guide is provided in Supporting Information: File S8. Once the toolbox is properly installed, user can reserve computation resource to run the optimisation task (please refer to the documentation on resource reservation of the respective cluster or grid infrastructure). For the operation on Grid'5000, an example of node reservation and the execution of the script are provided in Supporting Information: File S8 (step #9). A snapshot of the commands to boot up and to execute slave nodes is shown below.

```
ptrairatphisan@helios-11:~/optPBNInstall/workspace/sysb-opt/src$ mpdboot -n 10 -f machines --rsh=/usr/bin/
oarsh
ptrairatphisan@helios-11:~/optPBNInstall/workspace/sysb-opt/src$ mpiexec -n 40 ./sysbexample @alg.param Ap
optosis_Reduced_G5K_NEW_NormTo2_OptCpx2_Rev2.mat
```

As described in the previous section, the grid-based optimisation is performed in parallelising manner. This process includes 3 important steps: Initiation – to deploy

optimisation algorithms (EA and DE) on the grid, Process – to perform parallel evaluation of the optimisation algorithm for individual solution, and Finalize – to collect the results, then combine and report back to users. A snapshot of the first results from the optimisation process by grid-based *optPBN* pipeline is shown as follow.

```
#1 [ EXEC ]: Starting... Starting the algorithm...      Best    Average & Stdev
1       1.02023 2.86581 0.854243
Starting the algorithm...       Best    Average & Stdev
1       0.994852        2.60806 0.860451
2       1.02023 2.85701 0.852207
2       0.730013        2.12652 0.798906
3       1.02023 2.85424 0.854091
3       0.726175        1.74515 0.746433
4       1.01599 2.84121 0.857737
4       0.552534        1.47274 0.637391
5       1.01599 2.83933 0.85846
5       0.464666        1.24133 0.559726
0 : 0.464666  19 0.358301 0.254056 0.70225 0 0.152692 0.12691 0 0.602846 0 0 0.474674 0.545911 0.67992 0.3
26331 0.317106 0.719662 0.158391 0.560978 0.242531
```

Once the optimisation process is finished in each iteration, the results which comprised fitness cost and sampled parameter set are stored in the first column and the successive column(s) of the result file accordingly. There are two result files as the outcome of the two optimisation algorithms, EA_output.log and DE_output.log. These result files will be retrieved from the cluster/grid to be further analysed in the post-processing step on a local computer.

In the post-processing step, the quality of model fitting can be checked by using the script *evalCijAndSimulate_G5K* (already integrated in the stand-alone version of *optPBN* toolbox) to re-simulate model states based on the best parameter sets and to compare them side-by-side with measurement data. Furthermore, a set of parameters with good fitness can be selected to perform statistical analysis by calculating mean and standard deviation using the script *BestRunsStat*. The results from such analysis can be further interpreted as described in the manuscript which might shed some new insights into biological networks.

Reference:

1. Storn R, Price K (1997) Differential Evolution – A Simple and Efficient Heuristic for Global Optimization over Continuous Spaces. Journal of Global Optimization 11: 341-359.

2. Grid'5000 official website [https://www.grid5000.fr]
