# Supplementary material for: optPBN: An Optimisation Toolbox for Probabilistic Boolean Networks
Source: PLoS One. 2014 Jul 1;9(7):e98001. doi: 10.1371/journal.pone.0098001 (PMC4077690; doi:10.1371/journal.pone.0098001)
Supplement: File S5 — Derivations of the two-state Markov chain approach's formulas. The PDF documentation provides the corrected derivations of the two-state Markov chain approach's formulas in relation to the derivations presented in the original work of Raftery and Lewis [30]. The term λ which refers to in the calculation of m 0 should be replaced with (i.e., ). In addition, Φ which denotes the standard normal cumulative distribution function in the calculation of N should be substituted with the inverse of its function, Φ−1, after derivations. (PDF) [file pone.0098001.s006.pdf]

Two-state Markov chain approach

1 Derivation of the number of "burn-in" iterations

Let $\{Z_t\}_{t\geq 0}$ be a discrete-time two-state Markov chain as given in Figure 1. Z_t has the value 0 or 1 if the system is in state 0 or state 1 at time t, respectively. The transition probabilities satisfy $0 < \alpha, \beta < 1$ and the transition matrix for this chain has the following form

$$P = \begin{bmatrix} 1-\alpha & \alpha \\ \beta & 1-\beta \end{bmatrix}.$$

The chain is ergodic and the steady-state distribution is $\pi = [\pi_0 \ \pi_1] = [\frac{\beta}{\alpha+\beta} \ \frac{\alpha}{\alpha+\beta}]$. Let $\mathbb{E}_\pi(Z_n)$ denote the expected value of Z_n, with respect to the steady-state distribution π. Then, $\mathbb{E}_\pi(Z_n) = \frac{\alpha}{\alpha+\beta}$.

The l-step transition matrix can be written, as can be checked by induction, in the form

$$P^l = \begin{bmatrix} \pi_0 & \pi_1 \\ \pi_0 & \pi_1 \end{bmatrix} + \frac{\lambda^l}{\alpha+\beta} \cdot \begin{bmatrix} \alpha & -\alpha \\ -\beta & \beta \end{bmatrix},$$

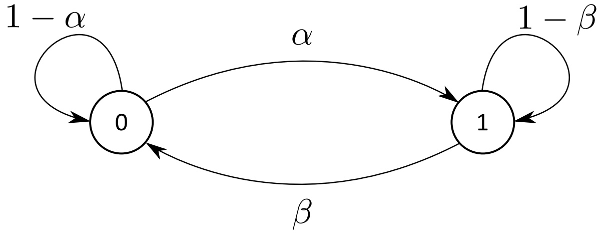

Figure 1: Two-state ergodic Markov chain.

where $\lambda = (1-\alpha-\beta)$. Matrix P has two distinct eigenvalues: 1 and λ, and $|\lambda| < 1$.

Suppose we require m to be such that the following condition is satisfied

$$\left| \begin{bmatrix} P[Z_m = 0 \,|\, Z_0 = j] \\ P[Z_m = 1 \,|\, Z_0 = j] \end{bmatrix} - \begin{bmatrix} \pi_0 \\ \pi_1 \end{bmatrix} \right| < \begin{bmatrix} \epsilon \\ \epsilon \end{bmatrix}$$

for some $\epsilon > 0$. If $e_0 = [1\ 0]$ and $e_1 = [0\ 1]$, then for $j = 0, 1$ we have that

$$\begin{bmatrix} P[Z_m = 0 \,|\, Z_0 = j] \\ P[Z_m = 1 \,|\, Z_0 = j] \end{bmatrix} = e_j P^m.$$

With this, the above requirement can be written as

$$\left| e_j \left(\begin{bmatrix} \pi_0 & \pi_1 \\ \pi_0 & \pi_1 \end{bmatrix} + \frac{\lambda^m}{\alpha+\beta} \cdot \begin{bmatrix} \alpha & -\alpha \\ -\beta & \beta \end{bmatrix} \right) - \begin{bmatrix} \pi_0 \\ \pi_1 \end{bmatrix} \right| < \begin{bmatrix} \epsilon \\ \epsilon \end{bmatrix}.$$

For $j = 0$ the above simplifies to

$$\left| \frac{\lambda^m}{\alpha+\beta} \cdot \begin{bmatrix} \alpha \\ -\alpha \end{bmatrix} \right| < \begin{bmatrix} \epsilon \\ \epsilon \end{bmatrix}$$

and for $j = 1$ we have

$$\left| \frac{\lambda^m}{\alpha+\beta} \cdot \begin{bmatrix} -\beta \\ \beta \end{bmatrix} \right| < \begin{bmatrix} \epsilon \\ \epsilon \end{bmatrix}.$$

It is enough to consider the following two inequalities

$$\left| \frac{\lambda^m \alpha}{\alpha+\beta} \right| < \epsilon \qquad \text{and} \qquad \left| \frac{\lambda^m \beta}{\alpha+\beta} \right| < \epsilon,$$

which, since $\alpha, \beta > 0$, can be rewritten as

$$|\lambda^m| < \frac{\epsilon(\alpha+\beta)}{\alpha} \qquad \text{and} \qquad |\lambda^m| < \frac{\epsilon(\alpha+\beta)}{\beta}.$$

Equivalently, m has to satisfy

$$|\lambda^m| < \frac{\epsilon(\alpha+\beta)}{\max(\alpha,\beta)}.$$

By the fact that $|\lambda^m| = |\lambda|^m$ this can be expressed as

$$|\lambda|^m < \frac{\epsilon(\alpha+\beta)}{\max(\alpha,\beta)}.$$

Then, by taking the logarithm to base 10 on both sides[1], we have that

$$m \cdot \log(|\lambda|) < \log\left(\frac{\epsilon(\alpha+\beta)}{\max(\alpha,\beta)}\right)$$

and in consequence, since $|\lambda| < 1$ and $\log|\lambda| < 0$,

$$m > \frac{\log\left(\frac{\epsilon(\alpha+\beta)}{\max(\alpha,\beta)}\right)}{\log(|\lambda|)}.$$

2 Derivation of the sample size

To determine the sample size N, we note first that the estimate of $\pi_1 = \mathbb{E}_\pi(Z_n)$ is $\bar{Z}_n = \frac{1}{n}\sum_{t=1}^{n} Z_t$. This holds by the Law of Large Numbers for stationary stochastic processes (often called the Birkhoff ergodic theorem). Under the condition of "ergodicity" it has exactly the same conclusion as the Strong Law of Large Numbers for i. i. d. sequences. Now, by a variant of the Central Limit Theorem for non-independent random variables[2], for n large, $\bar{Z}_n$ is approximately normally distributed with mean $\pi_1 = \frac{\alpha}{\alpha+\beta}$ and variance $\frac{1}{n}\frac{\alpha\beta(2-\alpha-\beta)}{(\alpha+\beta)^3}$, see Section 3. Let $\sigma^2 = \frac{\alpha\beta(2-\alpha-\beta)}{(\alpha+\beta)^3}$ and let X be the standardised $\bar{Z}_n$, i.e.,

$$X = \frac{\bar{Z}_n - \pi_1}{\sigma/\sqrt{n}}.$$

If follows that X is normally distributed with mean 0 and variance 1, i.e., $X \sim N(0,1)$. The requirement that $P[\pi_1 - r \leq \bar{Z}_n \leq \pi_1 + r] = s$ can be rewritten as

$$P[-r \leq \bar{Z}_n - \pi_1 \leq r] = s,$$

[1] In fact, by the formula for change of base for logarithms, the natural logarithm (ln) or logarithm to base 2 ($\log_2$) or to any other base could be used to calculate m instead of log. Notice that m does **not** depend on the choice of the base of the logarithm!

[2] Notice that the random variables Z_t, Z_{t+1} which values are consecutive states of a trajectory are correlated and are not independent.

and further as

$$P[-r \cdot \frac{\sqrt{n}}{\sigma} \leq \frac{\bar{Z}_n - \pi_1}{\sigma/\sqrt{n}} \leq r \cdot \frac{\sqrt{n}}{\sigma}] = s,$$

which is

$$P[-r \cdot \frac{\sqrt{n}}{\sigma} \leq X \leq r \cdot \frac{\sqrt{n}}{\sigma}] = s.$$

Since $X \sim N(0,1)$ and $N(0,1)$ is symmetric around 0, it follows that

$$P[0 \leq X \leq r \cdot \frac{\sqrt{n}}{\sigma}] = \frac{s}{2}$$

and

$$P[X \leq r \cdot \frac{\sqrt{n}}{\sigma}] = \frac{1}{2} + \frac{s}{2} = \frac{1}{2}(1+s).$$

Let $\Phi(\cdot)$ be the standard normal cumulative distribution function. Then the above can be rewritten as

$$\Phi(r \cdot \frac{\sqrt{n}}{\sigma}) = \frac{1}{2}(1+s).$$

Therefore, if we denote the inverse of the standard normal cumulative distribution function with $\Phi^{-1}(\cdot)$, then we have that

$$r \cdot \frac{\sqrt{n}}{\sigma} = \Phi^{-1}(\frac{1}{2}(1+s)).$$

In consequence,

$$n = \frac{\sigma^2}{\left\{\frac{r}{\Phi^{-1}(\frac{1}{2}(1+s))}\right\}^2} = \frac{\frac{\alpha\beta(2-\alpha-\beta)}{(\alpha+\beta)^3}}{\left\{\frac{r}{\Phi^{-1}(\frac{1}{2}(1+s))}\right\}^2}.$$

3 Derivation of the asymptotic variance

By the Central Limit Theorem for stationary stochastic processes $\sqrt{n}(\bar{Z}_n - \mathbb{E}_\pi(Z_n)) \xrightarrow{d} N(0, \sigma^2_{\text{as}})$ as $n \to \infty$, where σ^2_{as} is the so-called asymptotic variance given by

$$\sigma^2_{\text{as}} = \text{Var}_\pi(Z_j) + 2\sum_{k=1}^{\infty} \text{Cov}_\pi(Z_j, Z_{j+k}) \tag{3.1}$$

and $\mathrm{Var}_\pi(\cdot)$ and $\mathrm{Cov}_\pi(\cdot)$ denote the variance and covariance with respect to the steady-state distribution π, respectively. We proceed to calculate σ^2_{as}. First, observe that $\mathbb{E}_\pi(Z_n Z_{n+1}) = \frac{\alpha}{\alpha+\beta}(1-\beta)$: $Z_n Z_{n+1} \neq 0$ if and only if the chain is state 1 at time n and remains in 1 at time $n+1$, i.e., $Z_n = Z_{n+1} = 1$. The probability of this event at steady state is $\frac{\alpha}{\alpha+\beta}(1-\beta)$. Then, by the definition of covariance, we have that the steady-state covariance between consecutive random variables of the two-state Markov chain, i.e., $\mathrm{Cov}_\pi(Z_n, Z_{n+1})$ is

$$\begin{aligned}
\mathrm{Cov}_\pi(Z_n, Z_{n+1}) &= \mathbb{E}_\pi\left[(Z_n - \mathbb{E}_\pi(Z_n))(Z_{n+1} - \mathbb{E}_\pi(Z_{n+1}))\right] \\
&= \mathbb{E}_\pi\left[(Z_n - \frac{\alpha}{\alpha+\beta})(Z_{n+1} - \frac{\alpha}{\alpha+\beta})\right] \\
&= \mathbb{E}_\pi\left[Z_n Z_{n+1} - \frac{\alpha}{\alpha+\beta}(Z_n + Z_{n+1}) + \frac{\alpha^2}{(\alpha+\beta)^2}\right] \\
&= \mathbb{E}_\pi(Z_n Z_{n+1}) - \frac{\alpha}{\alpha+\beta}(\mathbb{E}_\pi(Z_n) + \mathbb{E}_\pi(Z_{n+1})) + \frac{\alpha^2}{(\alpha+\beta)^2} \\
&= \frac{\alpha(1-\beta)}{\alpha+\beta} - 2\frac{\alpha^2}{(\alpha+\beta)^2} + \frac{\alpha^2}{(\alpha+\beta)^2} \\
&= \frac{\alpha\beta(1-\alpha-\beta)}{(\alpha+\beta)^2}.
\end{aligned}$$

Further, we have that $\mathrm{Var}_\pi(Z_n) = \pi_0 \cdot \pi_1 = \frac{\alpha\beta}{(\alpha+\beta)^2}$ (variance of the Bernoulli distribution) and it can be shown that $\mathrm{Cov}_\pi(Z_n, Z_{n+k}) = (1-\alpha-\beta)^k \cdot \mathrm{Var}_\pi(Z_n)$ for any $k \geq 1$. Now, according to Equation (3.1), we have

$$\begin{aligned}
\sigma^2_{\text{as}} &= \mathrm{Var}_\pi(X_j) + 2\sum_{k=1}^{\infty} \mathrm{Cov}_\pi(X_j, X_{j+k}) \\
&= \frac{\alpha\beta}{(\alpha+\beta)^2} + 2\sum_{k=1}^{\infty}(1-\alpha-\beta)^k \cdot \frac{\alpha\beta}{(\alpha+\beta)^2} \\
&= \frac{\alpha\beta}{(\alpha+\beta)^2} + \frac{2\alpha\beta}{(\alpha+\beta)^2} \cdot \sum_{k=1}^{\infty}(1-\alpha-\beta)^k \\
&= \frac{\alpha\beta}{(\alpha+\beta)^2} + \frac{2\alpha\beta}{(\alpha+\beta)^2} \cdot \frac{1-\alpha-\beta}{\alpha+\beta} \\
&= \frac{\alpha\beta(2-\alpha-\beta)}{(\alpha+\beta)^3}.
\end{aligned}$$

In consequence, $\bar{Z}_n$ is approximately normally distributed with mean $\frac{\alpha}{\alpha+\beta}$ and variance $\frac{1}{n}\frac{\alpha\beta(2-\alpha-\beta)}{(\alpha+\beta)^3}$.
