# Supplementary material for: optPBN: An Optimisation Toolbox for Probabilistic Boolean Networks
Source: PLoS One. 2014 Jul 1;9(7):e98001. doi: 10.1371/journal.pone.0098001 (PMC4077690; doi:10.1371/journal.pone.0098001)
Supplement: File S9 — Installation guide for the grid-based pipeline of optPBN and an execution example on Grid'5000. The PDF documentation provides a list of commands to set-up the grid-based version of the optPBN toolbox on a cluster or on a grid-based infrastructure. An example set of commands to reserve resources and to execute an optimisation task on Grid'5000 is also included. (PDF) [file pone.0098001.s010.pdf]

Installation & execution guide for the grid-based version of optPBN toolbox

The grid-based version of *optPBN* toolbox can be downloaded from SourgeForge at https://sourceforge.net/projects/optpbn. Please type the password 'SysbioUL' upon the extraction of the compressed optPBN toolbox (in zip format) if asked.

Once the toolbox is placed on the working directory of a cluster or of a grid-based infrastructure such as Grid'5000, please run the installation file 'installOptPBN.sh' e.g., by using the command './installOptPBN.sh' on Linux operation system. A detailed description of the installation file is provided below.

#1 create the installs and lower level directories, namely for MCR, MPICH2 and LibXML

```
cd ~ && mkdir installs
mkdir installs/mcr
mkdir installs/mpich2
mkdir installs/libxml2
```

#2 copy the paradiseo archive into the installs directory and uncompress

```
cp optPBNInstall/kits/paradiseo-1.1.bz2 installs/ && cd installs
tar xvjf paradiseo-1.1.bz2
```

#3 uncompress and install mpich2, libxml and gsl(located in the lib subfolder)

```
cd paradiseo-1.1/lib

tar xvzf mpich2-1.0.3.tar.gz
cd mpich2-1.0.3
./configure --prefix=$HOME/installs/mpich2/ && make && make install
```

cd .. && tar xvjf libxml2-2.6.0.tar.bz2 && cd libxml2-2.6.0
./configure --prefix=$HOME/installs/libxml2/ && make && make install

!! IMPORTANT !! if your system already provides a libxml library, e.g. in /usr/lib, symbolic links should be created instead
in the ~/installs/libxml2/lib directory (modify the symbolic links already present in ~/installs/libxml2/lib to point to the system library)
the ln command can be used to create symbolic links (-s option); overseeing this step might cause the installation to fail!

cd ~/optPBNInstall/kits
tar xvzf gsl-1.15.tar.gz && cd gsl-1.15
./configure --prefix=$HOME/installs
make && make install

#4 set environment and variables
echo 'export PATH=$HOME/installs/bin:$HOME/installs/mpich2/bin:$HOME/installs/libxml2/bin/:$PATH' >> ~/.bashrc
echo 'export PATH=$HOME/installs/bin:$HOME/installs/mpich2/bin:$HOME/installs/libxml2/bin/:$PATH' >> ~/.bash_profile
echo 'export LD_LIBRARY_PATH=$HOME/installs/lib:$HOME/installs/mpich2/lib/:$HOME/installs/libxml2/lib:$LD_LIBRARY_PATH' >> ~/.bashrc
echo 'export LD_LIBRARY_PATH=$HOME/installs/lib:$HOME/installs/mpich2/lib/:$HOME/installs/libxml2/lib:$LD_LIBRARY_PATH' >> ~/.bash_profile

echo 'export LD_LIBRARY_PATH=$HOME/optPBNInstall/workspace/sysb-opt/src/libs:$LD_LIBRARY_PATH' >> ~/.bashrc
echo 'export LD_LIBRARY_PATH=$HOME/optPBNInstall/workspace/sysb-opt/src/libs:$LD_LIBRARY_PATH' >> ~/.bash_profile

echo 'export LD_LIBRARY_PATH=$HOME/installs/mcr/v78/runtime/glnxa64:$LD_LIBRARY_PATH' >> ~/.bashrc
echo 'export LD_LIBRARY_PATH=$HOME/installs/mcr/v78/runtime/glnxa64:$LD_LIBRARY_PATH' >> ~/.bash_profile

echo 'export LD_LIBRARY_PATH=$HOME/optPBNInstall/workspace/sysb-opt/src/libs:$LD_LIBRARY_PATH' >> ~/.bashrc

echo 'export MCR_INHIBIT_CTF_LOCK=1' >> ~/.bashrc
echo 'export MCR_INHIBIT_CTF_LOCK=1' >> ~/.bash_profile
source ~/.bashrc

echo 'MPD_SECRETWORD=secretw' > ~/.mpd.conf
chmod 600 ~/.mpd.conf

#5 install ParadisEO [version 1.1]: EO+MO+MOEO+PEO
cd ~/installs/paradiseo-1.1/paradiseo-eo/build/
rm -fr * && cmake ../ -Dconfig=$HOME/installs/paradiseo-1.1/install.cmake
make

cd ../../paradiseo-mo/build/
rm -fr * && cmake ../ -Dconfig=$HOME/installs/paradiseo-1.1/install.cmake
make

cd ../../paradiseo-moeo/build
rm -fr * && cmake ../ -Dconfig=$HOME/installs/paradiseo-1.1/install.cmake
make

cd ../../paradiseo-peo/build
rm -fr * && cmake ../ -Dconfig=$HOME/installs/paradiseo-1.1/install.cmake
make

#6 install MCR [choose option 1, i.e. Next, and then specify the /home/{username}/installs/mcr directory, choose Next when prompted, end with Finish]
cd ~/optPBNInstall/kits
chmod +x MCRInstaller.bin
./MCRInstaller.bin -console

#7 compile the project
cd ~/optPBNInstall/workspace/sysb-opt/src/libs/source/example/src
chmod +x compile.grid && ./compile.grid
cp example.exports libexample.ctf libexample.exports libexample.h libexample.so ../../../
cd ~/optPBNInstall/workspace/sysb-opt

./autogen.sh --with-EOdir=$HOME/installs/paradiseo-1.1/paradiseo-eo/ --with-MOdir=$HOME/installs/paradiseo-1.1/paradiseo-mo/ --with-MOEOdir=$HOME/installs/paradiseo-1.1/paradiseo-moeo --with-paradisEOdir=$HOME/installs/paradiseo-1.1/paradiseo-peo

make

#8 run once to extract the .ctf contents
cd src
./sysbexample

#9 Grid5000 ONLY:
make a reservation
oarsub –I –l /nodes=10,walltime=0:90

#verify the nodes
cat $OAR_FILE_NODES | uniq > machines
for i in `cat machines`; do oarsh $i 'hostname -f'; done

#start the MPI daemons
mpdboot -n 10 -f machines --rsh=/usr/bin/oarsh

#adjust the schema.xml file if needed as to match the number of resources and run (in this case using 80 processes)
#note that the node indexing starts at 0; the last node has in this case the label 79

#execute MPI and record computational time
{ time mpiexec -n 80 ./sysbexample @alg.param estim_Case_study_4_Extended.mat; } 2>&1 | tee TimeOutput.log

#shutdown the MPI daemons (after complete the optimisation task)
mpdallexit

The installation process is mostly automated. There is only one step (Step #6: Install Matlab Compiler Runtime, MCR) that requires user's interaction to set the installation path. If you install the toolbox on Grid'5000 (registration for an account required), you can proceed with an example of resources reservation and execution of an optimisation task by following the commands in step #9.
